# Proteomic insights into the associations between obesity, lifestyle factors, and coronary artery disease

**DOI:** 10.1186/s12916-023-03197-8

**Published:** 2023-12-05

**Authors:** Fangkun Yang, Fengzhe Xu, Han Zhang, Dipender Gill, Susanna C. Larsson, Xue Li, Hanbin Cui, Shuai Yuan

**Affiliations:** 1 Department of Cardiology, First Affiliated Hospital of Ningbo University (Ningbo First Hospital), School of Medicine, Ningbo University, 59 Liuting Road, Ningbo, 315010 China; 2 Key Laboratory of Precision Medicine for Atherosclerotic Diseases of Zhejiang Province, Ningbo, China; 3 Cardiovascular Disease Clinical Medical Research Center of Ningbo, Ningbo, Zhejiang China; 4 Key Laboratory of Growth Regulation and Translational Research of Zhejiang Province, School of Life Sciences, Westlake University, Hangzhou, China; 5 Department of Big Data in Health Science School of Public Health, Center of Clinical Big Data and Analytics of The Second Affiliated Hospital, Zhejiang University School of Medicine, Hangzhou, China; 6 Department of Epidemiology and Biostatistics, School of Public Health, Imperial College London, London, UK; 7 Unit of Cardiovascular and Nutritional Epidemiology, Institute of Environmental Medicine, Karolinska Institutet, Stockholm, Sweden; 8 Unit of Medical Epidemiology, Department of Surgical Sciences, Uppsala University, Uppsala, Sweden

**Keywords:** Blood protein, Coronary artery disease, Lifestyle factor, Mediation, Mendelian randomization, Obesity

## Abstract

**Background:**

We aimed to investigate the protein pathways linking obesity and lifestyle factors to coronary artery disease (CAD).

**Methods:**

Summary-level genome-wide association statistics of CAD were obtained from the CARDIoGRAMplusC4D consortium (60,801 cases and 123,504 controls) and the FinnGen study (R8, 39,036 cases and 303,463 controls). Proteome-wide Mendelian randomization (MR) analysis was conducted to identify CAD-associated blood proteins, supplemented by colocalization analysis to minimize potential bias caused by linkage disequilibrium. Two-sample MR analyses were performed to assess the associations of genetically predicted four obesity measures and 13 lifestyle factors with CAD risk and CAD-associated proteins’ levels. A two-step network MR analysis was conducted to explore the mediating effects of proteins in the associations between these modifiable factors and CAD.

**Results:**

Genetically predicted levels of 41 circulating proteins were associated with CAD, and 17 of them were supported by medium to high colocalization evidence. PTK7 (protein tyrosine kinase-7), RGMB (repulsive guidance molecule BMP co-receptor B), TAGLN2 (transgelin-2), TIMP3 (tissue inhibitor of metalloproteinases 3), and VIM (vimentin) were identified as promising therapeutic targets. Several proteins were found to mediate the associations between some modifiable factors and CAD, with PCSK9, C1S, AGER (advanced glycosylation end product-specific receptor), and MST1 (mammalian Ste20-like kinase 1) exhibiting highest frequency among the mediating networks.

**Conclusions:**

This study suggests pathways explaining the associations of obesity and lifestyle factors with CAD from alterations in blood protein levels. These insights may be used to prioritize therapeutic intervention for further study.

**Supplementary Information:**

The online version contains supplementary material available at 10.1186/s12916-023-03197-8.

## Background

Coronary artery disease (CAD) is charactered by the accumulation of atherosclerotic plaque in the epicardial arteries. This process can be modified by lifestyle factors, pharmaceutical treatments, and invasive interventions [1, 2]. Despite advances in the prevention and treatment strategies, CAD remains a major public health challenge and a leading global cause of morbidity and mortality [3]. In 2019, there were ~200 million cases of acute myocardial infarction, angina, and asymptomatic ischemic heart disease globally, leading to 9.1 million deaths and 182.0 million disability-adjusted life years [4]. Despite pharmacological treatments, such as antiplatelet agents, anticoagulants, statins, and other lipid-lowering medications, CAD still has a significant burden, and medical treatment is further associated with adverse effects, including impaired glycemic control, myopathy, hepatotoxicity, and renal toxicity, which are associated with a significant adverse effect burden and non-concordance with treatment [2]. Hence, there is a pressing need to identify potential novel targets for the disease.

Circulating proteins can serve as diagnostic markers and therapeutic targets. Previous studies have revealed the potential involvement of circulating proteins in the progression of CAD, offering new avenues for therapeutic intervention [5]. A previous proteomic analysis identified numerous proteins, and intricate networks and pathways, associated with early atherosclerosis. Of particular interest, a plasma multiplex assay consisting of 13 proteins, selected based on strong associations with atherosclerosis, exhibited robust predictive power for angiographically defined CAD [5]. Another proteomic investigation involving 528 individuals without prior cardiovascular disease suggested that epidermal growth factor receptor and contactin-1 exhibited associations with CAD [6]. However, whether the strategic targeting of these circulating proteins can effectively translate into tangible clinical advantages in mitigating cardiovascular events remains unanswered.

In recent years, there has been a steady movement towards unraveling the genetic underpinnings of CAD, resulting in the identification of approximately 60 distinct genetic loci [3]. These genetic loci encompass various physiological pathways, including but not limited to low-density lipoprotein (LDL) cholesterol and lipoprotein(a) (such as *APOB*, *PCSK9*, *LDLR*, etc.) and triglyceride-rich lipoproteins (including *LPL*, *APOA5*, *ANGPTL4*, etc.) as well as inflammation-related pathways (like *IL6R* and *CXCL12*). However, uncertainties still surround the potential pathways associated with CAD [7]. Additionally, various modifiable factors have been reported to influence cardiovascular health, including smoking, alcohol drinking, coffee consumption, physical activity, sedentary behavior, sleep, diet, and obesity [8]. The implementation of healthy lifestyle behaviors and weight management improves the cardiovascular outcomes, serving as an additional and complementary strategy to the secondary prevention therapy [2]. Circulating proteins may play an important mediating role in the association between obesity and lifestyle factors and the disease [9, 10]. For example, circulating nephronectin was reported to be an actionable mediator of the effect of obesity on COVID-19 severity [11]. However, few studies have been conducted to examine the proteomic pathways linking obesity and lifestyle factors to CAD risk. An appraisal of these associations may facilitate mechanistic understanding of modifiable risk of CAD and possibly generate therapeutic targets among high-risk populations with clinical implications.

The application of Mendelian randomization (MR), specifically leveraging genetic variants predicting the concentration of circulating proteins, can strengthen causal inference [12]. Leveraging genetic variants randomly allocated at conception as an instrumental variable (IV) for the exposure, it becomes feasible to obtain estimates that are less vulnerable to the influence of environmental confounders and reverse causality [13]. In our prior study employing proteome-wide MR and colocalization analysis, we have conducted an extensive investigation to identify potential therapeutic targets for inflammatory bowel disease [14]. Furthermore, our previous studies furnished compelling genetic evidence establishing potential causal associations between several lifestyle factors and CAD [15–17]. Here, we conducted a study to identify blood proteins associated with CAD by employing a proteome-wide MR approach and further explored the mediating network involving obesity, lifestyle factors, proteins, and CAD, thereby contributing to a deeper understanding of the pathogenesis.

## Methods

### Study design

**Fig. 1:**
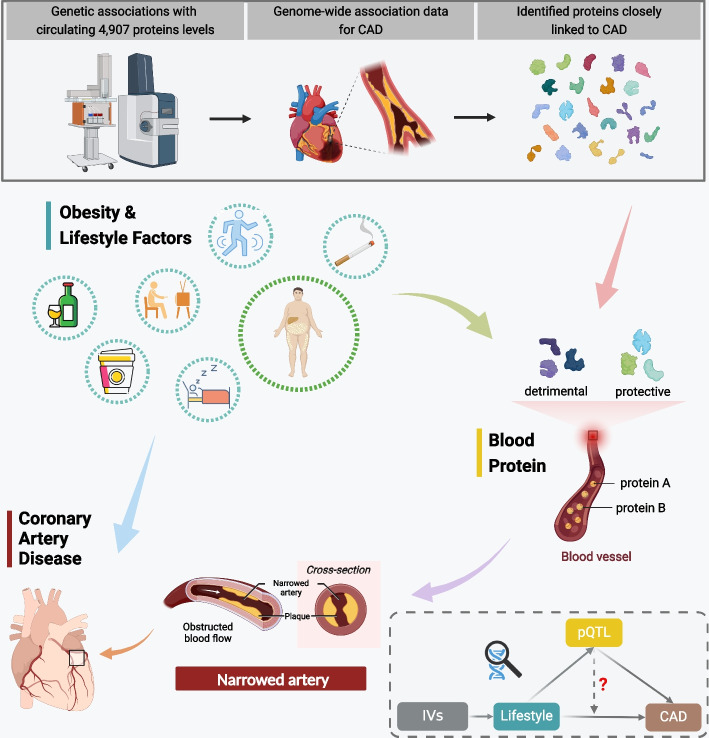
Schematic overview of the study design. Abbreviations: CAD, coronary artery disease; IVs, instrumental variables

A comprehensive investigation employing an integrated genetic approach was designed (Fig. 1). We first obtained genetic instruments for circulating proteins and utilized a proteome-wide MR approach to examine the potential associations between circulating proteins and the risk of CAD. Then, those protein-CAD associations were tested in colocalization analysis. Second, we conducted the two-sample MR analyses to assess the associations of genetically predicted four obesity measures and 13 modifiable factors with CAD risk. Third, the two-step network MR analyses were performed to delve into the potential mediation of CAD-associated proteins in the associations between these modifiable factors and CAD. The included genome-wide association studies (GWASs) had obtained the necessary ethical approvals from the relevant committees, and written informed consent was obtained from all individuals involved in these studies. The datasets employed in the present study have been publicly accessible, without any personally identifiable information. This study was conducted in accordance with the STROBE-MR checklist (Additional file 1) [18].

Two-step MR analysis have some identical assumptions as the two-sample MR analysis although the assumptions should be met for both steps. In detail, the validity of two-step MR results relies on three fundamental assumptions: (i) relevance assumption, i.e., the genetic variants should exhibit a strong association with the exposure of interest; (ii) independence assumption, i.e., the genetic variants should be independent of confounders; and (iii) exclusion restriction, i.e., the genetic variants should solely impact the outcome through the exposure. To satisfy the first assumption, our IV selection was confined to single nucleotide polymorphisms (SNPs) achieving the genome-wide significance threshold. The second assumption is usually satisfied and a merit of the MR approach since genetic variants are randomly assorted at conception and therefore not associated with confounders (e.g., environmental and self-adopted factors). For the third assumption, this assumption is likely to be satisfied in the proteome-wide MR analysis since we selected *cis*-SNPs with few pleiotropic effects as IVs. For MR analysis on modifiable factors, we conducted several supplementary analyses to fortify the resilience of the primary results as well as to detect and correct for potential horizontal pleiotropy. Two-step MR has two additional assumptions that are linearity without interaction and homogeneity of causal effects [19, 20], even though somehow heterogeneity of causal effects may not lead to bias in estimates due to the average effects it assesses [19]. Specifically, the effects of the exposure on the mediator and the effects of the mediator on the outcome should be linear without interactions [17].

### Proteomic data source

We obtained summary-level statistics from a comprehensive protein quantitative trait loci (pQTL) study conducted in a population of 35,559 individuals of Icelandic descent, which encompassed a wide range of genetic associations with the levels of 4,907 circulating proteins (https://www.decode.com/summarydata/) [21]. The measurement of plasma samples was conducted using the SomaScan proteomics platform (Version 4; SomaLogic), which incorporates a collection of over 5000 aptamers, capable of assessing the relative binding of the plasma sample in terms of relative fluorescence units. The aptamers underwent adjustment for age and sex by applying an adjusted rank-inverse normal transformation to their levels. Subsequently, the residuals were also subjected to standardization using rank-inverse normal transformation and served as phenotypes in the genome-wide association analyses. More details could be found elsewhere [21]. In this study, we focused on circulating proteins owning at least one lead *cis*-function genetic variant at the genome-wide threshold.

### Obesity and lifestyle factor data sources

Considering the substantial importance of lifestyle modification and body weight management in clinical practice, we acquired genetic associations with a wide range of modifiable factors from large-scale international consortia and GWASs (Table 1; Additional file 2: Table S1). Specifically, our investigation encompassed smoking initiation, lifetime smoking index, alcohol drinking and dependence, coffee and caffeine consumption, physical activity, sedentary behavior, sleep duration, insomnia, and obesity measures including body mass index (BMI), waist circumference (WC), waist-to-hip ratio (WHR), and visceral adipose tissue (VAT). Genetic IVs for these factors were constructed by selecting single nucleotide polymorphisms (SNPs) identified at the genome-wide significance threshold (*p* < 5 × 10^−8^) and in low linkage disequilibrium (*r*^2^ < 0.01). The data sources of used GWAS datasets and detailed definitions of modifiable factors were provided in the Additional file 2: Table S1-S3.

**Table 1:** Data sources for circulating proteins, obesity measures, lifestyle factors, and coronary artery disease

**Trait**	**Participants**	**Ancestry**	**Unit**	**PMID**
Circulating protein	35,559 individuals	European	SD	34857953
CAD (Consortia)	60,801 cases; 123,504 controls	~80% European	Odds ratio	26343387
CAD (FinnGen)	39,036 cases; 303,463 controls	European	Odds ratio	/
Body mass index	806,834 individuals	European	SD (~ 4.8 kg/m^2^)	30239722
Waist-to-hip ratio	806,834 individuals	European	SD	30239722
Visceral adipose tissue	325,153 individuals	European	1 kg	31501611
Waist circumference	224,459 individuals	European	SD	25673412
Smoking initiation	1,232,091 individuals	European	SD in prevalence of smoking initiation	30643251
Lifetime smoking index	462,690 individuals	European	SD change of lifetime smoking index	31689377
Alcohol drinking	941,280 individuals	European	SD increase of log-transformed alcoholic drinks/week	30643251
Alcohol dependence	46,568 individuals	European	SD in the prevalence of alcohol dependence	30482948
Coffee consumption	375,833 individuals	European	50% change	31046077
Caffeine consumption	9876 individuals	European	80 mg increase (equivalent to dose from 1 cup of coffee)	27702941
Sports	124,842 cases; 225,650 controls	European	≥ 2–3 versus 0 day/week	29899525
MVPA	661,399 individuals	European	“Active” versus “inactive” (≥ 20 mins/week)	36071172
VPA	98,060 cases; 162,995 controls	European	≥ 3 versus 0 day/week	29899525
Sedentary behavior	408,815 individuals	European	SD (~1.5 h) change of leisure television watching time	32317632
Leisure screen time	661,399 individuals	European	SD (hours/day)	36071172
Sleep duration	446,118 individuals	European	60 min	30846698
Insomnia	397,959 cases; 933,057 controls	European	SD in the prevalence of insomnia	30804565

*Abbreviations*: *CAD* Coronary artery disease, *MVPA* Mzoderate to vigorous physical activity, *SD* Standard deviation, *VPA* Vigorous physical activity

### Outcome data sources

Genetic associations with CAD were obtained from the CARDIoGRAMplusC4D (Coronary ARtery DIsease Genome wide Replication and Meta-analysis (CARDIoGRAM) plus The Coronary Artery Disease (C4D) Genetics) consortium and the FinnGen study R8 (Table 1) [22, 23]. The former study included approximately 185,000 participants, consisting of 60,801 individuals diagnosed as cases and 123,504 individuals serving as non-cases, who were from 48 independent cohort studies (www.cardiogramplusc4d.org). The CAD cases were defined as individuals presenting with acute coronary syndrome, chronic stable angina, coronary stenosis exceeding 50%, and myocardial infarction [22]. The FinnGen study represents one of the pioneering initiatives in the field of personalized medicine, with the primary objective of gathering and analyzing genomic and health information from a large cohort involving half a million Finnish participants (https://www.finngen.fi/en). For the current investigation, the R8 release of results of genome-wide association analysis was utilized, which encompassed 39,036 CAD cases (defined by the code 410|4110 in International Classification of Diseases (ICD)-Eighth Revision and the code 410|4110 in ICD-Ninth Revisions and the code I20.0, I21, or I22 in ICD-Tenth Revision) and 303,463 non-cases. Adjustments were made for age, sex, and up to top 20 genetic principal components.

### Statistical analysis

We harmonized the exposure and outcome data based on the effect and non-effect alleles for each SNP. SNPs with allele mismatch on effect or non-effect alleles were excluded from the analysis to ensure data integrity. Palindromic SNPs with minor allele frequency (MAF) below 0.42 were included in the analysis. Conversely, any palindromic SNPs flaunting a MAF between 0.42 and 0.5 were removed from the analysis. For SNPs unavailable in the outcome data, we searched their proxy SNPs in high linkage disequilibrium (*r*^2^ > 0.8) and replaced them with the proxy SNPs in the analysis. Missing SNPs without suitable proxies were removed from the analysis. To examine weak instrument bias, we have estimated the *F*-statistic to assess the strength of the used genetic instrumental variables. The SNP with the *F*-statistic < 10 was removed from the analysis. Used genetic instruments are presented in Additional file 2: Table S4. An online tool known as mRnd (https://shiny.cnsgenomics.com/mRnd/) was utilized for power calculation, and the statistical power exceeding 80% was deemed satisfying [24]. For the analysis with the exposure and outcome sample partially overlapped, we used an online tool (https://sb452.shinyapps.io/overlap/) to assess the bias from sample overlap and the corresponding type 1 error rate (Additional file 2: Table S5-S6) [25].

#### Proteome-wide MR and colocalization analysis

A proteome-wide MR analysis was conducted using lead *cis*-SNPs as genetic IVs for circulating proteins to investigate the associations between proteins and CAD risk [26]. The odds ratios (ORs) were determined using the Wald ratio method, while the corresponding confidence intervals (CIs) were estimated using the delta method. To account for multiple testing, the false discovery rate (FDR) was used by setting *α* = 0.05 under the Benjamini–Hochberg method. Colocalization analysis was performed to investigate whether the observed protein-CAD associations were influenced by linkage disequilibrium. For each genomic locus, the Bayesian method was employed to evaluate the evidence supporting five mutually exclusive hypotheses [27]. These hypotheses encompassed various scenarios regarding the association between the locus and two traits, including (1) absence of associations with either of the traits; (2) associations exclusively with one of the traits; (3) associations solely with the other trait; (4) associations with both traits, but involving distinct causal variants for each trait; and (5) associations with both traits, driven by shared causal variants. Posterior probabilities were derived for every hypothesis tested, denoted as H0, H1, H2, H3, and H4, while the prior probabilities of the variant being associated with one trait (*P*_1_) or the other trait (*P*_2_) only were set at 1 × 10^−4^, and the probability of association with both traits (*P*_3_) was set at 1 × 10^−5^. We considered two signals to have robust evidence of colocalization when the posterior probability for shared causal variants (*P*_H4_) was equal to or greater than 0.8. For cases where the *P*_H4_ fell between 0.5 and 0.8, it was considered as a moderate indication of colocalization. We additionally used *P*_H3_ data to evaluate confounding by linkage disequilibrium. In this case, strong evidence of colocalization analysis was defined by *P*_H3_ + *P*_H4_ > 0.8 and *P*_H4_/(*P*_H3_ + *P*_H4_) > 0.9, as previous [28, 29]. To increase power in colocalization analysis, we used the METAL software to meta-analyze the summary statistics derived from CARDIoGRAMplusC4D consortium and the FinnGen study [30]. The analysis was conducted using a fixed-effects model and the associations from two data sources were weighted by standard error of GWAS estimates.

#### Two-step network MR analysis

Two-sample MR analyses were performed to assess the associations of genetically predicted four obesity measures and 13 lifestyle factors with CAD risk and CAD-associated proteins’ levels. The analyses were conducted using the inverse-variance-weighted (IVW) method serving as the primary statistical model [31]. We reported the associations obtained from the fixed-effects IVW method if no significant heterogeneity was detected; otherwise, we reported the estimate from the multiplicative random-effects IVW method. The statistical significance threshold for the associations of genetically predicted modifiable factors with CAD risk was set at a two-sided *p* value of < 0.003 (= 0.05/19 tests). Subsequently, a two-step network MR analysis was performed to explore the potential mediation of identified CAD-associated proteins in the association between each pinpointed modifiable factor and CAD [19]. The indirect effect was calculated by multiplying the causal effect size of the modifiable factor on the CAD-associated protein by that of the protein-CAD association. The proportion mediated was further calculated by dividing the indirect effect by the total effect. The mediation analysis was confined to the modifiable factor-protein-CAD combination where the direction of the total effect (beta of the modifiable factor-CAD association) was in line with the direction of the effect through the mediator (beta of the modifiable factor-protein association × beta of the protein-CAD association).

To explore potential horizontal pleiotropy and validate the primary results, several supplementary analyses using different statistical models were conducted, namely the weighted-median method, MR-Egger regression, and MR Pleiotropy Residual Sum and Outlier (MR-PRESSO) methods [32–34]. The weighted median method is considered a reliable approach for estimating causal effects, particularly when < 50% of weight is derived from invalid genetic IVs [32]. The MR-Egger regression is a method to detect potential imbalance in pleiotropic effects across the genetic instruments utilizing an embedded intercept test, providing the corrected estimates adjusting for pleiotropy. Nevertheless, it may come at the expense of sacrificing statistical power [33]. The MR-PRESSO method provides the dual functionality of identifying potential outliers and deriving causal estimates that are relatively unbiased following the removal of such outliers [34]. Besides, it incorporates an intrinsic distortion test that allows the assessment of discrepancies between causal estimates prior to and following the removal of outliers [34]. Statistical analyses were performed using the METAL software and R software (version 4.2.0) with the utilization of packages named “TwoSampleMR (version 0.5.6),” “MendelianRandomization (version 0.7.0),” “MR-PRESSO (version 1.0),” and “coloc (version 5.2.3)” [30, 34–37].

## Results

**Fig. 2:**
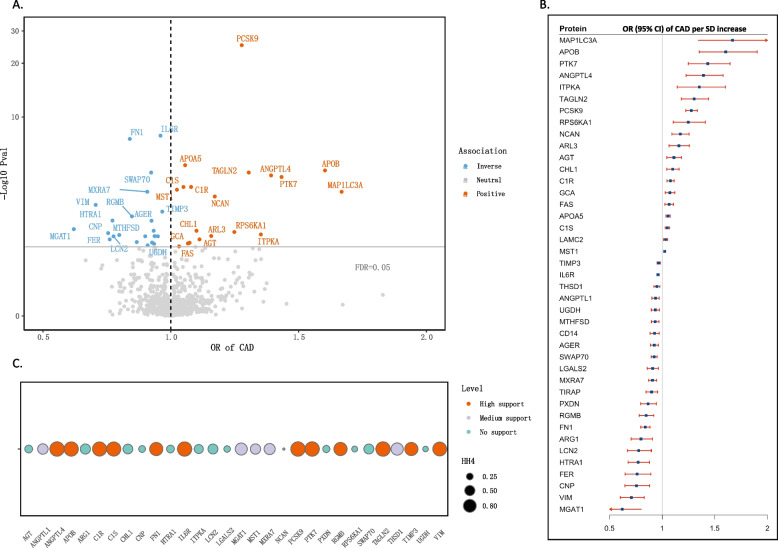
The associations of genetically predicted circulating proteins with CAD risk. **A** The volcano plot. The color of circle indicates the direction of association. **B** The forest plot. Odds ratios are scaled to per 1 standard deviation (SD) increase in the genetically predicted circulating proteins levels. **C** Colocalization analysis. Circle size indicates the colocalization *p* value for H4 and the color of circle indicates the classification of the evidence. Abbreviations: CAD, coronary artery disease; CI, confidence interval; OR, odds ratio

We investigated the associations between genetically predicted levels of 1247 circulating proteins, for which *cis*-index pQTL signals were available, and the risk of CAD (Fig. 2). A total of 165 protein-CAD associations were identified at the marginal significance level (*p* < 0.05) through the proteome-wide MR analysis. Following the application of Benjamini–Hochberg multiple testing correction, 41 circulating proteins had significant associations with CAD risk (FDR < 0.05; Fig. 2A, B). Out of those proteins, 19 showed positive associations with CAD, including MAP1LC3A (microtubule associated protein 1 light chain 3 alpha), APOB (apolipoprotein B), PTK7 (protein tyrosine kinase-7), among others (Fig. 2). For each 1-standard deviation (SD) increase in genetically predicted levels of protein, the ORs of CAD were 1.67 (95% CI, 1.34–2.07) for MAP1LC3A, 1.60 (95% CI, 1.35–1.90) for APOB, and 1.43 (95% CI, 1.25–1.65) for PTK7 (Fig. 2B), while 22 circulating proteins were inversely associated with CAD risk, such as MGAT1 (monoacylglycerol O-acyltransferase 1), VIM (vimentin), CNP (C-type natriuretic peptide), and others (Fig. 2). For 1-SD increment of genetically predicted protein levels, the ORs of CAD was 0.62 (95% CI, 0.48–0.80) for MGAT1, 0.71 (95% CI, 0.60–0.83) for VIM, and 0.75 (95% CI, 0.65–0.88) for CNP, respectively (Fig. 2B).

As a follow up analysis, colocalization analyses of the protein-CAD associations were performed (Fig. 2C). We identified strong evidence for 12 protein-CAD signals, including ANGPTL4 (angiopoietin-like 4), APOB, C1R, C1S, FN1 (fibronectin 1), IL6R (interleukin 6 receptor), PCSK9 (proprotein convertase subtilisin/kexin type 9), PTK7, RGMB (repulsive guidance molecule BMP co-receptor B), TAGLN2 (transgelin-2), TIMP3 (tissue inhibitor of metalloproteinases 3), and VIM. In addition, medium support evidence was observed for 5 circulating proteins, including ANGPTL1 (angiopoietin-like 1), MGAT1 (mannosyl (alpha-1,3-)-glycoprotein beta-1,2-N-acetylglucosaminyltransferase), MST1 (macrophage stimulating 1), MXRA7 (matrix remodeling-associated protein 7), and THSD1 (thrombospondin type-1 domain-containing protein 1). These signals remained robust when using both *P*_H3_ and *P*_H4_ to evaluate confounding by linkage disequilibrium (Additional file 2: Table S7).

**Fig. 3:**
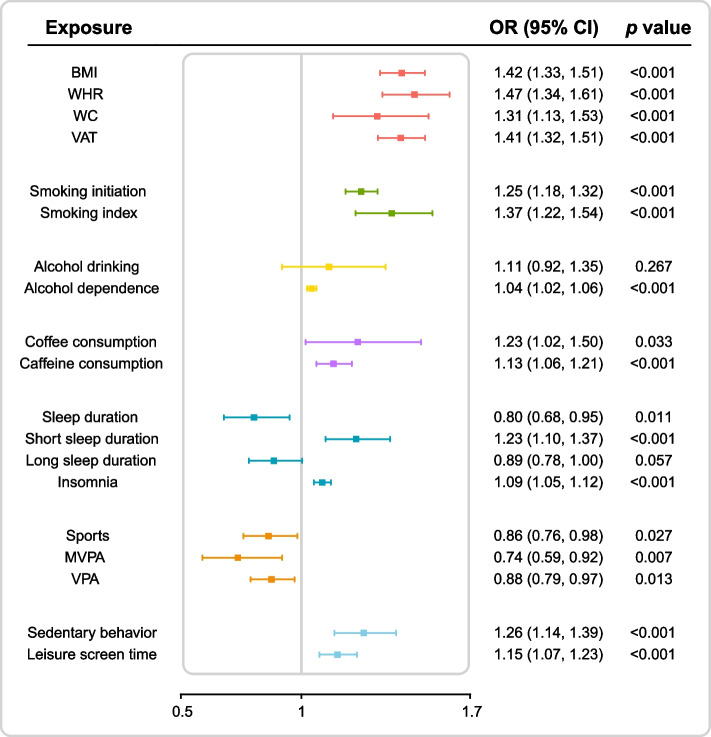
Two-sample Mendelian randomization analyses for the associations of genetically predicted obesity measures and lifestyle factors with the risk of coronary artery disease. Abbreviations: BMI, body mass index; CI, confidence interval; MVPA, moderate to vigorous physical activity; OR, odds ratio; VAT, visceral adipose tissue; VPA, vigorous physical activity; WC, waist circumference; WHR, waist-to-hip ratio

Subsequently, we conducted two-sample MR analyses to investigate the associations between genetically predicted four obesity measures and 13 lifestyle factors and CAD risk (Fig. 3). The IVW analyses revealed significant associations of genetically predicted obesity (as measured by BMI, WHR, WC, and VAT), smoking (both initiation and index), alcohol dependence, caffeine consumption, short sleep duration (< 7 h), insomnia, and sedentary behavior with the risk of CAD. Consistent and stable association patterns were observed in the sensitivity analyses employing various statistical methods (Additional file 2: Table S8-S12). Suggestive evidence emerged for the associations of genetically predicted coffee consumption, sleep duration, sports, moderate-to-vigorous physical activity, and vigorous physical activity with CAD risk after multiple testing correction. No consistent evidence was observed for the association of genetically predicted alcohol drinking and long sleep duration (> 9 h) with CAD.

**Fig. 4:**
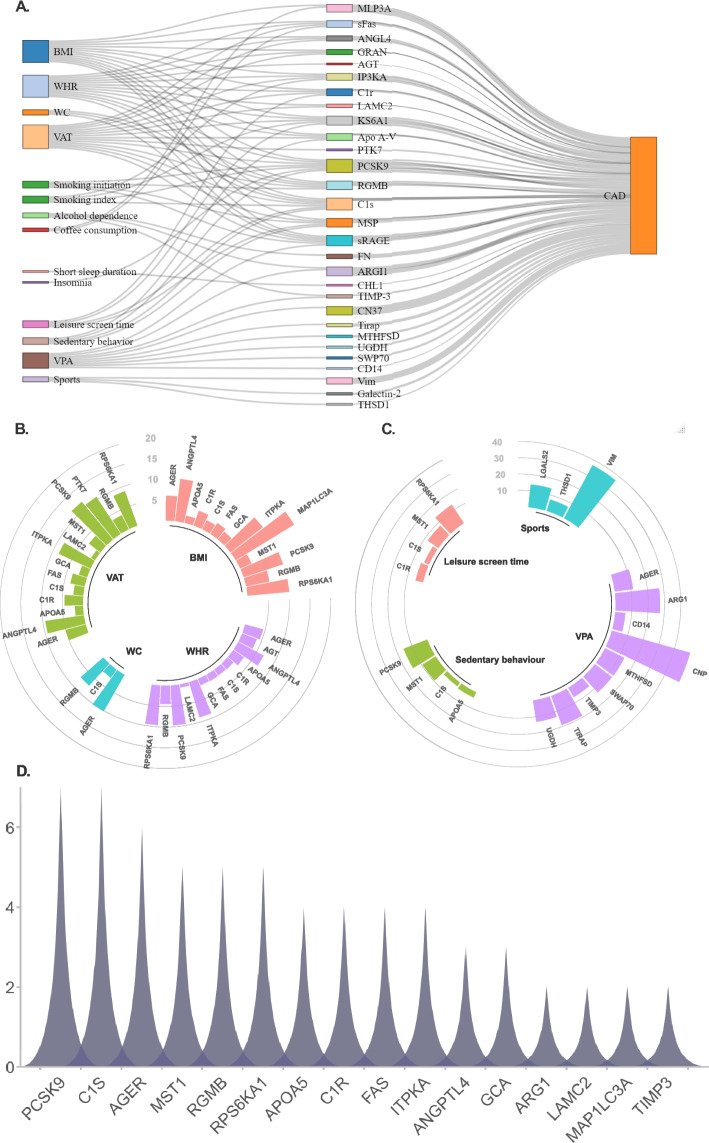
Two-step network mediation analysis connecting the obesity measures and lifestyle factors to coronary artery disease through the modulation of specific circulating proteins. **A** The overview of potential causal mediating network. **B** The proportion of the association between obesity and coronary artery disease mediated by circulating proteins. **C** The proportion of the association between of physical activity and sedentary behaviors with coronary artery disease mediated by circulating proteins. **D** The frequency of circulating proteins involved in the mediating network connecting modifiable factors to CAD risk. Abbreviations: BMI, body mass index; VAT, visceral adipose tissue; VPA, vigorous physical activity; WHR, waist-to-hip ratio; WC, waist circumference

We further examined the associations of the genetically predicted identified modifiable factors on the circulating levels of CAD-associated proteins (Additional file 2: Table S13-S21). A two-step network MR analysis was then conducted to explore the mediating network connecting the modifiable factors to CAD through the modulation of specific circulating proteins (Fig. 4A; Additional file 2: Table S22). We found that a significant proportion of the BMI-CAD association was mediated through MAP1LC3A (16.5% [95% CI 4.3–28.6%]). Additionally, we observed that ANGPTL4, RPS6KA1 (ribosomal protein S6 kinase A1), PCSK9, ITPKA (inositol-trisphosphate 3-kinase A), and AGER (advanced glycosylation end product-specific receptor) were each responsible for mediating 5 to 10% of the BMI-CAD association. Similarly, the positive association of WHR and VAT with CAD was mediated through PCSK9 (9.6% for WHR; 11.8% for VAT), RPS6KA1(9.6% for WHR; 8.8% for VAT), ITPKA (8.8% for WHR; 8.5% for VAT), ANGPTL4 (6.9% for WHR; 9.4% for VAT), and AGER (4.5% for WHR; 5.0% for VAT).

Furthermore, 36.8% (95% CI 16.5–57.2%) and 13.9% (95% CI 3.6–24.2%) of the inverse association between sports and CAD was mediated by VIM and LGALS2 (galectin-2), respectively, while the inverse association for vigorous physical activity was mainly mediated by CNP (49.9%), ARG1 (arginase-1; 27.2%) and AGER (11.3%). Regarding sedentary behaviors, 15.4% (95% CI 5.3–25.6%) and 9.4% (95% CI 1.2–17.6%) of the association with CAD were mediated by PCSK9 and MST1 (mammalian Ste20-like kinase 1), respectively. The association between leisure screen time and CAD was partly mediated by RPS6KA1 (13.4%) and MST1 (7.8%). Our analysis revealed that RGMB and PCSK9 mediated 9.0% (95% CI 2.8–15.2%) and 8.5% (95% CI 2.1–14.9%) of the association between smoking initiation and CAD. Additionally, ITPKA, PCSK9, and RPS6KA1 accounted for 11 to 13% of the positive association between smoking index and CAD. Besides, 33.5% (95% CI 20.5–46.6%), 9.9% (95% CI 4.7–15.2%), and 4.1% (95% CI 1.6–6.5%) of the association between caffeine consumption and CAD was mediated through APOB, VIM, and FAS (fatty acid synthase), respectively, while the association between coffee consumption and CAD was mainly mediated through MAP1LC3A (29.0%) and FAS (10.0%). Taken together, among these proteins, PCSK9, C1S, AGER, MST1, RGMB, and RPS6KA1 exhibited the highest frequency in the mediating network connecting modifiable factors to CAD risk (Fig. 4D). Most MR analyses had statistical power > 80%, except for some analyses of sports and coffee consumption (Additional file 2: Table S23).

## Discussion

In this study, we employed a comprehensive framework of proteome-wide MR and colocalization analyses to unravel the associations between a vast array of circulating proteins and CAD. The proteome-wide MR analyses identified 19 positive and 22 inverse protein-CAD associations. Among these associations, 12 and five protein-CAD associations had high and moderate colocalization support, respectively. Two-step network analysis indicated that many circulating proteins mediated the associations of genetically predicted obesity and some lifestyle factors with CAD. AGER and MST1, along with PCSK9 and C1S, exhibited the highest frequency among the identified causal mediating networks, which highlights their potential involvements in the pathogenesis and offers potential targets for CAD prevent and treatment.

We provided consistent evidence establishing causal links of 17 circulating proteins with CAD through a genetic framework. Our findings were partly in line with previous investigations. Family-based studies identified specific mutations in *APOB* and *PCSK9* as etiological factors contributing to development of familial hypercholesterolemia by either impeding the binding of LDL particles to the receptors, thus hindering the uptake, or by enhancing the catabolism of LDL receptors [3]. The strategy utilizing human monoclonal antibodies to selectively target ANGPTL4, thereby activating lipoprotein lipase, has demonstrated a substantial decrease in triglyceride-rich lipoproteins in both mice and monkeys [38]. Interleukin 6 (IL-6) signaling plays a crucial role in propagating downstream inflammation cascades, making it an attractive target for cardiovascular diseases [39]. However, the associations of plasma PTK7, RGMB, TAGLN2, TIMP3, and VIM with CAD have been scarcely investigated. PTK7, a transmembrane receptor with evolutionary conservation, plays a crucial role in embryonic development and tissue homeostasis [40]. RGMB represents a co-receptor for bone morphogenetic proteins, which was implicated in the angiogenesis and growth of colorectal cancer [41]. TIMP3 plays a crucial role in maintaining extracellular matrix balance. A recent MR study also demonstrated a causal association between elevated serum TIMP3 levels and a reduced risk of CAD [42].

Circulating proteins play a pivotal role in mediating the connection between modifiable factors and the susceptibility to CAD. The detrimental effect of obesity on CAD appears to be primarily mediated by MAP1LC3A, ANGPTL4, RPS6KA1, PCSK9, ITPKA, and AGER. A multinational prospective study involving 539 CAD patients indicated that plasma PCSK9 levels were associated with metabolic syndrome, insulin resistance, and obesity [43]. Another study involving thirty-four Japanese patients undergoing elective open-heart surgery reported a significant increase in the expression of ANGPTL4 in the epicardial adipose tissue of patients with CAD [44]. It was observed that the expression of MAP1LC3A was up-regulated in the adipose tissue of obese patients, indicating a close association with enhanced autophagy [45]. Experiments in mice suggested that RPS6K1 played an important role in the commitment of embryonic stem cells to early adipocyte progenitors [46]. Considering the limited evidence, potential mediating roles of these proteins deserve further investigations. Besides, physical activity seemed to increase circulating levels of VIM, LGALS2, CNP, ARG1, and AGER, thereby reducing the risk of CAD. In a 24-week study involving 61 Chinese patients, walking exercise resulted in a substantial increase in plasma CNP levels compared to a control group, highlighting its potential as an effective intervention [47]. ARG1 has been implicated in promoting anti-inflammatory (M2) phenotypes and was linked to stroke severity [48]. PCSK9, MST1, and RPS6K1 predominantly mediated the detrimental effect of sedentary behavior on CAD, while the effect of smoking was mainly mediated by RGMB, PCSK9, ITPKA, RPS6KA1, and AGER, which partially aligned with the observed association pattern between obesity and CAD. Our study revealed a potential detrimental effect of coffee and caffeine consumption on CAD, with mediation through APOB, VIM, FAS, and MAP1LC3A. Consistent with our findings, a recent MR study also suggested a causal association between coffee consumption and an increased CAD risk [49].

AGER and MST1 exhibited the highest frequency among the mediating networks, offering potential targets for CAD prevent and treatment, especially in individuals with obesity and unhealthy lifestyle factors. AGER, also known as RAGE, is expressed in various cell populations and its role extends to chronic inflammation and atherosclerosis [50, 51]. Upon ligand binding, RAGE triggers the production of proinflammatory cytokines and migration of leukocytes as well as tissue infiltration [50]. In animal studies, soluble RAGE (sRAGE) demonstrated atheroprotective properties [50]. In a population-based cohort study involving 4612 individuals, elevated levels of circulating sRAGE exhibited significant associations with a reduced rate of carotid intima-media thickness progression and a decreased risk of major coronary events [52]. Obesity may have the potential to intensify RAGE hyperactivation and trigger platelet activation, thereby contributing to the development of metabolic and vascular disorders [53]. In the Taipei Children Heart Study-III, a negative correlation was observed between sRAGE and BMI and blood lipid as well as glycemic [54]. In a prospective study, patients with chronic obstructive pulmonary disease exhibited significantly reduced serum sRAGE levels and smoking severely decreased sRAGE levels [55]. MST1 is a member of Ste-20 family and a key component of Hippo pathway, playing a pivotal role in diverse physiological processes [56]. Overexpression of MST1 in mice can lead to increased cardiomyocyte death, left ventricular fibrosis, and deterioration of cardiac function following myocardial infarction [57]. Another animal study also indicated that the knockdown of MST1 gene resulted in a notable reduction of atherosclerotic plaque [58]. However, the exact mechanism underlying the involvement of MST1 in atherosclerosis remains to be elucidated. The specific role of MST1 in relation to the modifiable factors has received limited investigation thus far. In mice fed a high-fat diet, MST1 knockout was reported to attenuate obesity-related non-alcoholic fatty liver disease by reversing mitophagy [59]. A recent whole genome sequencing study identified MST1 as one of potential lung cancer-associated gene mutations [60]. In mice with experimentally induced diabetes, physical exercise was found to suppress MST1 expression and ameliorate cardiac remodeling [61].

A notable strength of current study lies in its employment of the MR design, which mitigates bias arising from reverse causation by utilizing fixed genetic variants at conception and thus unaffected by disease status. Additionally, our study minimized the influence of confounding factors, as genetic variants associated with the exposure of interest are typically inherited independently of environmental exposures. We performed colocalization analysis, a robust method for uncovering the pleiotropic effects of specific loci on multiple traits. Moreover, we explored the potential mediators between various modifiable factors and CAD, shedding light on the mediating network that connects obesity and lifestyle factors, proteins and CAD, which contributed to a deepened understanding. Nevertheless, it is necessary to acknowledge certain limitations. Firstly, our MR analysis focused only on a subset of proteins with available *cis*-index pQTL signals. Secondly, the study cohort primarily consisted of individuals of European ancestry, which may restrict the generalizability of our findings to other populations. Thirdly, dietary factors, such as red and processed meat consumption, are also closely linked with CAD. However, the analysis for these factors cannot be performed due to lack of valid genetic instrumental variables. Fourthly, there were small sample overlaps between some modifiable factor GWAS datasets and CAD data sources, like waist circumference (4.9%), smoking initiation (1.8%), alcohol drinking (1.8%), and caffeine consumption (0.4%). However, we estimated that the biases potentially caused by the sample overlap were < 0.002 and corresponding type 1 error rates were ≤ 0.05, indicating that our results should be limitedly affected by this small sample overlap. Besides, given that the MR associations reflect a lifelong effect of genetically predicted the exposure on the outcome, our results should be interpreted with caution, especially when comparing our findings with the effects of a short-term lifestyle intervention. Finally, we unfortunately cannot examine the interactions between the exposures and mediating blood proteins given lack of individual-level data.

## Conclusions

In summary, the current study identified 19 positive and 22 inverse protein-CAD associations. These circulating proteins appeared to mediate many associations of obesity and lifestyle factors with CAD risk. Notably, AGER and MST1 exhibited high frequency among the identified mediating networks, which may underscore potential involvements in the pathogenesis and offer promising therapeutic targets to mitigate CAD risk in individuals with obesity and unhealthy lifestyle factors.

### Supplementary Information

**Additional file 1.** The STROBE-MR checklist.

**Additional file 2:** **Table S1.** [Adjustments in the GWAS]. **Table S2.** [Websites of the used GWAS datasets]. **Table S3.** [Detailed definitions of obesity measures and lifestyle factors]. **Table S4.** [The used genetic instrumental variables]. **Table S5.** [Sample overlap between used GWAS data sources]. **Table S6.** [The bias and type 1 error rate caused by the sample overlap]. **Table S7.** [Posterior probability of H3 and H4 in colocalization analysis]. **Table S8.** [Associations of obesity measures with CAD in sensitivity analyses]. **Table S9.** [Associations of smoking, alcohol use and coffee consumption with CAD in sensitivity analyses]. **Table S10.** [Associations of sleep traits with CAD in sensitivity analyses]. **Table S11.** [Associations of physical activity and sedentary behavior with CAD in sensitivity analyses]. **Table S12.** [Associations of obesity and lifestyle factors with CAD in MR-PRESSO analyses]. **Table S13.** [Associations of obesity measures with MAP1LC3A]. **Table S14.** [Associations of obesity measures with levels of ANGPTL4]. Table S15- [Associations of obesity measures with RPS6KA1]. **Table S16.** [Associations of obesity measures with PCSK9]. **Table S17.** [Associations of obesity measures with ITPKA]. **Table S18.** [Associations of physical activity with circulating proteins]. **Table S19.** [Associations of sedentary behavior with circulating proteins]. **Table S20.** [Associations of smoking with circulating proteins]. **Table S21.** [Associations of coffee and caffeine consumption with circulating proteins]. **Table S22.** [The estimates of the indirect effect and proportion mediated]. **Table S23.** [The statistical power of Mendelian randomization analyses].

## Data Availability

All the data used in the present study had been publicly available. The original contributions presented in the study are included in the article/supplementary material; further inquiries can be directed to the corresponding author/s.

## Abbreviations

CAD: Coronary artery disease
LDL: Low-density lipoprotein
MR: Mendelian randomization
IV: Instrumental variable
SNP: Single nucleotide polymorphism
GWAS: Genome-wide association study
pQTL: Protein quantitative trait loci
BMI: Body mass index
WC: Waist circumference
WHR: Waist-to-hip ratio
VAT: Visceral adipose tissue
CARDIoGRAMplusC4D: Coronary ARtery DIsease Genome wide Replication and Meta-analysis (CARDIoGRAM) plus The Coronary Artery Disease (C4D) Genetics consortium
ICD: International classification of diseases
MAF: Minor allele frequency
OR: Odds ratio
CI: Confidence interval
FDR: False discovery rate
IVW: Inverse-variance-weighted
MR-PRESSO: MR Pleiotropy Residual Sum and Outlier method
MAP1LC3A: Microtubule associated protein 1 light chain 3 alpha
APOB: Apolipoprotein B
PTK7: Protein tyrosine kinase-7
MGAT1: Monoacylglycerol O-acyltransferase 1
VIM: Vimentin
CNP: C-type natriuretic peptide
ANGPTL4: Angiopoietin-like 4
FN1: Fibronectin 1
IL6R: Interleukin 6 receptor
PCSK9: Proprotein convertase subtilisin/kexin type 9
RGMB: Repulsive guidance molecule BMP co-receptor B
TAGLN2: Transgelin-2
TIMP3: Tissue inhibitor of metalloproteinases 3
ANGPTL1: Angiopoietin-like 1
MGAT1: Mannosyl (alpha-1,3-)-glycoprotein beta-1,2-N acetylglucosaminyltransferase
MST1: Macrophage stimulating 1
MXRA7: Matrix remodeling-associated protein
THSD1: Thrombospondin type-1 domain-containing protein 1
RPS6KA1: Ribosomal protein S6 kinase A1
ITPKA: Inositol-trisphosphate 3-kinase A
AGER: Advanced glycosylation end product-specific receptor
ARG1: Arginase-1
