# Supplementary material for: Proteomic insights into the associations between obesity, lifestyle factors, and coronary artery disease
Source: BMC Med. 2023 Dec 5;21:485. doi: 10.1186/s12916-023-03197-8 (PMC10696760; doi:10.1186/s12916-023-03197-8)
Supplement: Supplementary file 1 — Additional file 1. The STROBE-MR checklist. [file 12916_2023_3197_MOESM1_ESM.pdf]

STROBE-MR checklist of recommended items to address in reports of Mendelian randomization studies[1 2]

Item No.	Section		Checklist item	Page No.	Relevant text from manuscript
1	**TITLE and ABSTRACT**		Indicate Mendelian randomization (MR) as the study's design in the title and/or the abstract if that is a main purpose of the study	3	Two-sample MR analyses were performed to assess the causal effects of 19 lifestyle factors on CAD risk and the circulating concentrations of CAD-associated proteins.
	**INTRODUCTION**				
2	**Background**		Explain the scientific background and rationale for the reported study. What is the exposure? Is a potential causal relationship between exposure and outcome plausible? Justify why MR is a helpful method to address the study question	5-6	The application of Mendelian randomization (MR), specifically leveraging genetic variants predicting the concentration of circulating proteins, could strengthen potential for causal inference. Therefore, we conducted the current study to explore the causal effects of circulating proteins on CAD and thus to investigate potential therapeutic targets by employing a proteome-wide MR analysis.
3	**Objectives**		State specific objectives clearly, including pre-specified causal hypotheses (if any). State that MR is a method that, under specific assumptions, intends to estimate causal effects	6	Here, we conducted a study to identify blood proteins associated with CAD by employing a proteome-wide MR approach and further explored the mediating network involving modifiable factors, proteins, and CAD, thereby contributing to a deeper understanding of the pathogenesis.
	**METHODS**				
4	**Study design and data sources**		Present key elements of the study design early in the article. Consider including a table listing sources of data for all phases of the study. For each data source contributing to the analysis, describe the following:		
		a)	Setting: Describe the study design and the underlying population, if possible. Describe the setting, locations, and relevant dates, including periods of recruitment, exposure, follow-up, and data collection, when available.	6-7	A comprehensive investigation employing an integrated genetic approach was designed (Figure 1).
		b)	Participants: Give the eligibility criteria, and the sources and methods of selection of participants. Report the sample size, and whether any power or sample size calculations were carried out prior to the main analysis	7-9	We obtained summary-level statistics from a comprehensive protein quantitative trait loci (pQTL) study conducted in a population of 35,559 individuals of Icelandic descent. Additionally, the online tool known as mRnd (https://shiny.cnsgenomics.com/mRnd/) was utilized for the power calculation, and the statistical power exceeding 80% was deemed satisfying.

		c)	Describe measurement, quality control and selection of genetic variants	9	We harmonized the exposure and outcome data based on the effect and non-effect alleles for each SNP. SNPs with allele mismatch on effect or non-effect alleles were excluded from the analysis to ensure data integrity. Palindromic SNPs with minor allele frequency (MAF) below the 0.42 were included in the analysis. Conversely, any palindromic SNPs flaunting a MAF between 0.42 and 0.5 were removed from the analysis. For SNPs unavailable in the outcome data, we searched their proxy SNPs in high linkage disequilibrium (r2 > 0.8) and replaced them with the proxy SNPs in the analysis. Missing SNPs without suitable proxies were removed from the analysis. To examine weak instrument bias, we have estimated the F-statistic to assess the strength of the used genetic instrumental variables. The SNP with the F-statistic < 10 was removed from the analysis.
		d)	For each exposure, outcome, and other relevant variables, describe methods of assessment and diagnostic criteria for diseases	8	The data sources of used GWAS datasets and detailed definitions of modifiable lifestyle factors were provided in the Supplementary Table 1-3.
		e)	Provide details of ethics committee approval and participant informed consent, if relevant	6	The included genome-wide association studies (GWASs) had obtained the necessary ethical approvals from the relevant committees and written informed consent was obtained from all individuals involved in these studies
5	**Assumptions**		Explicitly state the three core IV assumptions for the main analysis (relevance, independence and exclusion restriction) as well assumptions for any additional or sensitivity analysis	6-7	In detail, the validity of two-step MR results also relies on three fundamental assumptions: (i) relevance assumption, i.e., the genetic variants should exhibit a strong association with the exposure, (ii) independence assumption, i.e., the genetic variants should be independent of potential confounding variables; and (iii) exclusion restriction, i.e., the genetic variants should solely impact the outcome through the exposure.
6	**Statistical methods: main analysis**		Describe statistical methods and statistics used		
		a)	Describe how quantitative variables were handled in the analyses (i.e., scale, units, model)	7	The aptamers further underwent adjustment for age and sex by applying an adjusted rank-inverse normal transformation to their levels. Subsequently, the residuals were also subjected to standardization using rank-inverse normal

					transformation and served as phenotypes in the genome-wide association analyses.
		b)	Describe how genetic variants were handled in the analyses and, if applicable, how their weights were selected	8	Genetic IVs for these factors were constructed by selecting single nucleotide polymorphisms (SNPs) identified at the genome-wide significance threshold ($p < 5×10−8$) and in low linkage disequilibrium ($r2 < 0.01$). The data sources of used GWAS datasets and detailed definitions of modifiable factors were provided in the Supplementary Table 1-3.
		c)	Describe the MR estimator (e.g. two-stage least squares, Wald ratio) and related statistics. Detail the included covariates and, in case of two-sample MR, whether the same covariate set was used for adjustment in the two samples	10-11	Two-sample MR analyses were performed to assess the causal effects of 19 lifestyle factors on CAD risk and the circulating concentrations of CAD-associated proteins, with inverse-variance-weighted (IVW) method serving as the primary statistical model.25 The statistical significance threshold for the link of lifestyle factors with CAD risk was set at a two-sided p value of <0.003 (=0.05/19 tests). Subsequently, a two-step network MR analysis was performed to explore the potential mediation of specific proteins in the relationship between each lifestyle factor and CAD.
		d)	Explain how missing data were addressed	9	For SNPs unavailable in the outcome data, we searched their proxy SNPs in high linkage disequilibrium ($r2 > 0.8$) and replaced them with the proxy SNPs in the analysis. Missing SNPs without suitable proxies were removed from the analysis.
		e)	If applicable, indicate how multiple testing was addressed	11	The statistical significance threshold for the link of lifestyle factors with CAD risk was set at a two-sided p value of <0.003 (=0.05/19 tests).
7	**Assessment of assumptions**		Describe any methods or prior knowledge used to assess the assumptions or justify their validity	7	To satisfy the first assumption, our IV selection was confined to SNPs achieving the genome-wide significance threshold. The second assumption is usually satisfied and a merit of the MR approach since genetic variants are randomly assorted at conception and therefore unassociated with confounders (e.g., environmental and self-adopted factors). The most challenge for MR analysis is the third assumption. For protein-wide MR analysis, this assumption is likely to be satisfied since we selected cis-SNPs with limited pleiotropic effects as IVs.

8	**Sensitivity analyses and additional analyses**	Describe any sensitivity analyses or additional analyses performed (e.g. comparison of effect estimates from different approaches, independent replication, bias analytic techniques, validation of instruments, simulations)	11	Furthermore, to explore potential horizontal pleiotropy and validate the primary results, several supplementary analyses using other different statistical models were conducted, namely the weighted-median method, MR-Egger regression, weighted mode method and MR Pleiotropy Residual Sum and Outlier (MR-PRESSO) methods.
9	**Software and pre-registration**			
	a)	Name statistical software and package(s), including version and settings used	11	Statistical analyses were performed using METAL software and R software (version 4.2.0) with the utilization of several packages, including 'TwoSampleMR', 'MendelianRandomization', 'MR-PRESSO' and 'coloc'.
	b)	State whether the study protocol and details were pre-registered (as well as when and where)		NA
	**RESULTS**			
10	**Descriptive data**			
	a)	Report the numbers of individuals at each stage of included studies and reasons for exclusion. Consider use of a flow diagram	9	For the current investigation, the R8 release of results of genome-wide association analysis was utilized, which encompassed a total of 39,036 cases of CAD (defined by the code 410|4110 in International Classification of Diseases (ICD)-Eighth Revision and the code 410|4110 in ICD-Ninth Revisions and the code I20.0, I21 or I22 in ICD-Tenth Revision) and 303,463 controls.
	b)	Report summary statistics for phenotypic exposure(s), outcome(s), and other relevant variables (e.g. means, SDs, proportions)	9	The data sources of used GWAS datasets and detailed definitions of modifiable lifestyle factors were provided in the Supplementary Table 1-3.
	c)	If the data sources include meta-analyses of previous studies, provide the assessments of heterogeneity across these studies	10	To increase power in colocalization analysis, we used the METAL software to meta-analyze the summary statistics derived from CARDIoGRAMplusC4D consortium and the FinnGen study.[29] The analysis was conducted using a fixed-effects model and the associations from two data sources were weighted by standard error of GWAS estimates.
	d)	For two-sample MR:	9	For the analysis with the exposure and outcome sample partially overlapped, we used an online tool

			i. Provide justification of the similarity of the genetic variant-exposure associations between the exposure and outcome samples<br>ii. Provide information on the number of individuals who overlap between the exposure and outcome studies		(https://sb452.shinyapps.io/overlap/) to assess the bias from sample overlap and the corresponding type 1 error rate (Supplementary Table 4-5).[24]
11	**Main results**				
		a)	Report the associations between genetic variant and exposure, and between genetic variant and outcome, preferably on an interpretable scale	12	For each 1-standard deviation (SD) increase in genetically predicted levels of protein, the ORs of CAD was 1.67 (95% CI, 1.34-2.07) for MAP1LC3A, 1.60 (95% CI, 1.35-1.90) for APOB, and 1.43 (95% CI, 1.25-1.65) for PTK7 (Figure 2B).
		b)	Report MR estimates of the relationship between exposure and outcome, and the measures of uncertainty from the MR analysis, on an interpretable scale, such as odds ratio or relative risk per SD difference	12	For each 1-standard deviation (SD) increase in genetically predicted levels of protein, the ORs of CAD was 1.67 (95% CI, 1.34-2.07) for MAP1LC3A, 1.60 (95% CI, 1.35-1.90) for APOB, and 1.43 (95% CI, 1.25-1.65) for PTK7 (Figure 2B).
		c)	If relevant, consider translating estimates of relative risk into absolute risk for a meaningful time period		NA
		d)	Consider plots to visualize results (e.g. forest plot, scatterplot of associations between genetic variants and outcome versus between genetic variants and exposure)	/	Figure 2
12	**Assessment of assumptions**				
		a)	Report the assessment of the validity of the assumptions	7	For MR analysis on modifiable risk factors, we conducted several supplementary analyses to fortify the resilience of the primary results as well as to detect and correct for potential horizontal pleiotropy. To examine potential bias due to weak instrument, we have estimated F-statistic to assess the strength of the used genetic instrumental variables. The SNP with the F-statistic < 10 was removed from the analysis. Additionally, the online tool known as mRnd (https://shiny.cnsgenomics.com/mRnd/) was utilized for the power calculation, and the statistical power exceeding 80% was deemed satisfying.
		b)	Report any additional statistics (e.g., assessments of heterogeneity across genetic variants, such as I^2, Q statistic or E-value)	7	For MR analysis on modifiable risk factors, we conducted several supplementary analyses to fortify the resilience of the primary results as well as to detect and correct for potential horizontal pleiotropy.

13	**Sensitivity analyses and additional analyses**				
		a)	Report any sensitivity analyses to assess the robustness of the main results to violations of the assumptions	13	Consistent and stable association patterns were observed in the sensitivity analyses employing various statistical methods (Supplementary Table 7-11).
		b)	Report results from other sensitivity analyses or additional analyses	13	Consistent and stable association patterns were observed in the sensitivity analyses employing various statistical methods (Supplementary Table 7-11).
		c)	Report any assessment of direction of causal relationship (e.g., bidirectional MR)		NA
		d)	When relevant, report and compare with estimates from non-MR analyses		NA
		e)	Consider additional plots to visualize results (e.g., leave-one-out analyses)	/	Figure 3-4
	**DISCUSSION**				
14	**Key results**		Summarize key results with reference to study objectives	15	The proteome-wide MR analyses identified a total of 19 positive and 22 inverse protein-CAD associations, among which 12 and 5 protein-CAD associations had high and moderate colocalization support, respectively. Two-step network analysis indicated causal mediation of many circulating proteins in the association between lifestyle factors and CAD. AGER and MST1, along with PCSK9 and C1S, exhibited the highest frequency among the identified causal mediating networks, which highlights their potential involvements in the pathogenesis and offers potential targets for CAD prevent and treatment.
15	**Limitations**		Discuss limitations of the study, taking into account the validity of the IV assumptions, other sources of potential bias, and imprecision. Discuss both direction and magnitude of any potential bias and any efforts to address them	18	Nevertheless, it is necessary to acknowledge certain limitations. Firstly…
16	**Interpretation**				
		a)	Meaning: Give a cautious overall interpretation of results in the context of their limitations and in comparison with other studies	18	Besides, given that the MR associations reflect a lifelong effect of genetically predicted exposure on the outcome, our results should be interpreted with caution, especially when comparing our findings with the effects of a short-term lifestyle intervention.

		b)	Mechanism: Discuss underlying biological mechanisms that could drive a potential causal relationship between the investigated exposure and the outcome, and whether the gene-environment equivalence assumption is reasonable. Use causal language carefully, clarifying that IV estimates may provide causal effects only under certain assumptions	17	Upon ligand binding, RAGE triggers the production of proinflammatory cytokines, migration of leukocytes, as well as tissue infiltration.44 In animal studies, soluble RAGE (sRAGE) demonstrated atheroprotective properties.
		c)	Clinical relevance: Discuss whether the results have clinical or public policy relevance, and to what extent they inform effect sizes of possible interventions	16	AGER and MST1 exhibited the highest frequency among the mediating networks, offering potential targets for CAD prevent and treatment, especially in individuals with unhealthy lifestyles.
17	**Generalizability**		Discuss the generalizability of the study results (a) to other populations, (b) across other exposure periods/timings, and (c) across other levels of exposure	18	Secondly, the study cohort primarily consisted of individuals of European ancestry, which may restrict the generalizability of our findings to other populations.
	**OTHER INFORMATION**				
18	**Funding**		Describe sources of funding and the role of funders in the present study and, if applicable, sources of funding for the databases and original study or studies on which the present study is based	20	This work was supported by grants from the Key Laboratory of Precision Medicine for Atherosclerotic Diseases of Zhejiang Province, China (Grant No. 2022E10026), National Natural Science Foundation of China (82200489), the Major Project of Science and Technology Innovation 2025 in Ningbo, China (Grant No. 2021Z134), the Key research and development project of Zhejiang Province, China (Grant No. 2021C03096).
19	**Data and data sharing**		Provide the data used to perform all analyses or report where and how the data can be accessed, and reference these sources in the article. Provide the statistical code needed to reproduce the results in the article, or report whether the code is publicly accessible and if so, where	19	All the data used in the present study had been publicly available. The original contributions presented in the study are included in the article/Supplementary Material, further inquiries can be directed to the corresponding author/s.
20	**Conflicts of Interest**		All authors should declare all potential conflicts of interest	19	The authors declare that they have no competing interests.

This checklist is copyrighted by the Equator Network under the Creative Commons Attribution 3.0 Unported (CC BY 3.0) license.

1. Skrivankova VW, Richmond RC, Woolf BAR, Yarmolinsky J, Davies NM, Swanson SA, et al. Strengthening the Reporting of Observational Studies in Epidemiology using Mendelian Randomization (STROBE-MR) Statement. JAMA. 2021;under review.

2. Skrivankova VW, Richmond RC, Woolf BAR, Davies NM, Swanson SA, VanderWeele TJ, et al. Strengthening the Reporting of Observational Studies in Epidemiology using Mendelian Randomisation (STROBE-MR): Explanation and Elaboration. BMJ. 2021;375:n2233.
