# Supplementary material for: Proteomic insights into the associations between obesity, lifestyle factors, and coronary artery disease
Source: BMC Med. 2023 Dec 5;21:485. doi: 10.1186/s12916-023-03197-8 (PMC10696760; doi:10.1186/s12916-023-03197-8)
Supplement: Supplementary file 2 — Additional file 2: Table S1. [Adjustments in the GWAS]. Table S2. [Websites of the used GWAS datasets]. Table S3. [Detailed definitions of obesity measures and lifestyle factors]. Table S4. [The used genetic instrumental variables]. Table S5. [Sample overlap between used GWAS data sources]. Table S6. [The bias and type 1 error rate caused by the sample overlap]. Table S7. [Posterior probability of H3 and H4 in colocalization analysis]. Table S8. [Associations of obesity measures with CAD in sensitivity analyses]. Table S9. [Associations of smoking, alcohol use and coffee consumption with CAD in sensitivity analyses]. Table S10. [Associations of sleep traits with CAD in sensitivity analyses]. Table S11. [Associations of physical activity and sedentary behavior with CAD in sensitivity analyses]. Table S12. [Associations of obesity and lifestyle factors with CAD in MR-PRESSO analyses]. Table S13. [Associations of obesity measures with MAP1LC3A]. Table S14. [Associations of obesity measures with levels of ANGPTL4]. Table S15- [Associations of obesity measures with RPS6KA1]. Table S16. [Associations of obesity measures with PCSK9]. Table S17. [Associations of obesity measures with ITPKA]. Table S18. [Associations of physical activity with circulating proteins]. Table S19. [Associations of sedentary behavior with circulating proteins]. Table S20. [Associations of smoking with circulating proteins]. Table S21. [Associations of coffee and caffeine consumption with circulating proteins]. Table S22. [The estimates of the indirect effect and proportion mediated]. Table S23. [The statistical power of Mendelian randomization analyses]. [file 12916_2023_3197_MOESM2_ESM.pdf]

Supplementary materials for

Proteomic insights into the associations between obesity, lifestyle factors, and coronary artery disease

Table of Contents

Supplemental Table 1. Adjustments in the GWAS on obesity and lifestyle factors. 2

Supplemental Table 2. Websites of the used GWAS datasets. 3

Supplemental Table 3. Detailed definitions of obesity measures and lifestyle factors. 4

Supplementary Table 4. The used genetic instrumental variables for obesity measures and lifestyle factors in Mendelian randomization analysis. 6

Supplemental Table 5. Sample overlap between used GWAS data sources. 79

Supplemental Table 6. The bias and type 1 error rate caused by the sample overlap. 80

Supplemental Table 7. Posterior probability of H3 and H4 in colocalization analysis. 81

Supplemental Table 8. Associations of genetically predicted obesity measures with CAD in sensitivity analyses. 82

Supplemental Table 9. Associations of genetically predicted smoking, alcohol use and coffee consumption with CAD in sensitivity analyses. 83

Supplemental Table 10. Associations of genetically predicted sleep traits with CAD in sensitivity analyses. 84

Supplemental Table 11. Associations of genetically predicted physical activity and sedentary behavior with CAD in sensitivity analyses. 85

Supplemental Table 12. Associations of genetically predicted obesity and lifestyle factors with CAD in MR-PRESSO Analyses. 86

Supplemental Table 13. Associations of genetically predicted obesity measures with levels of MAP1LC3A. 87

Supplemental Table 14. Associations of genetically predicted obesity measures with levels of ANGPTL4. 88

Supplemental Table 15. Associations of genetically predicted obesity measures with levels of RPS6KA1. 89

Supplemental Table 16. Associations of genetically predicted obesity measures with levels of PCSK9. 90

Supplemental Table 17. Associations of genetically predicted obesity measures with levels of ITPKA. 91

Supplemental Table 18. Associations of genetically predicted physical activity with levels of circulating proteins. 92

Supplemental Table 19. Associations of genetically predicted sedentary behavior with levels of circulating proteins. 93

Supplemental Table 20. Associations of genetically predicted smoking with levels of circulating proteins. 94

Supplemental Table 21. Associations of genetically predicted coffee and caffeine consumption with levels of circulating proteins. 95

Supplemental Table 22. The estimates of the indirect effect and proportion mediated. 96

Supplemental Table 23. The statistical power of Mendelian randomization analyses. 100

Supplemental Table 1. Adjustments in the GWAS on obesity and lifestyle factors.

Trait	Adjustments	PMID
Body mass index	Age, sex, and genetic 1–5 principal components	30239722
Waist-to-hip ratio	Age, sex, and genetic 1–5 principal components	30239722
Waist circumference	Age and study-specific covariates	25673412
Visceral adipose tissue	Age, sex, batch and the first ten principal components	31501611
Smoking initiation	Age, sex, and the first ten genetic principal components	30643251
Lifetime smoking index	Genotyping chip and sex	31689377
Alcohol drinking	Age, sex, and the first ten genetic principal components	30643251
Alcohol dependence	Sex and 1-5 principal components	30482948
Coffee consumption	Age, sex, body mass index, total energy, proportion of typical food intake, and 20 genetic principal components	31046077
Caffeine consumption	Age, sex, study-site, fasting status, smoking status, and genetic principal components	27702941
Moderate-to-vigorous physical activity	Age, sex, age-squared, principal components reflecting population structure	36071172
Vigorous physical activity	Age, sex, genotyping chip, first ten genomic principal components, and center	29899525
Sedentary behavior	Age-squared, age, sex, age-sex interaction, and the first 30 principal components	32317632
Leisure screen time	Age, sex, age-squared, principal components reflecting population structure	36071172
Sleep duration	Age, sex, and 10 principal components	30846698
Insomnia	Age, sex, genotype array, and 10 genetic principal components in the UK Biobank; age, sex and the top five principal components in 23andMe	30804565

Supplemental Table 2. Websites of the used GWAS datasets.

Trait	Website
Circulating protein	https://www.decode.com/summarydata/
CAD (Consortia)	http://www.cardiogramplusc4d.org/
CAD (FinnGen)	https://www.finngen.fi/
Body mass index	https://zenodo.org/records/1251813
Waist-to-hip ratio	https://zenodo.org/records/1251813
Visceral adipose tissue	https://www.ebi.ac.uk/gwas/publications/31501611
Waist circumference	https://portals.broadinstitute.org/collaboration/giant/index.php/GIANT_consortium_data_files#GWAS_Anthropometric_2015_Waist_Summary_Statistics
Smoking initiation	https://genome.psych.umn.edu/index.php/GSCAN
Lifetime smoking index	https://data.bris.ac.uk/data/dataset/10i96zb8gm0j81yz0q6ztei23d
Alcohol drinking	https://genome.psych.umn.edu/index.php/GSCAN
Alcohol dependence	http://www.med.unc.edu/pgc/results-and-downloads
Coffee consumption	https://digitalhub.northwestern.edu/users/mcc340
Caffeine consumption	https://digitalhub.northwestern.edu/users/mcc340
Sports	https://www.ukbiobank.ac.uk/
MVPA	https://www.ebi.ac.uk/gwas/downloads/summary-statistics
VPA	https://www.ukbiobank.ac.uk/
Sedentary behavior	https://data.mendeley.com/datasets/mxjj6czsrd/1
Leisure screen time	https://www.ebi.ac.uk/gwas/downloads/summary-statistics
Sleep duration	http://sleepdisordergenetics.org/
Insomnia	https://ctg.cncr.nl/software/summary_statistics

Note: CAD, coronary artery disease; MVPA, moderate to vigorous physical activity; VPA, vigorous physical activity.

Supplemental Table 3. Detailed definitions of obesity measures and lifestyle factors.

Trait	Definition
Body mass index	The body mass divided by the square of the body height
Waist-to-hip ratio	The measured circumference of the waist divided by the measured circumference of the hip.
Visceral adipose tissue	The mass of visceral adipose tissue measured by Dual-energy X-ray absorptiometry
Waist circumference	Obtained by measuring the abdomen at the level of the superior iliac crest with a tape measure
Smoking initiation	This is a binary phenotype. Code "2" for everyone in the study who reports ever being a regular smoker in their life (current or former). Code "1" for everyone who denies ever being a regular smoker in their life.
Lifetime smoking index	The smoking measures were combined into a lifetime smoking index along with a simulated half-life (τ) constant. Half-life captures the exponentially decreasing effect of smoking at a given time on health outcomes. The value of half-life was determined by simulating the effects of lifetime smoking on lung cancer and overall mortality in the UK Biobank.
Alcohol drinking	The average number of drinks a subject reports drinking each week, aggregated across all types of alcohol
Alcohol dependence	Defined as meeting criteria for a DSM-IV2 diagnosis of alcohol dependence
Coffee consumption	Total coffee consumption was based on the question 'How many cups of coffee do you drink each day (include decaffeinated coffee)?'
Caffeine consumption	Measured by plasma caffeine-related metabolites (137X, 17X, 13X, 37X and 137U)
Sports	Defined as spending 2–3 days/week or more doing strenuous sports or other exercises for a duration of 15–30 min or greater
MVPA	MVPA= '1' if subjects report engaging in ≥1 h/wk of leisure moderate/vigorous activity. These include activities with MET values of 3 or greater. Otherwise, MVPA= '0'."
VPA	Participants were asked: "In a typical week, how many days did you do 10 min or more of vigorous physical activity? (Activities making you sweat or breathe hard such as fast cycling, aerobics, heavy lifting)". Those who indicated 1 or more such days were then asked "How many minutes did you usually spend doing vigorous activities on a typical day".
Sedentary behavior	Participants were asked three questions, "In a typical DAY, how many hours do you spend watching TV?", "In a typical DAY, how many hours do you spend using the computer?" and "In a typical DAY, how many hours do you spend driving?"
Leisure screen time	The time spent watching TV as a quantitative trait, expressed in hours/day.
Sleep duration	Participants were asked: About how many hours sleep do you get in every 24 h? (including naps), with responses in hour increments. Sleep duration was treated as a continuous variable
Insomnia	Insomnia complaints were assessed by asking: "Do you have trouble falling asleep at night or do you wake up in the middle of the night?" with following four answers: "never/rarely", "sometimes", "usually", or "prefer not to answer". Insomnia cases were defined as participants who answered this question with "usually"

Note: For the construction of the lifetime smoking index, several smoking measures were combined into a lifetime smoking index along with a simulated half-life (τ) constant. The simulated half-life (τ) constant captures the exponentially decreasing effect of smoking at a given time on health outcomes. These values were used to fit the final model which is:

$tsc^* = \max(tsc - \delta, 0)$
$dur^* = \max(dur + tsc - \delta, 0) – tsc^*$
$\text{lifetime smoking} = (1 – 0.5^{dur^*/\tau})\ (0.5^{tsc^*/\tau})\ \ln(int+1)$

…where τ = half-life, δ = lag time, int = cigarettes per day, tss = time started smoking, tsc = time since cessation, dur = duration of smoking (either age-tss for current smokers or [age-tsc]-tss for former smokers). So, where δ = 0, tsc* = tsc and consequently, the lifetime smoking calculation can be simplified to:

$\text{Lifetime smoking index} = (1 – 0.5^{dur/\tau})\ (0.5^{tsc/\tau})\ \ln(int+1).$

Supplementary Table 4. The used genetic instrumental variables for obesity measures and lifestyle factors in Mendelian randomization analysis.

Phenotype	SNP	CHR:POS	EA	Beta	SE	EAF	*P*	*F*-statistic
BMI	rs10044136	5:153546808	G	0.0162	0.0017	0.43	3.1E-21	104
BMI	rs10099330	8:143383694	G	0.0119	0.0017	0.45	3.2E-12	57
BMI	rs10116186	9:80532374	G	0.0116	0.0019	0.56	1.6E-09	53
BMI	rs10132280	14:25928179	C	0.0214	0.0018	0.68	2.3E-33	162
BMI	rs10203386	2:25136866	A	0.0318	0.0017	0.41	1.4E-78	396
BMI	rs1030015	7:78139581	T	0.0109	0.0017	0.51	1.2E-10	48
BMI	rs1045411	13:31033232	C	0.0139	0.0019	0.69	6.7E-14	66
BMI	rs1048303	7:100804140	C	0.0110	0.0017	0.38	1.5E-10	46
BMI	rs10497870	2:203970283	A	0.0121	0.0016	0.53	2.0E-13	59
BMI	rs10499694	7:50614173	A	0.0130	0.0016	0.55	1.3E-15	67
BMI	rs10510419	3:12426936	G	0.0168	0.0023	0.86	2.2E-13	56
BMI	rs10518269	19:31028666	C	0.0173	0.0023	0.85	4.7E-14	61
BMI	rs10744146	12:17212881	G	0.0113	0.0017	0.48	2.5E-11	51
BMI	rs10761247	9:96403367	G	0.0113	0.0017	0.45	4.5E-11	51
BMI	rs10761785	10:65318766	G	0.0133	0.0016	0.48	3.5E-16	71
BMI	rs10823893	10:53677313	A	0.0124	0.0019	0.40	1.6E-10	60
BMI	rs10858334	9:137989785	G	0.0148	0.0025	0.11	4.9E-09	33
BMI	rs10887584	10:88112323	A	0.0126	0.0019	0.43	4.6E-11	63
BMI	rs10920678	1:190239907	A	0.0149	0.0016	0.49	7.2E-20	89
BMI	rs10929925	2:6155557	C	0.0142	0.0016	0.52	3.0E-18	81
BMI	rs10938397	4:45182527	G	0.0322	0.0016	0.45	2.4E-86	414
BMI	rs10942267	5:80841914	A	0.0148	0.0018	0.75	6.5E-16	67
BMI	rs10954772	8:30863938	T	0.0155	0.0021	0.31	5.3E-14	83
BMI	rs10984756	9:122651784	G	0.0176	0.0028	0.09	4.4E-10	40

BMI	rs11047132	12:24008435	G	0.0227	0.0031	0.11	1.6E-13	84
BMI	rs1106761	8:142619234	A	0.0134	0.0020	0.35	1.8E-11	66
BMI	rs1106908	17:34942595	G	0.0161	0.0016	0.59	7.6E-23	101
BMI	rs11075489	16:62803841	C	0.0116	0.0017	0.54	1.2E-11	54
BMI	rs11105839	12:91237920	T	0.0113	0.0017	0.63	1.3E-11	48
BMI	rs11115176	12:82465797	T	0.0131	0.0019	0.78	6.8E-12	48
BMI	rs11121210	1:8708529	C	0.0111	0.0017	0.66	2.4E-10	45
BMI	rs11170468	12:39430048	A	0.0130	0.0019	0.81	1.1E-11	42
BMI	rs11218510	11:121922587	G	0.0140	0.0020	0.63	6.8E-13	73
BMI	rs112566467	1:39562627	T	0.0184	0.0024	0.20	5.9E-15	86
BMI	rs11525873	7:138817193	T	0.0232	0.0032	0.86	3.0E-13	102
BMI	rs11577094	1:38026600	T	0.0186	0.0030	0.08	3.3E-10	43
BMI	rs11611246	12:939480	T	0.0223	0.0020	0.22	2.0E-28	136
BMI	rs11633626	15:95271378	C	0.0157	0.0018	0.42	7.3E-19	97
BMI	rs11636611	15:36391965	T	0.0104	0.0017	0.49	8.9E-10	44
BMI	rs11655587	17:47140794	C	0.0210	0.0020	0.66	6.9E-26	160
BMI	rs11672660	19:46180184	C	0.0338	0.0021	0.76	6.8E-60	335
BMI	rs1167821	7:75150312	T	0.0201	0.0019	0.42	9.4E-26	158
BMI	rs11692326	2:208263279	T	0.0147	0.0019	0.22	1.7E-14	59
BMI	rs11739877	5:105876806	T	0.0116	0.0018	0.56	4.0E-11	53
BMI	rs11757278	6:13180454	T	0.0133	0.0019	0.64	6.9E-13	66
BMI	rs11836108	12:133391022	A	0.0113	0.0018	0.26	8.4E-10	40
BMI	rs11856579	15:78012688	G	0.0161	0.0019	0.78	1.4E-16	72
BMI	rs11866815	16:387867	C	0.0153	0.0019	0.78	2.2E-16	66
BMI	rs11915371	3:70539559	C	0.0154	0.0021	0.20	2.3E-13	60
BMI	rs12049202	1:77967523	T	0.0239	0.0021	0.26	3.4E-29	175
BMI	rs12072739	1:98315893	G	0.0169	0.0023	0.29	1.5E-13	94
BMI	rs12098284	10:76047464	T	0.0184	0.0026	0.10	9.9E-13	49

BMI	rs12121950	1:49710264	T	0.0185	0.0018	0.38	7.1E-24	130
BMI	rs12140153	1:62579891	G	0.0353	0.0034	0.92	1.4E-25	142
BMI	rs12151152	19:47563532	G	0.0210	0.0020	0.56	6.5E-27	175
BMI	rs12206094	6:108906200	C	0.0143	0.0018	0.73	1.9E-15	64
BMI	rs12282785	11:76476030	C	0.0157	0.0023	0.79	1.4E-11	67
BMI	rs12286929	11:115022404	G	0.0177	0.0016	0.52	1.9E-27	126
BMI	rs12321904	12:109989228	T	0.0096	0.0016	0.47	2.7E-09	37
BMI	rs12364470	11:134601012	G	0.0187	0.0022	0.21	2.2E-17	92
BMI	rs12369179	12:122963550	C	0.0340	0.0031	0.92	2.3E-28	138
BMI	rs12422552	12:14413931	G	0.0131	0.0019	0.76	1.8E-11	50
BMI	rs12429545	13:54102206	A	0.0313	0.0024	0.14	1.4E-37	193
BMI	rs12438629	15:62122539	C	0.0347	0.0052	0.95	2.1E-11	93
BMI	rs12449219	16:77261943	G	0.0164	0.0026	0.09	1.4E-10	36
BMI	rs12462975	19:30272202	A	0.0193	0.0018	0.31	1.5E-25	130
BMI	rs12509234	4:120319434	C	0.0116	0.0019	0.25	3.9E-10	40
BMI	rs12628051	22:40654276	T	0.0161	0.0018	0.62	2.9E-19	98
BMI	rs12628891	22:38317137	C	0.0115	0.0019	0.72	5.9E-10	43
BMI	rs12635553	3:171113714	A	0.0099	0.0017	0.51	4.1E-09	40
BMI	rs12680842	8:95582606	A	0.0142	0.0017	0.61	3.4E-16	77
BMI	rs12681792	8:62054463	A	0.0150	0.0021	0.29	2.9E-12	74
BMI	rs1269175	6:126040435	A	0.0105	0.0017	0.52	8.1E-10	44
BMI	rs12692596	2:161265910	T	0.0120	0.0017	0.35	1.0E-12	53
BMI	rs12714199	2:86812549	C	0.0141	0.0017	0.40	3.2E-16	77
BMI	rs12765914	10:34013507	T	0.0226	0.0031	0.11	2.0E-13	78
BMI	rs1277733	10:18562538	T	0.0120	0.0020	0.78	4.3E-09	40
BMI	rs12868881	13:112218924	A	0.0138	0.0017	0.45	1.4E-15	76
BMI	rs12922346	16:82438337	C	0.0133	0.0020	0.23	1.5E-11	50
BMI	rs12939549	17:78611724	A	0.0180	0.0016	0.62	3.7E-28	123

BMI	rs13002946	2:100801959	T	0.0177	0.0019	0.77	3.9E-20	89
BMI	rs13021737	2:632348	G	0.0578	0.0021	0.82	2.9E-161	785
BMI	rs13033310	2:133523605	A	0.0146	0.0022	0.25	3.4E-11	64
BMI	rs13107325	4:103188709	T	0.0468	0.0032	0.05	3.8E-47	155
BMI	rs13110266	4:162129844	G	0.0124	0.0016	0.58	4.0E-14	60
BMI	rs13174863	5:139080745	G	0.0197	0.0023	0.15	1.9E-17	78
BMI	rs13191362	6:163033350	A	0.0235	0.0025	0.91	4.1E-21	71
BMI	rs1320251	17:21264396	C	0.0177	0.0017	0.51	5.5E-25	126
BMI	rs1346841	4:65651730	G	0.0126	0.0017	0.63	3.2E-13	60
BMI	rs1356506	18:40708038	T	0.0137	0.0018	0.59	8.4E-15	73
BMI	rs1383592	8:106430676	A	0.0122	0.0021	0.26	4.9E-09	46
BMI	rs140733155	21:48048773	G	0.0559	0.0094	0.02	3.0E-09	110
BMI	rs1409818	20:21381121	T	0.0195	0.0028	0.09	2.6E-12	51
BMI	rs1431659	8:73439070	A	0.0191	0.0019	0.27	2.3E-23	115
BMI	rs1436344	3:104606144	C	0.0147	0.0017	0.61	1.1E-17	83
BMI	rs1437842	4:173597016	G	0.0106	0.0017	0.58	8.5E-10	44
BMI	rs1451077	2:147901207	G	0.0169	0.0019	0.45	1.4E-18	114
BMI	rs1451109	4:137071335	G	0.0158	0.0017	0.34	1.4E-19	90
BMI	rs1470545	2:205365851	T	0.0370	0.0043	0.04	5.6E-18	87
BMI	rs1471212	15:31857494	A	0.0109	0.0017	0.46	1.5E-10	48
BMI	rs1481012	4:89039082	A	0.0188	0.0026	0.91	5.1E-13	48
BMI	rs1491905	14:47296286	T	0.0147	0.0017	0.46	3.9E-18	87
BMI	rs1501673	5:87963600	A	0.0289	0.0025	0.13	2.7E-31	154
BMI	rs1522569	4:171632637	T	0.0141	0.0022	0.86	1.6E-10	38
BMI	rs1536053	13:111982291	C	0.0122	0.0019	0.71	8.0E-11	50
BMI	rs1561554	2:35409697	G	0.0115	0.0017	0.35	4.9E-11	48
BMI	rs1658820	8:4288577	T	0.0132	0.0020	0.27	3.8E-11	55
BMI	rs16851483	3:141275436	T	0.0352	0.0034	0.07	4.9E-25	125

BMI	rs16989232	20:39291784	A	0.0115	0.0017	0.37	6.2E-12	50
BMI	rs17001561	4:77096118	A	0.0145	0.0023	0.13	1.8E-10	37
BMI	rs17024393	1:110154688	C	0.0644	0.0049	0.05	7.1E-39	314
BMI	rs17201143	20:50402079	C	0.0112	0.0018	0.70	1.1E-09	43
BMI	rs17276464	4:153028860	T	0.0096	0.0016	0.40	4.8E-09	36
BMI	rs1730859	1:107617707	G	0.0120	0.0017	0.31	3.0E-12	50
BMI	rs17405819	8:76806584	T	0.0211	0.0018	0.71	6.0E-33	149
BMI	rs17636031	10:126594078	C	0.0154	0.0018	0.23	3.9E-17	68
BMI	rs17681451	3:114399296	G	0.0225	0.0031	0.93	7.4E-13	52
BMI	rs17806224	20:51065854	G	0.0260	0.0022	0.84	7.9E-32	147
BMI	rs1852006	7:77829768	G	0.0153	0.0018	0.63	6.7E-18	88
BMI	rs1884389	20:1410582	C	0.0108	0.0017	0.56	3.7E-10	46
BMI	rs1884429	1:11112836	T	0.0134	0.0019	0.23	2.3E-12	52
BMI	rs1884897	20:6612832	G	0.0184	0.0017	0.65	2.7E-28	124
BMI	rs1899689	7:121964349	T	0.0120	0.0017	0.40	4.2E-13	56
BMI	rs1916801	3:61187046	A	0.0169	0.0017	0.57	1.9E-24	113
BMI	rs1927790	13:96922191	C	0.0140	0.0016	0.46	1.6E-17	79
BMI	rs1928295	9:120378483	T	0.0134	0.0016	0.52	2.2E-16	72
BMI	rs194809	16:23804956	A	0.0126	0.0022	0.18	4.9E-09	38
BMI	rs1951455	14:91512339	C	0.0148	0.0019	0.72	6.1E-15	71
BMI	rs1954494	14:62593297	T	0.0097	0.0016	0.54	2.4E-09	38
BMI	rs1982350	11:13350131	G	0.0156	0.0018	0.56	2.0E-18	97
BMI	rs1982441	8:28021769	T	0.0170	0.0025	0.17	2.2E-11	65
BMI	rs1996120	15:68181534	G	0.0167	0.0016	0.64	3.9E-24	103
BMI	rs2051559	4:3298800	C	0.0167	0.0025	0.12	3.8E-11	48
BMI	rs2053682	5:170599327	A	0.0170	0.0018	0.66	2.6E-20	104
BMI	rs2065418	11:30422068	T	0.0139	0.0018	0.68	6.1E-15	68
BMI	rs2112347	5:75015242	T	0.0276	0.0017	0.59	1.2E-61	297

BMI	rs2134858	9:73837155	C	0.0117	0.0017	0.50	5.9E-12	55
BMI	rs215614	7:32347335	G	0.0144	0.0018	0.33	2.8E-16	74
BMI	rs217669	14:62360075	C	0.0172	0.0021	0.25	6.3E-16	89
BMI	rs2183824	9:28412078	T	0.0234	0.0017	0.35	2.3E-41	201
BMI	rs2192158	4:55505360	A	0.0137	0.0017	0.48	7.4E-16	76
BMI	rs2196618	8:85089437	G	0.0137	0.0019	0.72	1.5E-12	60
BMI	rs2206277	6:50798526	T	0.0408	0.0021	0.21	1.8E-83	441
BMI	rs2228213	6:12124855	G	0.0144	0.0017	0.66	5.5E-17	75
BMI	rs2246012	6:131898208	C	0.0161	0.0022	0.22	1.1E-13	71
BMI	rs2342892	16:24540806	T	0.0126	0.0017	0.47	1.3E-13	64
BMI	rs2357760	6:120213880	A	0.0143	0.0017	0.66	2.1E-16	74
BMI	rs2391540	4:130726833	T	0.0139	0.0018	0.31	1.4E-14	67
BMI	rs2396625	7:113028634	T	0.0176	0.0017	0.61	2.8E-24	119
BMI	rs2400414	1:194965200	C	0.0126	0.0018	0.66	5.5E-13	57
BMI	rs2439823	10:99778226	G	0.0165	0.0017	0.50	6.5E-22	110
BMI	rs2601777	16:4035068	G	0.0143	0.0017	0.64	2.8E-17	76
BMI	rs2605603	11:93221105	G	0.0103	0.0016	0.48	2.0E-10	43
BMI	rs2619976	17:71754545	T	0.0105	0.0018	0.38	2.0E-09	42
BMI	rs2622274	6:64240516	G	0.0107	0.0017	0.59	3.2E-10	45
BMI	rs264962	2:104308545	C	0.0119	0.0017	0.55	1.7E-12	57
BMI	rs2715423	15:99511873	G	0.0115	0.0019	0.72	1.9E-09	43
BMI	rs273505	19:18217147	C	0.0173	0.0019	0.46	3.3E-19	120
BMI	rs2777768	9:84186734	A	0.0119	0.0019	0.78	6.4E-10	39
BMI	rs2820295	1:201800868	A	0.0235	0.0018	0.30	5.6E-39	188
BMI	rs2832283	21:30690558	A	0.0115	0.0020	0.24	4.7E-09	39
BMI	rs28350	3:42418446	A	0.0172	0.0022	0.19	1.1E-14	74
BMI	rs2861089	5:164557954	A	0.0105	0.0017	0.36	1.6E-09	41
BMI	rs2861685	2:67837553	T	0.0165	0.0019	0.59	7.8E-18	107

BMI	rs2862996	11:43653833	G	0.0216	0.0017	0.31	3.6E-35	161
BMI	rs288230	5:107422067	T	0.0238	0.0023	0.83	4.7E-26	131
BMI	rs2907948	7:150638484	G	0.0145	0.0019	0.81	1.9E-14	52
BMI	rs2968487	1:96887370	T	0.0176	0.0018	0.26	7.6E-22	95
BMI	rs3101336	1:72751185	C	0.0254	0.0016	0.63	4.8E-54	242
BMI	rs34277166	10:102634853	A	0.0144	0.0019	0.43	8.3E-14	82
BMI	rs34811474	4:25408838	G	0.0293	0.0023	0.78	8.5E-38	237
BMI	rs349088	11:84814393	C	0.0130	0.0017	0.44	3.5E-14	67
BMI	rs354508	8:15535226	C	0.0146	0.0023	0.84	2.7E-10	46
BMI	rs355777	3:154034950	C	0.0151	0.0017	0.42	2.1E-18	90
BMI	rs35851183	4:80717182	G	0.0121	0.0020	0.32	1.1E-09	51
BMI	rs36061954	8:38329650	T	0.0119	0.0019	0.39	1.1E-09	55
BMI	rs3739514	9:133783025	A	0.0132	0.0020	0.39	6.6E-11	67
BMI	rs3744017	17:73871467	A	0.0139	0.0022	0.16	2.4E-10	41
BMI	rs3770799	2:36788616	G	0.0114	0.0018	0.37	1.2E-10	49
BMI	rs3806114	6:20482335	G	0.0120	0.0018	0.27	1.5E-11	46
BMI	rs3808477	8:116670347	C	0.0182	0.0019	0.71	8.7E-22	110
BMI	rs3811125	9:94187247	C	0.0152	0.0021	0.77	9.4E-13	67
BMI	rs3825061	11:118944675	T	0.0140	0.0017	0.32	6.2E-16	69
BMI	rs3923783	17:1843189	C	0.0222	0.0022	0.79	4.1E-23	131
BMI	rs3930349	17:31475545	C	0.0140	0.0020	0.75	7.7E-12	59
BMI	rs39654	3:173095123	G	0.0163	0.0017	0.58	1.8E-21	104
BMI	rs4097319	10:33860515	T	0.0107	0.0017	0.57	4.3E-10	45
BMI	rs4123853	8:14091025	T	0.0140	0.0017	0.32	1.1E-15	69
BMI	rs41284828	13:50963685	G	0.0341	0.0057	0.95	3.0E-09	97
BMI	rs4256980	11:8673939	G	0.0187	0.0017	0.63	8.6E-29	131
BMI	rs427943	21:46570896	C	0.0177	0.0017	0.57	3.6E-25	124
BMI	rs4290163	10:104610926	T	0.0130	0.0017	0.40	3.5E-15	66

BMI	rs4307239	7:24354300	G	0.0115	0.0017	0.47	1.5E-11	53
BMI	rs4421883	13:79561962	C	0.0103	0.0017	0.56	1.8E-09	42
BMI	rs459552	5:112176756	T	0.0133	0.0019	0.28	8.4E-12	57
BMI	rs4609871	16:29932064	T	0.0219	0.0017	0.50	6.6E-38	194
BMI	rs4671328	2:58935282	T	0.0214	0.0017	0.45	3.4E-36	183
BMI	rs4700608	5:63026280	C	0.0155	0.0017	0.55	4.3E-20	96
BMI	rs4721089	7:1872921	T	0.0167	0.0023	0.73	5.6E-13	89
BMI	rs4740619	9:15634326	T	0.0189	0.0016	0.51	3.2E-31	144
BMI	rs4834272	4:113313986	C	0.0108	0.0017	0.35	3.5E-10	43
BMI	rs4841504	8:11024663	C	0.0166	0.0016	0.56	5.4E-24	110
BMI	rs4857968	3:20714580	G	0.0125	0.0018	0.76	6.5E-12	47
BMI	rs487152	6:160774486	A	0.0108	0.0016	0.48	3.0E-11	47
BMI	rs4880341	10:133992689	C	0.0130	0.0017	0.47	3.1E-14	68
BMI	rs4906263	14:103249127	G	0.0176	0.0018	0.35	8.1E-23	113
BMI	rs4973618	2:229002620	G	0.0148	0.0018	0.33	1.3E-16	78
BMI	rs5215	11:17408630	T	0.0113	0.0017	0.56	1.2E-11	51
BMI	rs543874	1:177889480	G	0.0479	0.0020	0.18	3.1E-125	555
BMI	rs561136	1:23345051	C	0.0186	0.0024	0.15	1.4E-14	70
BMI	rs56356382	19:4064057	T	0.0217	0.0024	0.84	3.2E-19	104
BMI	rs57800857	4:140863365	A	0.0157	0.0020	0.66	3.5E-15	90
BMI	rs592483	11:69445173	C	0.0137	0.0017	0.51	1.5E-16	76
BMI	rs59302296	3:9507314	A	0.0217	0.0032	0.08	9.1E-12	54
BMI	rs594821	18:76745589	C	0.0191	0.0030	0.91	3.2E-10	47
BMI	rs615568	20:3008775	G	0.0101	0.0017	0.47	3.8E-09	41
BMI	rs61813324	1:156049877	T	0.0289	0.0028	0.15	3.2E-24	172
BMI	rs61828641	1:174321997	A	0.0223	0.0030	0.10	2.5E-13	75
BMI	rs6265	11:27679916	C	0.0413	0.0021	0.83	7.4E-89	397
BMI	rs6443750	3:181329682	C	0.0152	0.0021	0.80	7.3E-13	59

BMI	rs6451675	5:43110855	G	0.0140	0.0018	0.60	2.5E-14	76
BMI	rs6463489	7:5542513	T	0.0167	0.0026	0.10	2.5E-10	39
BMI	rs6476617	9:37200103	G	0.0149	0.0017	0.68	1.5E-17	78
BMI	rs6477694	9:111932342	C	0.0126	0.0017	0.40	6.6E-14	62
BMI	rs6493498	15:51754451	T	0.0137	0.0016	0.43	4.8E-17	74
BMI	rs650198	12:69674595	C	0.0137	0.0019	0.33	6.4E-13	67
BMI	rs6512302	20:62691550	C	0.0134	0.0020	0.77	1.5E-11	52
BMI	rs6539064	12:103706754	C	0.0194	0.0019	0.70	1.1E-23	127
BMI	rs6545714	2:59307725	G	0.0194	0.0016	0.40	4.0E-32	145
BMI	rs6556301	5:176527577	G	0.0113	0.0017	0.63	8.1E-11	48
BMI	rs6567160	18:57829135	C	0.0552	0.0019	0.22	7.8E-184	835
BMI	rs6591407	11:56914157	C	0.0124	0.0021	0.84	3.6E-09	33
BMI	rs6607337	17:35057373	C	0.0124	0.0019	0.73	2.5E-11	49
BMI	rs6661316	1:210095527	T	0.0120	0.0016	0.52	1.7E-13	58
BMI	rs6690398	1:66447394	A	0.0125	0.0017	0.58	1.9E-13	61
BMI	rs6720868	2:230663576	T	0.0154	0.0018	0.31	1.8E-17	82
BMI	rs6804181	3:116937546	A	0.0141	0.0023	0.79	6.5E-10	54
BMI	rs6804842	3:25106437	G	0.0141	0.0016	0.54	7.6E-18	80
BMI	rs6850421	4:180187034	A	0.0113	0.0019	0.51	3.7E-09	51
BMI	rs6864049	5:124330522	G	0.0121	0.0016	0.57	1.5E-13	58
BMI	rs6890310	5:27193573	G	0.0119	0.0019	0.75	3.3E-10	43
BMI	rs6922607	6:142703483	G	0.0130	0.0022	0.18	1.9E-09	41
BMI	rs6932930	6:34677103	G	0.0257	0.0019	0.25	1.2E-40	202
BMI	rs6962280	7:44788657	G	0.0140	0.0019	0.47	3.4E-13	79
BMI	rs698147	5:3513485	A	0.0116	0.0017	0.48	9.7E-12	54
BMI	rs7024334	9:109072075	T	0.0135	0.0020	0.22	4.7E-12	51
BMI	rs7037266	9:6942940	C	0.0106	0.0018	0.60	1.2E-09	43
BMI	rs704061	12:89771903	C	0.0142	0.0017	0.41	8.3E-17	79

BMI	rs7070670	10:61842645	C	0.0126	0.0021	0.71	7.1E-10	52
BMI	rs7084454	10:21821274	A	0.0198	0.0018	0.30	4.5E-27	134
BMI	rs709400	14:104149475	A	0.0148	0.0017	0.68	5.7E-19	77
BMI	rs7124681	11:47529947	A	0.0257	0.0016	0.39	4.0E-55	254
BMI	rs7138803	12:50247468	A	0.0297	0.0017	0.38	3.1E-71	335
BMI	rs7144011	14:79940383	T	0.0263	0.0020	0.23	2.4E-40	196
BMI	rs7181610	15:35826859	A	0.0146	0.0025	0.90	4.5E-09	30
BMI	rs7206608	16:82872628	G	0.0130	0.0018	0.34	1.2E-12	61
BMI	rs7238896	18:1840658	G	0.0218	0.0028	0.16	2.5E-15	104
BMI	rs7239114	18:45921214	A	0.0120	0.0017	0.53	6.2E-13	58
BMI	rs72673947	8:118884379	G	0.0223	0.0031	0.12	4.1E-13	83
BMI	rs7357754	9:92207308	G	0.0120	0.0017	0.50	1.8E-12	58
BMI	rs740157	7:77055885	A	0.0118	0.0016	0.46	4.8E-13	56
BMI	rs7498665	16:28883241	G	0.0285	0.0017	0.35	1.1E-66	298
BMI	rs7534091	1:118864616	G	0.0120	0.0018	0.25	6.7E-11	44
BMI	rs7561278	2:48954905	T	0.0169	0.0021	0.77	4.9E-16	83
BMI	rs7570446	2:193801010	A	0.0111	0.0019	0.55	4.7E-09	49
BMI	rs7575118	2:182653725	T	0.0140	0.0024	0.14	3.3E-09	37
BMI	rs7588437	2:181575281	G	0.0165	0.0017	0.68	2.3E-22	95
BMI	rs7599312	2:213413231	G	0.0182	0.0018	0.72	1.5E-23	109
BMI	rs7607490	2:12851120	A	0.0164	0.0027	0.09	1.2E-09	34
BMI	rs7616009	3:194881756	G	0.0157	0.0024	0.86	4.3E-11	48
BMI	rs7631156	3:131751628	A	0.0215	0.0018	0.29	3.3E-32	155
BMI	rs765875	6:143185683	C	0.0132	0.0017	0.46	1.1E-14	70
BMI	rs7678054	4:95093855	G	0.0099	0.0017	0.57	4.6E-09	39
BMI	rs76942203	11:116973247	A	0.0263	0.0041	0.07	9.1E-11	75
BMI	rs7713317	5:95716722	G	0.0166	0.0018	0.31	2.0E-20	94
BMI	rs7734385	5:158460212	G	0.0101	0.0016	0.52	6.1E-10	41

BMI	rs77432547	13:86494817	G	0.0170	0.0021	0.33	1.4E-15	103
BMI	rs7769594	6:83447813	T	0.0164	0.0023	0.14	2.1E-12	54
BMI	rs7777084	7:93089243	A	0.0131	0.0017	0.42	1.6E-14	68
BMI	rs7802342	7:137435925	G	0.0124	0.0019	0.27	6.2E-11	49
BMI	rs7826312	8:32400115	C	0.0109	0.0016	0.57	3.2E-11	47
BMI	rs7893571	10:16750129	T	0.0125	0.0018	0.69	5.8E-12	54
BMI	rs7899106	10:87410904	G	0.0327	0.0037	0.05	1.7E-18	74
BMI	rs7903146	10:114758349	C	0.0178	0.0018	0.77	1.7E-23	92
BMI	rs79113395	1:1590521	G	0.0200	0.0022	0.77	2.0E-20	115
BMI	rs7944782	11:130795698	G	0.0144	0.0017	0.52	3.6E-17	83
BMI	rs7975187	12:60964108	G	0.0137	0.0021	0.21	3.9E-11	50
BMI	rs8024806	15:53473990	T	0.0254	0.0035	0.94	6.7E-13	56
BMI	rs8075273	17:61728881	C	0.0137	0.0018	0.76	4.7E-14	55
BMI	rs8122855	20:25192049	A	0.0137	0.0018	0.34	4.1E-14	68
BMI	rs8134638	21:40644170	C	0.0133	0.0020	0.47	1.6E-11	71
BMI	rs816367	6:53995542	G	0.0107	0.0017	0.34	1.0E-09	41
BMI	rs8181823	13:65477940	C	0.0125	0.0020	0.73	4.4E-10	49
BMI	rs845084	10:125220036	A	0.0136	0.0019	0.27	3.2E-12	59
BMI	rs867560	9:129465233	G	0.0136	0.0017	0.53	1.2E-15	74
BMI	rs872281	14:40834177	C	0.0152	0.0023	0.78	1.6E-11	64
BMI	rs889398	16:69556715	C	0.0195	0.0016	0.59	3.2E-32	148
BMI	rs891387	18:21103909	T	0.0208	0.0017	0.49	9.3E-35	175
BMI	rs930295	2:50233352	A	0.0208	0.0023	0.15	2.0E-19	90
BMI	rs9320823	6:98429337	C	0.0165	0.0017	0.66	2.1E-21	99
BMI	rs942066	14:94031914	G	0.0202	0.0020	0.61	2.4E-24	157
BMI	rs946824	1:243684019	T	0.0197	0.0025	0.16	4.8E-15	83
BMI	rs9540493	13:66205704	A	0.0129	0.0017	0.50	7.8E-15	67
BMI	rs9595908	13:33184288	T	0.0154	0.0017	0.60	3.7E-20	92

BMI	rs9603697	13:40783323	T	0.0134	0.0018	0.38	1.7E-13	68
BMI	rs9808302	2:236854450	G	0.0116	0.0019	0.50	9.0E-10	54
BMI	rs9816226	3:185834499	T	0.0315	0.0021	0.82	1.5E-50	236
BMI	rs9818122	3:85861064	C	0.0228	0.0020	0.18	4.0E-30	125
BMI	rs9826775	3:156295341	A	0.0155	0.0024	0.85	6.6E-11	51
BMI	rs9862795	3:49915506	T	0.0264	0.0019	0.42	4.4E-44	275
BMI	rs9937053	16:53799507	A	0.0721	0.0016	0.42	1.0E-200	2053
WHR	rs10019888	4:26062990	A	-0.0208	0.0023	0.85	8.0E-20	87
WHR	rs10049088	3:156797648	T	-0.0271	0.0018	0.34	1.5E-53	267
WHR	rs10132280	14:25928179	A	-0.0116	0.0018	0.32	3.1E-10	48
WHR	rs10153926	2:43189120	A	0.0143	0.0024	0.24	2.0E-09	59
WHR	rs10164099	18:34690744	T	-0.0149	0.0025	0.85	1.2E-09	45
WHR	rs1017698	2:219170525	A	-0.0113	0.0018	0.65	5.1E-10	47
WHR	rs1020731	2:161144055	A	0.0127	0.0019	0.73	5.8E-11	51
WHR	rs1045241	5:118729286	T	-0.0123	0.0019	0.30	8.9E-11	51
WHR	rs1045411	13:31033232	T	-0.0155	0.0019	0.31	1.3E-15	82
WHR	rs10475249	5:4010135	C	0.0134	0.0020	0.50	1.3E-11	72
WHR	rs10490869	3:35635145	A	-0.0183	0.0022	0.79	9.3E-17	90
WHR	rs10499013	6:97946396	A	-0.0125	0.0020	0.27	5.1E-10	50
WHR	rs10507223	12:108455828	C	0.0134	0.0022	0.79	5.1E-10	48
WHR	rs10745659	12:94092690	C	-0.0127	0.0018	0.56	1.4E-12	64
WHR	rs10761785	10:65318766	T	-0.0147	0.0017	0.52	6.4E-18	87
WHR	rs10788569	10:89604732	T	-0.0134	0.0019	0.74	4.0E-12	56
WHR	rs10827252	10:33672884	A	-0.0113	0.0018	0.49	2.0E-10	52
WHR	rs10842707	12:26471364	T	0.0266	0.0020	0.26	3.9E-39	218
WHR	rs10851523	15:53044002	C	-0.0118	0.0020	0.31	1.8E-09	48
WHR	rs10876528	12:54421476	A	0.0236	0.0018	0.39	8.6E-39	215
WHR	rs10896012	11:65278461	T	-0.0180	0.0022	0.86	2.1E-16	63

WHR	rs10919388	1:170372503	A	-0.0266	0.0019	0.27	6.4E-43	227
WHR	rs10923724	1:119546842	T	0.0240	0.0017	0.62	1.8E-45	219
WHR	rs10980797	9:113912553	A	-0.0154	0.0020	0.54	3.9E-15	95
WHR	rs10991433	9:107726918	T	-0.0254	0.0028	0.90	5.7E-20	90
WHR	rs11030108	11:27695464	A	0.0154	0.0018	0.33	1.2E-17	84
WHR	rs11055887	12:14417179	A	-0.0133	0.0023	0.19	4.3E-09	44
WHR	rs11176015	12:66441684	T	0.0151	0.0020	0.27	7.2E-14	73
WHR	rs11187537	10:95346805	C	0.0130	0.0020	0.27	1.7E-10	53
WHR	rs11216183	11:116781545	A	0.0226	0.0034	0.12	2.7E-11	89
WHR	rs1122080	5:158015903	A	-0.0131	0.0022	0.17	1.6E-09	39
WHR	rs112266013	6:15230743	A	-0.0167	0.0028	0.12	3.2E-09	49
WHR	rs11231084	11:62177643	C	-0.0117	0.0020	0.46	2.4E-09	55
WHR	rs1124639	2:200775744	T	-0.0113	0.0017	0.50	7.5E-11	52
WHR	rs1139653	16:4484396	A	0.0147	0.0020	0.30	1.1E-13	74
WHR	rs1142	7:104756326	T	0.0149	0.0018	0.32	2.1E-17	78
WHR	rs114760566	6:34192036	A	0.0674	0.0048	0.03	2.9E-44	195
WHR	rs1158805	18:40736590	A	-0.0133	0.0018	0.42	4.9E-13	70
WHR	rs1163627	13:112225701	A	0.0104	0.0017	0.60	3.0E-09	42
WHR	rs11654387	17:68446861	C	0.0161	0.0018	0.56	1.5E-19	103
WHR	rs11724804	4:965779	A	-0.0165	0.0018	0.45	5.0E-20	109
WHR	rs11747001	5:132412299	A	0.0154	0.0020	0.74	8.4E-15	73
WHR	rs11764879	7:77333267	A	-0.0117	0.0019	0.26	3.9E-10	43
WHR	rs11878507	19:18837823	A	-0.0119	0.0020	0.54	1.4E-09	57
WHR	rs11897119	2:66772000	T	-0.0134	0.0017	0.58	4.1E-15	70
WHR	rs1190982	14:58815839	T	0.0157	0.0019	0.36	5.1E-16	91
WHR	rs11992444	8:25464690	T	0.0185	0.0020	0.53	5.0E-21	138
WHR	rs12024554	1:19925759	T	-0.0149	0.0023	0.23	1.1E-10	64
WHR	rs12042959	1:243533273	A	0.0155	0.0025	0.85	9.3E-10	50

WHR	rs12101393	15:92570921	C	0.0140	0.0022	0.76	1.2E-10	57
WHR	rs12138803	1:172348823	T	0.0198	0.0019	0.24	7.2E-25	116
WHR	rs12140153	1:62579891	T	-0.0214	0.0035	0.08	7.6E-10	52
WHR	rs12206094	6:108906200	T	-0.0119	0.0019	0.27	2.2E-10	45
WHR	rs12287076	11:47606865	C	0.0192	0.0020	0.73	3.5E-21	118
WHR	rs12430764	13:93896935	A	0.0107	0.0018	0.48	1.9E-09	46
WHR	rs1243188	10:21908803	T	-0.0148	0.0019	0.71	2.4E-14	72
WHR	rs12440605	15:42102285	A	0.0118	0.0018	0.52	4.0E-11	56
WHR	rs12440695	15:62435156	T	-0.0103	0.0018	0.66	4.4E-09	38
WHR	rs12449442	17:65947640	A	0.0172	0.0021	0.23	2.6E-16	86
WHR	rs12459350	19:2176586	A	0.0134	0.0017	0.53	2.2E-15	72
WHR	rs12469667	2:166162705	A	-0.0130	0.0021	0.27	2.8E-10	53
WHR	rs12494190	3:168949384	A	-0.0121	0.0018	0.40	3.0E-11	57
WHR	rs12495178	3:85886077	T	0.0124	0.0018	0.59	1.3E-12	60
WHR	rs12527712	6:80916967	T	0.0284	0.0033	0.08	3.4E-18	93
WHR	rs12575252	11:8694073	C	-0.0154	0.0018	0.36	2.3E-18	89
WHR	rs12590238	14:103350197	C	-0.0141	0.0023	0.79	1.5E-09	53
WHR	rs12593088	15:81058640	A	-0.0128	0.0019	0.29	5.2E-12	54
WHR	rs12608504	19:18389135	A	0.0251	0.0018	0.35	4.7E-46	231
WHR	rs12629247	3:187632584	C	0.0289	0.0047	0.05	9.8E-10	67
WHR	rs12631066	3:33872787	C	0.0124	0.0021	0.23	2.1E-09	44
WHR	rs12684047	9:111972671	A	-0.0185	0.0023	0.19	3.4E-15	84
WHR	rs12692387	2:9698190	T	-0.0124	0.0021	0.43	2.9E-09	61
WHR	rs12774134	10:4963327	T	-0.0158	0.0026	0.13	2.1E-09	45
WHR	rs12777288	10:115860058	T	-0.0139	0.0021	0.79	6.9E-11	51
WHR	rs12828016	12:998365	T	-0.0114	0.0018	0.33	4.1E-10	47
WHR	rs1294436	6:6746166	C	0.0230	0.0020	0.59	1.3E-30	206
WHR	rs12986231	19:18469017	T	0.0150	0.0022	0.74	2.0E-11	70

WHR	rs13028903	2:59951465	T	0.0113	0.0018	0.41	2.8E-10	50
WHR	rs13063979	3:131564741	T	-0.0135	0.0019	0.74	1.2E-12	56
WHR	rs13130484	4:45175691	T	0.0147	0.0017	0.45	7.8E-18	86
WHR	rs13229637	7:136717447	T	0.0159	0.0025	0.82	1.9E-10	61
WHR	rs13255070	8:68203608	A	-0.0136	0.0022	0.75	5.1E-10	56
WHR	rs13256367	8:128334900	A	0.0141	0.0019	0.68	5.4E-14	70
WHR	rs1328757	20:56135199	T	0.0114	0.0018	0.45	1.7E-10	52
WHR	rs13333747	16:2175373	T	0.0180	0.0025	0.83	1.4E-12	75
WHR	rs1334576	6:7211818	A	-0.0162	0.0018	0.47	1.7E-19	106
WHR	rs1345203	2:112253851	T	0.0187	0.0024	0.80	3.1E-15	90
WHR	rs13642	11:30432220	A	0.0113	0.0019	0.68	9.5E-10	45
WHR	rs1382894	5:76599022	A	0.0114	0.0019	0.65	9.2E-10	48
WHR	rs1385167	2:66200648	A	-0.0224	0.0024	0.82	6.2E-20	121
WHR	rs1406948	20:33905619	A	-0.0137	0.0017	0.43	4.1E-15	74
WHR	rs1431659	8:73439070	A	0.0143	0.0020	0.27	1.1E-12	65
WHR	rs1452075	3:62481063	T	0.0123	0.0019	0.70	1.3E-10	51
WHR	rs1458156	12:41887940	T	0.0125	0.0018	0.48	1.9E-12	63
WHR	rs1494204	10:27904321	T	-0.0106	0.0018	0.36	4.4E-09	42
WHR	rs15285	8:19824667	T	-0.0117	0.0019	0.27	1.6E-09	43
WHR	rs1534696	7:26397239	A	-0.0226	0.0017	0.56	3.7E-39	203
WHR	rs155524	3:37562141	A	0.0134	0.0018	0.58	1.3E-13	71
WHR	rs1563355	1:219653101	T	-0.0277	0.0019	0.34	1.6E-49	276
WHR	rs1569135	2:188115398	A	0.0215	0.0017	0.56	2.8E-37	184
WHR	rs16976932	15:56781255	A	0.0186	0.0027	0.08	1.0E-11	41
WHR	rs17101456	10:122875040	A	-0.0206	0.0028	0.86	7.5E-14	82
WHR	rs17109256	14:79939993	A	0.0169	0.0021	0.23	2.4E-16	81
WHR	rs17324331	2:230739209	C	0.0120	0.0019	0.31	1.8E-10	50
WHR	rs17326656	2:48962291	T	0.0153	0.0021	0.18	4.2E-13	56

WHR	rs17448885	6:97385975	C	0.0110	0.0019	0.60	3.3E-09	47
WHR	rs17513613	19:30286822	T	-0.0125	0.0018	0.67	5.4E-12	56
WHR	rs1757471	10:34168090	T	0.0128	0.0018	0.55	5.9E-13	65
WHR	rs17644283	4:26308792	A	0.0148	0.0018	0.37	3.2E-17	83
WHR	rs17651507	17:44059010	A	0.0187	0.0020	0.73	1.9E-20	111
WHR	rs17738166	5:172997978	A	0.0114	0.0018	0.41	3.8E-10	51
WHR	rs1787013	18:13072979	T	-0.0108	0.0018	0.57	1.3E-09	46
WHR	rs1789882	4:100235053	A	0.0160	0.0023	0.20	1.0E-11	65
WHR	rs1800437	19:46181392	C	-0.0209	0.0022	0.24	6.2E-22	128
WHR	rs1800978	9:107665978	C	0.0201	0.0026	0.88	9.2E-15	68
WHR	rs1822489	5:112542527	A	0.0122	0.0019	0.40	1.5E-10	58
WHR	rs1974004	9:95568230	T	0.0173	0.0025	0.14	3.8E-12	57
WHR	rs1997833	20:39690342	T	-0.0122	0.0019	0.73	4.2E-11	47
WHR	rs2047937	16:49864791	T	-0.0106	0.0017	0.45	4.6E-10	45
WHR	rs2061708	1:103417203	C	0.0152	0.0020	0.57	2.1E-14	92
WHR	rs2112347	5:75015242	T	0.0150	0.0017	0.59	8.4E-18	88
WHR	rs213624	1:26201164	A	0.0118	0.0018	0.51	3.3E-11	56
WHR	rs2161097	5:103945178	T	0.0126	0.0018	0.41	2.1E-12	62
WHR	rs2161228	5:88001798	T	0.0192	0.0029	0.11	4.3E-11	57
WHR	rs2167750	4:89730074	T	0.0199	0.0018	0.51	6.5E-29	160
WHR	rs2183825	9:28412375	T	-0.0141	0.0018	0.65	4.4E-15	73
WHR	rs2195086	2:60814466	T	-0.0144	0.0024	0.84	2.8E-09	45
WHR	rs2200155	12:33734935	A	-0.0133	0.0018	0.39	5.1E-13	68
WHR	rs2236519	20:45529571	A	0.0212	0.0018	0.37	1.9E-30	169
WHR	rs2276390	11:111895254	T	-0.0210	0.0019	0.40	5.8E-28	170
WHR	rs2291542	3:49751585	T	0.0168	0.0018	0.27	1.7E-20	90
WHR	rs2294239	22:29449477	A	0.0200	0.0017	0.59	3.2E-31	156
WHR	rs2306589	17:34848874	T	0.0160	0.0017	0.46	2.5E-21	103

WHR	rs2335077	1:107573565	A	-0.0121	0.0019	0.69	8.3E-11	51
WHR	rs2398893	9:96758342	A	0.0157	0.0019	0.71	9.0E-17	83
WHR	rs2448	5:53302354	T	0.0158	0.0020	0.74	4.6E-15	78
WHR	rs2455848	3:15771372	T	0.0128	0.0018	0.29	1.8E-12	55
WHR	rs2503099	6:100610101	A	-0.0214	0.0023	0.85	8.3E-20	97
WHR	rs2513987	11:102977271	A	-0.0138	0.0022	0.25	3.9E-10	57
WHR	rs2526886	14:71359064	T	0.0125	0.0021	0.68	3.6E-09	55
WHR	rs2595004	3:11406721	T	-0.0156	0.0023	0.86	2.1E-11	49
WHR	rs2613505	1:72835410	T	0.0161	0.0022	0.84	1.0E-13	56
WHR	rs2715135	7:50750128	T	0.0109	0.0018	0.37	4.1E-09	44
WHR	rs2725371	8:30854033	A	0.0172	0.0021	0.29	9.9E-16	98
WHR	rs2742690	1:2987268	A	0.0168	0.0025	0.15	9.2E-12	58
WHR	rs28446899	8:72396213	T	0.0362	0.0037	0.06	7.9E-23	121
WHR	rs28451064	21:35593827	A	0.0177	0.0030	0.14	4.2E-09	62
WHR	rs2898885	14:65421274	T	-0.0138	0.0023	0.85	8.5E-10	40
WHR	rs2903995	1:23271504	A	-0.0122	0.0018	0.43	1.6E-11	59
WHR	rs2925979	16:81534790	T	0.0215	0.0018	0.32	1.6E-31	163
WHR	rs2957658	11:10393468	A	-0.0125	0.0020	0.52	1.8E-10	63
WHR	rs3092781	20:45789953	T	-0.0145	0.0017	0.48	1.9E-17	85
WHR	rs3121419	4:3232257	T	-0.0129	0.0019	0.37	1.6E-11	63
WHR	rs313741	1:86273451	A	-0.0120	0.0018	0.51	1.6E-11	58
WHR	rs332105	2:119444229	A	-0.0139	0.0018	0.51	1.1E-14	78
WHR	rs34322	12:12879570	T	0.0104	0.0018	0.42	4.6E-09	43
WHR	rs35169799	11:64031241	T	0.0373	0.0041	0.08	6.3E-20	170
WHR	rs36061954	8:38329650	T	0.0146	0.0020	0.39	3.4E-13	82
WHR	rs372321	7:101721006	A	0.0156	0.0023	0.14	1.7E-11	48
WHR	rs3736485	15:51748610	A	0.0118	0.0017	0.45	3.8E-12	56
WHR	rs3767848	1:214173840	A	-0.0124	0.0021	0.29	2.2E-09	51

WHR	rs3786897	19:33893008	A	-0.0237	0.0017	0.59	1.5E-43	219
WHR	rs3825061	11:118944675	T	0.0142	0.0018	0.32	5.5E-15	71
WHR	rs3891424	2:239365456	A	-0.0278	0.0044	0.06	4.0E-10	69
WHR	rs3903399	1:205041542	T	-0.0149	0.0022	0.77	6.8E-12	63
WHR	rs39312	7:116954785	A	-0.0154	0.0018	0.59	3.5E-18	92
WHR	rs3936510	5:55860866	T	0.0241	0.0021	0.16	1.8E-29	124
WHR	rs399984	2:164877930	C	0.0129	0.0022	0.78	1.9E-09	46
WHR	rs409125	2:165667643	A	-0.0182	0.0018	0.58	2.1E-23	130
WHR	rs4239275	17:79923718	T	0.0118	0.0018	0.40	3.3E-11	54
WHR	rs429358	19:45411941	T	0.0346	0.0027	0.83	4.2E-37	269
WHR	rs4372913	2:114517748	A	-0.0140	0.0022	0.74	8.6E-11	61
WHR	rs4395620	5:106328326	T	0.0106	0.0018	0.51	3.6E-09	45
WHR	rs4454042	5:155824774	T	0.0127	0.0020	0.30	1.4E-10	55
WHR	rs4476935	7:112987650	T	-0.0108	0.0018	0.41	1.5E-09	46
WHR	rs4646342	17:17493272	A	-0.0130	0.0018	0.38	6.6E-13	64
WHR	rs4660808	1:40018509	T	0.0145	0.0021	0.20	7.6E-12	54
WHR	rs4671193	2:67846288	T	-0.0191	0.0018	0.40	4.8E-25	142
WHR	rs4686340	3:9345218	A	0.0123	0.0020	0.26	6.0E-10	47
WHR	rs4714668	6:43101270	C	-0.0117	0.0020	0.56	2.5E-09	54
WHR	rs4727695	7:107614003	A	0.0215	0.0029	0.92	1.8E-13	54
WHR	rs4755720	11:43628749	T	-0.0132	0.0018	0.62	4.0E-13	67
WHR	rs4779526	15:31705683	A	0.0132	0.0021	0.73	2.1E-10	55
WHR	rs4788204	16:29995218	A	0.0169	0.0018	0.42	2.6E-21	112
WHR	rs4851284	2:100894887	T	-0.0151	0.0021	0.72	5.6E-13	74
WHR	rs4894803	3:171800256	A	0.0149	0.0018	0.60	3.7E-16	86
WHR	rs494752	18:46853270	C	-0.0154	0.0019	0.62	2.3E-16	90
WHR	rs4964656	12:108594069	C	-0.0181	0.0020	0.36	2.0E-20	121
WHR	rs536665	11:85322400	A	-0.0171	0.0024	0.86	2.2E-12	57

WHR	rs543874	1:177889480	A	-0.0203	0.0021	0.82	8.1E-22	100
WHR	rs55747707	7:73037366	A	-0.0154	0.0023	0.18	3.4E-11	56
WHR	rs55920843	2:158412701	T	0.0623	0.0092	0.99	1.5E-11	50
WHR	rs579682	11:122014110	T	-0.0133	0.0020	0.74	2.0E-11	54
WHR	rs591939	17:40698075	A	-0.0177	0.0023	0.78	9.1E-15	88
WHR	rs6021889	20:50982870	A	0.0203	0.0019	0.74	2.5E-27	129
WHR	rs605066	6:139829666	T	-0.0192	0.0018	0.54	2.4E-26	148
WHR	rs6130360	20:42010996	A	0.0151	0.0025	0.86	1.1E-09	43
WHR	rs62095889	18:21069068	A	-0.0166	0.0021	0.34	1.6E-15	100
WHR	rs62506196	8:60264465	A	-0.0159	0.0027	0.79	2.7E-09	69
WHR	rs62565259	9:102162570	T	-0.0157	0.0027	0.13	3.7E-09	46
WHR	rs645040	3:135926622	T	0.0156	0.0020	0.81	1.2E-14	61
WHR	rs6474945	9:15670492	T	-0.0101	0.0017	0.49	2.7E-09	41
WHR	rs6545714	2:59307725	A	-0.0139	0.0017	0.61	9.0E-16	75
WHR	rs6548834	3:82704753	A	0.0118	0.0018	0.31	1.6E-10	48
WHR	rs6550597	3:18738940	A	0.0117	0.0020	0.67	3.1E-09	49
WHR	rs6566233	18:53445930	T	-0.0128	0.0020	0.55	7.1E-11	66
WHR	rs6567160	18:57829135	T	-0.0263	0.0020	0.78	2.5E-39	189
WHR	rs6604731	1:224051439	T	-0.0116	0.0019	0.30	2.5E-09	46
WHR	rs664532	13:110932363	T	0.0113	0.0019	0.41	1.7E-09	50
WHR	rs6658723	1:112274162	T	0.0145	0.0020	0.47	2.3E-13	85
WHR	rs6688053	1:163589208	T	0.0123	0.0020	0.48	4.4E-10	61
WHR	rs6688233	1:9335745	T	0.0186	0.0021	0.20	3.0E-19	89
WHR	rs668871	6:160769811	T	-0.0134	0.0020	0.48	7.2E-12	72
WHR	rs6694768	1:114953420	T	0.0121	0.0019	0.68	1.6E-10	51
WHR	rs6699397	1:91212216	A	-0.0118	0.0017	0.51	1.3E-11	56
WHR	rs672341	6:153455994	A	-0.0122	0.0020	0.42	8.0E-10	59
WHR	rs6743060	2:629510	A	0.0266	0.0022	0.82	1.8E-32	167

WHR	rs6749646	2:25193998	A	-0.0210	0.0022	0.84	6.0E-22	95
WHR	rs6795831	3:129341403	A	0.0288	0.0023	0.82	2.1E-37	197
WHR	rs6905288	6:43758873	A	0.0327	0.0017	0.58	2.0E-78	421
WHR	rs6942652	7:120889272	C	-0.0126	0.0018	0.41	2.2E-12	62
WHR	rs7025089	9:134881443	A	-0.0118	0.0019	0.68	4.3E-10	49
WHR	rs7070670	10:61842645	T	-0.0132	0.0021	0.29	3.6E-10	58
WHR	rs7070749	10:63882682	A	0.0126	0.0020	0.55	1.9E-10	63
WHR	rs708437	10:36227656	A	0.0144	0.0024	0.77	1.6E-09	60
WHR	rs711869	2:13073967	A	-0.0171	0.0018	0.58	2.4E-21	115
WHR	rs7138803	12:50247468	A	0.0125	0.0018	0.38	1.1E-12	59
WHR	rs7183908	15:74329193	T	-0.0158	0.0020	0.46	8.3E-16	100
WHR	rs7186893	16:24806420	T	-0.0137	0.0020	0.24	1.2E-11	55
WHR	rs719802	11:113234679	T	0.0114	0.0018	0.37	3.8E-10	49
WHR	rs7198287	16:85258191	T	-0.0138	0.0022	0.20	6.1E-10	49
WHR	rs7206608	16:82872628	C	-0.0127	0.0019	0.66	2.5E-11	58
WHR	rs7213608	17:21279289	T	-0.0171	0.0019	0.65	7.0E-19	107
WHR	rs7217226	17:2136065	T	-0.0137	0.0018	0.65	9.9E-15	69
WHR	rs7222	12:2055266	T	0.0109	0.0018	0.50	1.2E-09	48
WHR	rs72959041	6:127454893	A	0.1260	0.0044	0.06	4.6E-183	1441
WHR	rs7311622	12:98772975	T	-0.0119	0.0018	0.37	3.0E-11	53
WHR	rs733381	22:40669648	A	0.0122	0.0021	0.75	2.8E-09	45
WHR	rs7395513	11:69262756	A	-0.0173	0.0020	0.45	2.5E-18	119
WHR	rs747249	11:130271647	A	0.0111	0.0019	0.35	2.7E-09	45
WHR	rs747601	11:13274553	A	-0.0128	0.0020	0.29	7.3E-11	54
WHR	rs7492628	14:91547136	C	-0.0125	0.0019	0.71	7.9E-11	51
WHR	rs7498665	16:28883241	A	-0.0168	0.0017	0.65	1.1E-22	104
WHR	rs758598	17:59492714	A	0.0123	0.0019	0.31	5.6E-11	52
WHR	rs7591387	2:43756032	T	0.0179	0.0028	0.08	9.3E-11	38

WHR	rs7599312	2:213413231	A	-0.0114	0.0019	0.28	2.4E-09	43
WHR	rs7647305	3:185834290	T	-0.0142	0.0021	0.21	8.3E-12	53
WHR	rs76699125	3:64701535	T	0.0320	0.0041	0.95	5.0E-15	79
WHR	rs7744833	6:20581828	A	0.0125	0.0019	0.73	6.9E-11	50
WHR	rs7797307	7:68686127	C	-0.0245	0.0040	0.04	7.1E-10	38
WHR	rs780159	10:80907147	A	-0.0133	0.0017	0.42	2.9E-14	70
WHR	rs7823561	8:25641764	A	0.0159	0.0019	0.64	3.3E-17	94
WHR	rs789351	4:145868370	T	0.0123	0.0017	0.38	7.6E-13	57
WHR	rs7907173	10:5648787	A	-0.0109	0.0018	0.46	9.4E-10	48
WHR	rs793456	3:99525631	A	-0.0106	0.0018	0.58	3.6E-09	44
WHR	rs797486	13:51221618	A	0.0316	0.0026	0.87	1.1E-34	179
WHR	rs8024294	15:94023132	A	0.0177	0.0028	0.12	3.6E-10	52
WHR	rs8043060	15:67661784	A	-0.0166	0.0020	0.25	8.9E-17	84
WHR	rs8060576	16:4908956	T	0.0161	0.0027	0.11	1.5E-09	41
WHR	rs8070737	17:3981066	T	0.0160	0.0023	0.19	7.4E-12	63
WHR	rs8071778	17:46080233	C	-0.0160	0.0025	0.15	6.6E-11	52
WHR	rs8079062	17:74255029	A	-0.0234	0.0033	0.94	1.7E-12	51
WHR	rs809955	4:140874760	A	-0.0155	0.0020	0.35	2.5E-14	89
WHR	rs8103017	19:55999142	C	-0.0165	0.0022	0.72	2.2E-14	89
WHR	rs8141715	22:47214749	T	-0.0136	0.0020	0.68	1.5E-11	65
WHR	rs858516	17:7537098	T	-0.0119	0.0020	0.58	1.6E-09	56
WHR	rs861029	4:56245637	T	-0.0135	0.0020	0.70	4.4E-11	62
WHR	rs863750	12:124505444	T	0.0259	0.0017	0.58	6.2E-51	263
WHR	rs889398	16:69556715	T	-0.0171	0.0017	0.41	2.0E-23	114
WHR	rs901630	6:98539519	T	-0.0119	0.0017	0.35	6.8E-12	52
WHR	rs905938	1:154991389	T	0.0126	0.0019	0.77	7.8E-11	45
WHR	rs910382	20:51699189	A	-0.0169	0.0018	0.45	2.4E-21	114
WHR	rs929641	2:58792377	A	0.0127	0.0017	0.60	8.0E-14	62

WHR	rs9296938	6:14573063	A	-0.0145	0.0020	0.27	1.5E-12	67
WHR	rs9362083	6:85396119	A	-0.0117	0.0018	0.59	7.8E-11	53
WHR	rs936226	15:75069282	T	0.0114	0.0019	0.70	2.0E-09	44
WHR	rs9369425	6:43810974	A	0.0195	0.0019	0.69	1.1E-23	131
WHR	rs9370243	6:53789830	T	0.0198	0.0032	0.10	1.1E-09	56
WHR	rs946106	1:49928489	T	-0.0170	0.0020	0.76	2.7E-17	84
WHR	rs950732	10:94114633	T	0.0120	0.0018	0.51	2.4E-11	58
WHR	rs9515201	13:111040798	A	-0.0121	0.0018	0.29	6.6E-11	49
WHR	rs9630986	2:181607751	C	0.0122	0.0019	0.71	1.0E-10	50
WHR	rs9644033	8:23610639	A	0.0188	0.0021	0.78	2.1E-19	98
WHR	rs9659380	1:98423149	A	-0.0179	0.0024	0.78	5.2E-14	88
WHR	rs979012	20:6623374	T	0.0116	0.0018	0.33	7.9E-11	48
WHR	rs987237	6:50803050	A	-0.0200	0.0022	0.80	1.0E-19	105
WHR	rs9878908	3:12302462	T	-0.0193	0.0021	0.79	3.4E-20	98
WHR	rs9923544	16:53801985	T	0.0389	0.0017	0.42	1.2E-113	596
WHR	rs9942009	3:89121921	T	0.0126	0.0018	0.40	3.0E-12	62
WHR	rs9976841	21:39484323	A	-0.0129	0.0018	0.49	2.1E-12	67
WHR	rs998732	19:19378671	A	0.0165	0.0023	0.88	1.0E-12	48
WHR	rs9988	17:73230856	T	0.0152	0.0024	0.86	1.9E-10	45
WC	rs10132280	14:25928179	C	0.0220	0.0037	0.68	2.2E-09	48
WC	rs10767658	11:27672252	C	0.0312	0.0037	0.33	3.3E-17	97
WC	rs10840100	11:8669437	G	0.0203	0.0035	0.63	5.4E-09	43
WC	rs10938397	4:45182527	G	0.0316	0.0035	0.45	6.1E-20	111
WC	rs10968576	9:28414339	G	0.0246	0.0036	0.34	1.2E-11	61
WC	rs11165623	1:96893000	A	0.0198	0.0034	0.44	5.2E-09	43
WC	rs12429545	13:54102206	A	0.0309	0.0052	0.14	2.5E-09	52
WC	rs12446632	16:19935389	G	0.0361	0.0050	0.88	5.2E-13	64
WC	rs12885454	14:29736838	C	0.0197	0.0035	0.65	2.6E-08	40

WC	rs1516725	3:185824004	C	0.0310	0.0051	0.87	1.7E-09	48
WC	rs1549293	16:31141993	C	0.0201	0.0035	0.63	7.3E-09	42
WC	rs1558902	16:53803574	A	0.0739	0.0035	0.40	3.7E-101	592
WC	rs16894959	6:34825662	C	0.0262	0.0048	0.18	3.4E-08	46
WC	rs16996700	20:50981945	T	0.0226	0.0037	0.76	1.5E-09	42
WC	rs2033529	6:40348653	G	0.0209	0.0037	0.31	1.7E-08	42
WC	rs2075650	19:45395619	A	0.0307	0.0050	0.84	8.9E-10	57
WC	rs2112347	5:75015242	T	0.0254	0.0035	0.59	3.7E-10	70
WC	rs2176040	2:227092802	A	0.0151	0.0035	0.36	1.5E-05	24
WC	rs2287019	19:46202172	C	0.0351	0.0046	0.79	1.7E-14	93
WC	rs2293576	11:47434986	G	0.0222	0.0036	0.70	9.4E-10	46
WC	rs2325036	3:85819412	A	0.0232	0.0035	0.56	2.1E-11	60
WC	rs2489623	6:127455821	C	0.0187	0.0034	0.53	3.4E-08	39
WC	rs2531992	16:4021734	G	0.0282	0.0048	0.81	3.0E-09	54
WC	rs2650492	16:28333411	A	0.0256	0.0038	0.27	2.5E-11	57
WC	rs2820292	1:201784287	C	0.0189	0.0034	0.54	2.4E-08	40
WC	rs3127553	1:49438005	G	0.0226	0.0035	0.38	1.6E-10	54
WC	rs3810291	19:47569003	A	0.0259	0.0040	0.62	1.7E-10	71
WC	rs3849570	3:81792112	A	0.0214	0.0038	0.39	2.2E-08	49
WC	rs4130548	1:78463868	C	0.0220	0.0035	0.33	3.4E-10	48
WC	rs4776970	15:68080886	A	0.0196	0.0035	0.64	2.3E-08	40
WC	rs6163	10:104596924	A	0.0191	0.0035	0.39	3.7E-08	39
WC	rs633715	1:177852580	C	0.0431	0.0043	0.21	3.3E-23	141
WC	rs6545714	2:59307725	G	0.0220	0.0035	0.40	1.9E-10	52
WC	rs6567160	18:57829135	C	0.0483	0.0040	0.22	2.6E-33	178
WC	rs6755502	2:635721	C	0.0512	0.0045	0.82	2.0E-30	171
WC	rs7138803	12:50247468	A	0.0282	0.0035	0.38	1.6E-15	84
WC	rs7144011	14:79940383	T	0.0330	0.0041	0.23	9.4E-16	86

WC	rs7239883	18:40147671	G	0.0207	0.0035	0.42	2.3E-09	47
WC	rs7498665	16:28883241	G	0.0338	0.0035	0.35	1.4E-22	117
WC	rs7531118	1:72837239	C	0.0268	0.0035	0.55	1.5E-14	80
WC	rs7550711	1:110082886	T	0.0577	0.0098	0.05	3.4E-09	71
WC	rs7903146	10:114758349	C	0.0219	0.0037	0.77	3.9E-09	39
WC	rs806794	6:26200677	A	0.0223	0.0037	0.62	2.1E-09	53
WC	rs929641	2:58792377	A	0.0207	0.0034	0.60	1.2E-09	46
WC	rs9400239	6:108977663	C	0.0244	0.0036	0.64	1.9E-11	62
WC	rs943005	6:50865820	T	0.0388	0.0044	0.19	7.2E-19	106
VAT	rs10057588	5:43160167	G	-0.0145	0.0026	0.41	3.8E-08	33
VAT	rs10182458	2:25150641	G	0.0261	0.0025	0.44	5.9E-26	110
VAT	rs10187101	2:50742227	T	-0.0172	0.0026	0.41	2.7E-11	46
VAT	rs10423928	19:46182304	A	-0.0328	0.0031	0.24	1.0E-25	127
VAT	rs10510025	10:118650996	T	0.0164	0.0029	0.26	1.4E-08	34
VAT	rs10740991	10:22058137	G	0.0257	0.0028	0.28	1.3E-20	86
VAT	rs10756714	9:15885041	G	-0.0197	0.0025	0.48	3.4E-15	63
VAT	rs10773302	12:123043145	G	-0.0172	0.0028	0.24	7.7E-10	35
VAT	rs10789334	1:72647903	A	-0.0185	0.0030	0.19	4.2E-10	33
VAT	rs10896012	11:65278461	C	0.0224	0.0030	0.14	9.5E-14	39
VAT	rs10938398	4:45186139	A	0.0284	0.0025	0.45	1.0E-29	129
VAT	rs11030112	11:27705188	A	0.0313	0.0026	0.32	2.7E-32	138
VAT	rs11126734	2:27035978	A	-0.0154	0.0025	0.41	8.0E-10	37
VAT	rs111363146	7:44801682	C	0.0198	0.0036	0.09	3.6E-08	21
VAT	rs11150745	17:78757626	G	-0.0194	0.0027	0.29	3.1E-13	51
VAT	rs11161044	14:29675957	G	-0.0173	0.0032	0.25	4.5E-08	36
VAT	rs11173521	12:60951398	T	0.0141	0.0025	0.45	1.9E-08	32
VAT	rs112108364	13:86490590	G	0.0173	0.0028	0.37	3.3E-10	46
VAT	rs113866544	17:46270606	C	0.0366	0.0049	0.10	1.2E-13	78

VAT	rs114067739	1:46260329	A	-0.0366	0.0057	0.06	1.7E-10	52
VAT	rs11679338	2:181606895	C	-0.0172	0.0026	0.29	4.2E-11	40
VAT	rs117151227	18:58121144	C	-0.0618	0.0076	0.03	5.0E-16	64
VAT	rs117176448	8:27261138	G	0.0248	0.0042	0.08	3.7E-09	31
VAT	rs11776713	8:30861607	C	-0.0151	0.0025	0.49	1.0E-09	37
VAT	rs11880870	19:18830704	G	-0.0185	0.0025	0.52	8.1E-14	56
VAT	rs11896591	2:57239889	G	0.0137	0.0025	0.48	3.7E-08	30
VAT	rs11917587	3:141207575	A	0.0144	0.0025	0.42	8.9E-09	33
VAT	rs12001634	9:94186527	A	-0.0161	0.0026	0.31	7.9E-10	36
VAT	rs12101386	15:92571283	T	-0.0164	0.0030	0.24	5.0E-08	32
VAT	rs12103006	16:24726237	A	-0.0166	0.0025	0.50	3.3E-11	45
VAT	rs12200046	6:154315310	T	0.0218	0.0037	0.15	5.9E-09	40
VAT	rs1225060	3:131637173	A	0.0232	0.0028	0.26	7.9E-17	67
VAT	rs1229984	4:100239319	T	-0.0498	0.0084	0.11	2.8E-09	156
VAT	rs12335914	9:92209151	C	0.0160	0.0025	0.48	1.2E-10	42
VAT	rs12409875	1:2424765	A	-0.0143	0.0025	0.45	7.2E-09	33
VAT	rs12435171	14:101155220	G	0.0163	0.0027	0.38	3.0E-09	40
VAT	rs12459368	19:18459377	G	-0.0199	0.0028	0.25	1.2E-12	48
VAT	rs12477088	2:67841326	C	-0.0203	0.0025	0.41	6.2E-16	65
VAT	rs12632423	3:52041566	A	-0.0227	0.0041	0.12	2.1E-08	35
VAT	rs12739999	1:32207990	A	0.0217	0.0033	0.21	5.5E-11	51
VAT	rs13017207	2:100841235	A	-0.0196	0.0025	0.39	9.2E-15	60
VAT	rs13062093	3:35667057	G	0.0197	0.0026	0.35	1.5E-14	58
VAT	rs13075615	3:136505832	T	-0.0217	0.0035	0.11	4.0E-10	30
VAT	rs13097150	3:62433911	T	0.0154	0.0026	0.36	2.0E-09	35
VAT	rs13135092	4:103198082	G	0.0326	0.0045	0.05	7.3E-13	31
VAT	rs13192865	6:97929355	A	-0.0182	0.0028	0.26	8.8E-11	41
VAT	rs13263674	8:14238841	G	0.0171	0.0027	0.25	4.7E-10	35

VAT	rs13337177	16:2175323	T	-0.0228	0.0032	0.18	2.0E-12	49
VAT	rs13393304	2:637830	A	-0.0445	0.0033	0.18	6.1E-42	186
VAT	rs1446585	2:136407479	G	-0.0169	0.0030	0.36	1.2E-08	43
VAT	rs145350287	12:120907309	A	-0.0424	0.0063	0.04	1.9E-11	40
VAT	rs1454687	3:94038085	C	0.0230	0.0025	0.46	1.5E-20	86
VAT	rs1474518	8:116825690	C	-0.0181	0.0029	0.25	6.6E-10	40
VAT	rs148168215	9:30835804	T	-0.0532	0.0094	0.02	1.5E-08	36
VAT	rs1559677	15:47738063	G	0.0149	0.0025	0.42	4.2E-09	35
VAT	rs1591726	1:49963473	T	0.0223	0.0027	0.37	4.1E-17	76
VAT	rs1652376	18:21109466	T	-0.0209	0.0025	0.48	4.2E-17	71
VAT	rs17239176	10:107744042	C	-0.0166	0.0030	0.24	2.9E-08	33
VAT	rs1724557	4:137094048	C	0.0156	0.0025	0.48	6.9E-10	39
VAT	rs17589357	15:35927468	C	-0.0199	0.0035	0.09	1.1E-08	22
VAT	rs1762509	1:107592959	A	0.0157	0.0026	0.31	2.2E-09	35
VAT	rs17682873	17:52940772	T	0.0196	0.0035	0.12	3.1E-08	27
VAT	rs17770336	9:28414625	T	0.0247	0.0026	0.34	7.9E-21	90
VAT	rs1834144	18:40744790	A	-0.0181	0.0026	0.42	1.7E-12	52
VAT	rs1928496	13:31012904	C	-0.0211	0.0028	0.30	9.3E-14	60
VAT	rs2020942	17:28546914	T	0.0152	0.0025	0.42	2.3E-09	36
VAT	rs2102278	4:52818664	G	0.0157	0.0027	0.34	3.5E-09	36
VAT	rs215628	7:32360096	C	0.0158	0.0026	0.35	6.5E-10	37
VAT	rs2172131	10:133978962	T	0.0169	0.0025	0.46	1.9E-11	46
VAT	rs217669	14:62360075	C	0.0175	0.0028	0.25	3.3E-10	37
VAT	rs2253310	6:108888593	C	-0.0209	0.0026	0.42	3.2E-16	70
VAT	rs2285640	17:34951204	G	0.0200	0.0025	0.45	7.6E-16	64
VAT	rs2304608	5:87962298	A	0.0299	0.0034	0.15	1.9E-18	76
VAT	rs2307111	5:75003678	C	-0.0260	0.0025	0.43	1.1E-24	108
VAT	rs2448916	8:95308350	A	-0.0140	0.0026	0.45	3.6E-08	32

VAT	rs245775	5:170532105	A	-0.0196	0.0028	0.31	2.2E-12	53
VAT	rs2472297	15:75027880	T	0.0166	0.0028	0.24	3.1E-09	32
VAT	rs247975	3:173107443	T	-0.0144	0.0025	0.44	7.5E-09	33
VAT	rs2481665	1:62594677	C	-0.0178	0.0025	0.37	7.8E-13	48
VAT	rs2499468	6:51809081	C	-0.0156	0.0026	0.31	2.0E-09	34
VAT	rs2537621	7:5557255	C	0.0146	0.0026	0.38	1.3E-08	33
VAT	rs254024	5:103944020	T	0.0156	0.0025	0.41	4.2E-10	38
VAT	rs264932	2:104288396	A	0.0139	0.0025	0.40	3.7E-08	30
VAT	rs2667761	15:77883130	C	-0.0159	0.0026	0.41	7.3E-10	40
VAT	rs2678204	1:201800511	G	0.0231	0.0026	0.30	1.2E-18	73
VAT	rs2730806	12:41866904	T	0.0165	0.0025	0.47	3.4E-11	44
VAT	rs2744973	6:34580221	T	0.0214	0.0027	0.32	2.0E-15	65
VAT	rs2799465	9:126544609	C	0.0213	0.0037	0.16	6.0E-09	40
VAT	rs2804477	10:33720175	A	0.0211	0.0036	0.13	5.5E-09	33
VAT	rs2926614	8:76301610	T	-0.0224	0.0032	0.20	4.2E-12	53
VAT	rs2926864	5:153213196	A	0.0175	0.0026	0.31	2.7E-11	42
VAT	rs2962082	16:62816628	A	-0.0154	0.0025	0.46	6.7E-10	38
VAT	rs329124	5:133865452	G	-0.0138	0.0025	0.42	4.2E-08	30
VAT	rs34431565	6:31782180	T	-0.0340	0.0059	0.05	9.1E-09	32
VAT	rs34811474	4:25408838	A	-0.0191	0.0029	0.22	7.0E-11	41
VAT	rs35060985	11:43693110	A	0.0234	0.0027	0.31	2.5E-18	76
VAT	rs35697587	14:47298505	G	0.0167	0.0025	0.46	1.6E-11	45
VAT	rs35972789	10:75519691	A	-0.0396	0.0065	0.03	1.0E-09	28
VAT	rs362307	4:3241845	T	0.0287	0.0048	0.07	2.0E-09	35
VAT	rs3759094	12:56497903	T	-0.0152	0.0026	0.33	7.1E-09	33
VAT	rs3774063	3:9511736	T	0.0238	0.0041	0.08	6.6E-09	27
VAT	rs3784692	15:67988133	C	-0.0244	0.0025	0.39	5.7E-22	92
VAT	rs3787075	20:25186502	G	0.0178	0.0026	0.34	1.3E-11	46

VAT	rs3791687	2:212258767	T	0.0191	0.0030	0.23	1.2E-10	42
VAT	rs3803253	13:99117149	A	-0.0163	0.0027	0.26	2.4E-09	34
VAT	rs3826408	17:7101292	T	0.0143	0.0025	0.49	8.0E-09	33
VAT	rs3843540	7:99126640	C	-0.0256	0.0035	0.21	2.2E-13	72
VAT	rs3943933	5:63020327	A	0.0166	0.0025	0.55	2.2E-11	44
VAT	rs40067	5:107439012	A	-0.0245	0.0033	0.21	1.6E-13	64
VAT	rs4073582	11:66050712	A	-0.0175	0.0026	0.32	1.2E-11	43
VAT	rs4148866	12:123425575	T	0.0153	0.0025	0.41	1.3E-09	37
VAT	rs4239060	17:1844519	A	-0.0256	0.0032	0.21	9.0E-16	70
VAT	rs429358	19:45411941	C	-0.0304	0.0034	0.17	4.6E-19	84
VAT	rs4399192	1:98376502	G	0.0178	0.0030	0.29	1.8E-09	42
VAT	rs4402589	16:29954654	T	-0.0263	0.0025	0.50	6.2E-26	112
VAT	rs4419475	4:96150044	T	0.0139	0.0025	0.46	3.7E-08	31
VAT	rs4482463	2:205375909	C	0.0362	0.0047	0.09	1.7E-14	73
VAT	rs4500930	2:228985505	T	0.0171	0.0026	0.33	5.8E-11	42
VAT	rs4558773	3:82710125	A	0.0172	0.0026	0.33	2.2E-11	42
VAT	rs4562625	1:209218583	C	0.0150	0.0025	0.40	3.8E-09	35
VAT	rs4807179	19:1956035	G	-0.0146	0.0026	0.40	1.5E-08	33
VAT	rs4808762	19:18326222	C	0.0289	0.0027	0.26	3.6E-26	104
VAT	rs4809221	20:62372706	G	-0.0146	0.0026	0.29	3.2E-08	28
VAT	rs4842920	15:84539424	T	-0.0154	0.0028	0.30	2.4E-08	32
VAT	rs4872376	8:25759022	C	-0.0138	0.0025	0.44	2.7E-08	31
VAT	rs4929923	11:8639200	T	-0.0180	0.0026	0.37	3.8E-12	49
VAT	rs496072	4:171055797	T	0.0139	0.0025	0.47	3.4E-08	31
VAT	rs538656	18:57850422	T	0.0453	0.0029	0.22	1.8E-54	229
VAT	rs539515	1:177889025	C	0.0380	0.0031	0.18	1.4E-35	140
VAT	rs55726687	12:991306	A	0.0227	0.0030	0.21	8.1E-14	55
VAT	rs55742087	3:185830488	T	-0.0233	0.0032	0.17	3.6E-13	49

VAT	rs55769038	11:13331808	G	-0.0159	0.0025	0.48	3.4E-10	41
VAT	rs55911231	13:96983940	T	0.0148	0.0025	0.48	4.3E-09	36
VAT	rs56094641	16:53806453	G	0.0647	0.0025	0.40	3.8E-145	656
VAT	rs56356382	19:4064057	C	-0.0217	0.0032	0.16	6.5E-12	42
VAT	rs57241669	4:175246482	G	-0.0257	0.0047	0.09	3.9E-08	35
VAT	rs577525	10:99769388	T	-0.0177	0.0025	0.49	1.5E-12	51
VAT	rs58120873	2:47313562	A	-0.0248	0.0045	0.08	2.7E-08	30
VAT	rs59066241	12:97925364	G	0.0212	0.0039	0.16	4.4E-08	40
VAT	rs60377014	3:88187052	T	-0.0203	0.0035	0.16	4.5E-09	36
VAT	rs6096886	20:50951298	G	-0.0284	0.0032	0.17	2.4E-19	75
VAT	rs61537964	1:115585759	G	-0.0231	0.0040	0.12	8.1E-09	38
VAT	rs61813293	1:156019075	T	0.0237	0.0035	0.15	1.9E-11	45
VAT	rs61903695	11:89922417	G	0.0168	0.0028	0.24	3.0E-09	34
VAT	rs61910767	11:134515899	T	-0.0229	0.0033	0.14	7.0E-12	40
VAT	rs62024481	15:99238661	T	-0.0177	0.0031	0.18	1.1E-08	30
VAT	rs62084234	17:65847060	G	0.0255	0.0031	0.23	3.7E-16	75
VAT	rs62104473	19:30289779	T	0.0192	0.0026	0.33	3.5E-13	53
VAT	rs62183012	2:159607760	C	-0.0162	0.0027	0.29	3.0E-09	35
VAT	rs62190394	2:230624929	T	0.0219	0.0027	0.32	2.0E-16	68
VAT	rs62261725	3:85898626	G	-0.0214	0.0026	0.35	5.5E-16	68
VAT	rs62262093	3:49960388	T	-0.0299	0.0025	0.59	1.3E-33	141
VAT	rs62413414	6:80310375	T	0.0198	0.0034	0.14	8.8E-09	31
VAT	rs62473743	7:76610299	A	0.0198	0.0034	0.22	6.8E-09	44
VAT	rs62477685	7:75101707	T	-0.0195	0.0025	0.48	7.8E-15	62
VAT	rs6433243	2:171605495	T	0.0162	0.0026	0.33	4.6E-10	38
VAT	rs6536575	4:162091639	T	-0.0141	0.0025	0.45	1.5E-08	32
VAT	rs653958	1:96884006	G	0.0187	0.0026	0.40	3.3E-13	55
VAT	rs66679256	4:18351898	T	0.0185	0.0025	0.40	1.3E-13	53

VAT	rs669696	16:69626136	A	-0.0242	0.0025	0.41	6.2E-22	93
VAT	rs6739755	2:59330227	A	0.0227	0.0025	0.40	4.0E-19	80
VAT	rs67463976	7:70045443	C	0.0163	0.0025	0.42	9.1E-11	42
VAT	rs684214	17:40696915	T	0.0179	0.0028	0.25	8.4E-11	39
VAT	rs7021721	9:111963641	C	-0.0154	0.0027	0.29	1.7E-08	32
VAT	rs7035637	9:16739403	A	0.0187	0.0028	0.27	3.6E-11	44
VAT	rs704061	12:89771903	C	0.0165	0.0025	0.41	3.9E-11	43
VAT	rs7132908	12:50263148	A	0.0255	0.0025	0.38	1.2E-23	100
VAT	rs7156625	14:79942647	A	0.0269	0.0030	0.23	2.0E-19	83
VAT	rs7165759	15:80988600	A	-0.0175	0.0027	0.27	1.0E-10	40
VAT	rs71658797	1:77967507	A	0.0347	0.0038	0.12	4.8E-20	82
VAT	rs719802	11:113234679	T	0.0174	0.0025	0.37	7.8E-12	46
VAT	rs72663503	1:39969059	T	0.0209	0.0030	0.22	1.7E-12	49
VAT	rs72892910	6:50816887	T	0.0362	0.0033	0.20	7.2E-28	135
VAT	rs72995085	6:143193971	C	-0.0215	0.0032	0.20	3.8E-11	48
VAT	rs73033486	7:2908277	A	0.0207	0.0037	0.11	3.0E-08	27
VAT	rs7308188	12:103701537	C	-0.0187	0.0029	0.29	5.7E-11	46
VAT	rs73213484	4:28489339	T	-0.0211	0.0036	0.16	4.1E-09	38
VAT	rs7324067	13:65484906	T	-0.0165	0.0029	0.27	1.2E-08	35
VAT	rs74934567	3:61239698	G	-0.0220	0.0034	0.16	1.2E-10	42
VAT	rs7498665	16:28883241	G	0.0269	0.0025	0.35	1.8E-26	107
VAT	rs754635	3:42305131	C	-0.0230	0.0039	0.13	3.3E-09	39
VAT	rs7550711	1:110082886	T	0.0615	0.0079	0.05	5.2E-15	117
VAT	rs7586854	2:188075837	T	-0.0145	0.0025	0.51	5.6E-09	34
VAT	rs76040172	21:46488959	A	-0.0451	0.0055	0.07	2.6E-16	91
VAT	rs76111507	2:455477	T	-0.0754	0.0066	0.02	1.7E-30	77
VAT	rs76327888	14:103380403	T	0.0230	0.0033	0.21	2.9E-12	57
VAT	rs7649970	3:12392272	T	0.0255	0.0038	0.15	1.8E-11	55

VAT	rs7654647	4:80810453	T	0.0155	0.0025	0.35	6.9E-10	36
VAT	rs7724430	5:153546427	A	0.0139	0.0025	0.44	2.7E-08	31
VAT	rs7773094	6:129440026	C	-0.0177	0.0031	0.20	1.2E-08	33
VAT	rs778094	2:147903802	G	0.0150	0.0025	0.49	2.6E-09	36
VAT	rs7788950	7:71580718	A	-0.0175	0.0032	0.21	5.0E-08	33
VAT	rs7822494	8:87476439	C	-0.0162	0.0025	0.41	6.5E-11	42
VAT	rs7845090	8:73449940	G	0.0201	0.0028	0.29	3.2E-13	54
VAT	rs7849553	9:73832360	C	0.0141	0.0025	0.48	1.4E-08	32
VAT	rs7864091	9:96346188	A	0.0186	0.0034	0.20	2.9E-08	36
VAT	rs78719460	12:133395038	A	0.0159	0.0027	0.26	2.8E-09	32
VAT	rs7893571	10:16750129	G	-0.0179	0.0026	0.31	1.2E-11	45
VAT	rs7942037	11:30430332	C	-0.0169	0.0026	0.32	5.6E-11	40
VAT	rs7982447	13:54453811	C	0.0208	0.0031	0.21	1.6E-11	46
VAT	rs8015400	14:25930988	C	-0.0199	0.0027	0.36	5.2E-14	59
VAT	rs8074454	17:3981148	C	0.0174	0.0026	0.30	3.8E-11	42
VAT	rs809955	4:140874760	A	-0.0158	0.0026	0.35	7.5E-10	37
VAT	rs8103728	19:33900257	C	-0.0152	0.0026	0.36	8.0E-09	35
VAT	rs879620	16:4015729	C	-0.0206	0.0026	0.43	9.0E-16	68
VAT	rs916289	13:33366248	T	-0.0138	0.0025	0.43	3.3E-08	30
VAT	rs9277979	6:33294098	T	0.0231	0.0032	0.14	8.8E-13	42
VAT	rs9304665	19:47602577	T	-0.0165	0.0029	0.29	1.7E-08	37
VAT	rs9320823	6:98429337	T	-0.0232	0.0025	0.34	4.9E-20	79
VAT	rs9358912	6:26211146	T	-0.0246	0.0028	0.32	1.2E-18	86
VAT	rs9471333	6:40362023	C	0.0244	0.0025	0.53	1.1E-22	96
VAT	rs9512696	13:28012527	A	-0.0144	0.0026	0.36	4.0E-08	31
VAT	rs9522285	13:112230701	A	0.0187	0.0025	0.45	9.6E-14	56
VAT	rs9569934	13:59257944	T	-0.0188	0.0032	0.18	4.0E-09	34
VAT	rs9641499	7:112984493	A	-0.0178	0.0025	0.41	1.2E-12	50

VAT	rs9832402	3:173742542	G	-0.0170	0.0029	0.26	3.0E-09	36
VAT	rs9843340	3:156317073	C	-0.0221	0.0035	0.15	1.7E-10	40
VAT	rs9925945	16:393564	C	-0.0175	0.0028	0.22	7.0E-10	34
VAT	rs9989141	14:94006257	C	-0.0201	0.0026	0.39	6.8E-15	62
Smoking Initiation	rs10042827	5:170299916	T	-0.0167	0.0027	0.33	9.4E-10	153
Smoking Initiation	rs1004787	2:45159091	G	-0.0284	0.0026	0.39	1.1E-28	472
Smoking Initiation	rs10060196	5:106455988	C	-0.0183	0.0026	0.39	1.3E-12	197
Smoking Initiation	rs1008078	1:91189731	C	-0.0228	0.0026	0.50	1.6E-18	321
Smoking Initiation	rs1022376	2:22067213	T	0.0147	0.0026	0.49	1.7E-08	134
Smoking Initiation	rs10233018	7:117523709	A	-0.0246	0.0025	0.43	4.8E-22	367
Smoking Initiation	rs10279261	7:133589846	G	0.0189	0.0026	0.37	6.1E-13	204
Smoking Initiation	rs1030015	7:78139581	G	-0.0143	0.0026	0.49	2.2E-08	126
Smoking Initiation	rs10446419	3:25725501	A	0.0196	0.0031	0.79	5.1E-10	158
Smoking Initiation	rs10490159	2:51341259	C	-0.0172	0.0026	0.62	3.9E-11	173
Smoking Initiation	rs1050847	16:87443734	C	0.0148	0.0026	0.50	7.4E-09	135
Smoking Initiation	rs10698713	6:158882320	G	0.0335	0.0056	0.98	2.4E-09	48
Smoking Initiation	rs10789369	1:73824909	A	0.0234	0.0026	0.37	3.4E-19	317
Smoking Initiation	rs10805858	5:88873832	A	-0.0181	0.0027	0.67	1.9E-11	178
Smoking Initiation	rs10853981	19:4965064	G	-0.0148	0.0027	0.71	4.9E-08	111
Smoking Initiation	rs10858334	9:137989785	C	-0.0229	0.0038	0.89	1.2E-09	122
Smoking Initiation	rs10873871	1:76689019	A	-0.0175	0.0031	0.80	2.8E-08	119
Smoking Initiation	rs10885480	10:115378364	T	0.0187	0.0028	0.71	3.8E-11	178
Smoking Initiation	rs10905461	10:8803551	T	0.0164	0.0029	0.25	2.4E-08	123
Smoking Initiation	rs10914684	1:33795572	G	0.0158	0.0027	0.68	6.3E-09	135
Smoking Initiation	rs10935779	3:149543102	C	0.0143	0.0026	0.57	2.9E-08	124
Smoking Initiation	rs10945141	6:69470709	G	-0.0181	0.0029	0.74	3.6E-10	154
Smoking Initiation	rs10953957	7:121954709	G	-0.0144	0.0026	0.60	3.7E-08	123
Smoking Initiation	rs10966092	9:23831658	T	0.0205	0.0029	0.70	1.1E-12	218

Smoking Initiation	rs10969352	9:29747488	T	-0.0143	0.0025	0.54	1.8E-08	126
Smoking Initiation	rs11057005	12:16748721	A	0.0157	0.0026	0.50	9.1E-10	152
Smoking Initiation	rs1106363	11:131966264	C	-0.0174	0.0027	0.61	9.2E-11	178
Smoking Initiation	rs11076962	16:5811367	T	-0.0183	0.0028	0.73	1.2E-10	162
Smoking Initiation	rs1108130	13:100648356	T	-0.0239	0.0031	0.75	1.6E-14	267
Smoking Initiation	rs1109480	12:121083279	G	0.0167	0.0026	0.65	1.8E-10	156
Smoking Initiation	rs11128203	3:71064431	T	-0.0204	0.0026	0.52	1.3E-15	256
Smoking Initiation	rs11162019	1:87913176	C	0.0155	0.0026	0.66	5.1E-09	133
Smoking Initiation	rs1116690	4:143510148	A	-0.0163	0.0029	0.26	2.2E-08	127
Smoking Initiation	rs11192347	10:106929313	G	0.0265	0.0043	0.92	6.2E-10	130
Smoking Initiation	rs11258417	10:13533053	C	0.0145	0.0026	0.67	2.7E-08	114
Smoking Initiation	rs1126757	19:55879872	C	-0.0142	0.0026	0.47	2.9E-08	123
Smoking Initiation	rs112725451	4:68017710	C	-0.0261	0.0034	0.82	1.6E-14	249
Smoking Initiation	rs113230003	19:18460956	G	0.0189	0.0029	0.76	1.1E-10	159
Smoking Initiation	rs1139897	16:720986	G	0.0241	0.0030	0.68	1.8E-15	313
Smoking Initiation	rs114976176	2:264621	A	0.0155	0.0027	0.69	6.0E-09	127
Smoking Initiation	rs1150668	6:28129789	T	0.0185	0.0026	0.54	8.5E-13	210
Smoking Initiation	rs11594623	10:103960351	T	-0.0274	0.0030	0.78	7.5E-20	319
Smoking Initiation	rs1160685	4:94052854	C	-0.0153	0.0026	0.58	2.3E-09	140
Smoking Initiation	rs11611651	12:133380790	G	-0.0271	0.0045	0.94	2.1E-09	106
Smoking Initiation	rs11642231	16:89608702	G	0.0156	0.0026	0.59	3.4E-09	145
Smoking Initiation	rs11651955	17:16235462	G	0.0140	0.0025	0.53	3.7E-08	121
Smoking Initiation	rs11692435	2:98275354	G	-0.0251	0.0046	0.79	4.5E-08	254
Smoking Initiation	rs11713899	3:2365026	A	-0.0187	0.0034	0.84	3.1E-08	115
Smoking Initiation	rs1173461	5:157707571	C	-0.0166	0.0027	0.68	9.5E-10	147
Smoking Initiation	rs117657830	16:75766873	A	0.0378	0.0064	0.97	3.2E-09	93
Smoking Initiation	rs11766326	7:111100585	T	0.0175	0.0026	0.49	1.8E-11	190
Smoking Initiation	rs11768481	7:96629103	C	0.0186	0.0027	0.60	5.2E-12	204

Smoking Initiation	rs117734003	19:51129745	G	-0.0303	0.0051	0.92	2.6E-09	159
Smoking Initiation	rs11783093	8:27425349	C	0.0471	0.0035	0.87	2.1E-41	631
Smoking Initiation	rs11791671	9:3398679	C	-0.0279	0.0051	0.95	4.2E-08	85
Smoking Initiation	rs118202	6:111658371	G	0.0367	0.0033	0.27	1.9E-29	660
Smoking Initiation	rs11872397	18:72535282	G	0.0171	0.0029	0.77	5.2E-09	127
Smoking Initiation	rs1187820	3:173072584	C	0.0143	0.0026	0.60	2.7E-08	120
Smoking Initiation	rs11956866	5:161018271	T	0.0148	0.0026	0.49	7.8E-09	136
Smoking Initiation	rs12022778	1:50603995	A	-0.0268	0.0032	0.79	3.2E-17	292
Smoking Initiation	rs12027999	1:154206358	T	0.0244	0.0039	0.87	5.3E-10	168
Smoking Initiation	rs12130857	1:7791461	G	0.0180	0.0027	0.70	3.7E-11	168
Smoking Initiation	rs12195240	6:98636905	G	-0.0249	0.0028	0.69	1.1E-18	328
Smoking Initiation	rs12244388	10:104640052	G	-0.0258	0.0027	0.62	4.3E-22	388
Smoking Initiation	rs12474587	2:162802993	G	-0.0242	0.0026	0.63	4.8E-21	336
Smoking Initiation	rs12517438	5:30842054	T	-0.0154	0.0026	0.50	1.9E-09	145
Smoking Initiation	rs12563365	1:236872829	G	-0.0166	0.0026	0.46	1.1E-10	168
Smoking Initiation	rs12633090	3:83241365	G	0.0230	0.0033	0.84	3.2E-12	171
Smoking Initiation	rs12714017	2:80999398	T	-0.0154	0.0026	0.54	3.7E-09	145
Smoking Initiation	rs12739243	1:210302043	T	0.0213	0.0031	0.73	4.4E-12	218
Smoking Initiation	rs12740789	1:72752073	G	0.0285	0.0033	0.86	1.2E-17	245
Smoking Initiation	rs12755632	1:41776623	A	0.0154	0.0027	0.67	1.9E-08	130
Smoking Initiation	rs12855717	13:101252635	C	-0.0155	0.0026	0.43	1.2E-09	146
Smoking Initiation	rs12878369	14:28346502	C	-0.0174	0.0026	0.65	1.6E-11	170
Smoking Initiation	rs12918191	16:50945156	A	0.0197	0.0030	0.80	3.1E-11	155
Smoking Initiation	rs1291821	10:11133823	A	-0.0145	0.0026	0.46	1.4E-08	128
Smoking Initiation	rs13007361	2:166250244	G	-0.0175	0.0031	0.85	2.3E-08	99
Smoking Initiation	rs13066050	3:81325861	C	-0.0188	0.0031	0.81	1.9E-09	137
Smoking Initiation	rs13109980	4:140886963	G	0.0222	0.0027	0.68	3.4E-16	266
Smoking Initiation	rs13110073	4:147797913	T	0.0246	0.0026	0.57	3.2E-21	366

Smoking Initiation	rs13237637	7:3503207	G	0.0237	0.0025	0.49	1.5E-20	345
Smoking Initiation	rs13261666	8:59814666	G	0.0200	0.0025	0.46	4.4E-15	245
Smoking Initiation	rs13392222	2:100672408	A	0.0234	0.0037	0.83	1.9E-10	195
Smoking Initiation	rs13437771	7:99071478	A	0.0271	0.0035	0.78	1.4E-14	308
Smoking Initiation	rs1373178	18:49967811	T	0.0203	0.0026	0.39	4.2E-15	241
Smoking Initiation	rs1381287	14:98597552	C	-0.0180	0.0026	0.52	1.8E-12	200
Smoking Initiation	rs1381775	11:42442826	T	0.0156	0.0028	0.28	2.8E-08	122
Smoking Initiation	rs1385108	5:154839646	C	-0.0187	0.0030	0.73	3.8E-10	170
Smoking Initiation	rs13906	12:49952394	C	0.0245	0.0041	0.87	2.0E-09	165
Smoking Initiation	rs1435479	4:94550450	G	-0.0164	0.0028	0.70	5.7E-09	140
Smoking Initiation	rs1435672	15:36399479	T	-0.0141	0.0026	0.40	3.8E-08	118
Smoking Initiation	rs1445649	2:155682556	T	-0.0206	0.0026	0.49	8.5E-16	261
Smoking Initiation	rs1449012	3:159048333	C	0.0154	0.0026	0.60	1.8E-09	139
Smoking Initiation	rs147052174	1:179783167	G	-0.0623	0.0098	0.99	2.3E-10	117
Smoking Initiation	rs1514176	1:74991596	G	0.0193	0.0026	0.47	7.7E-14	229
Smoking Initiation	rs1518393	2:58171220	A	-0.0169	0.0026	0.41	1.3E-10	169
Smoking Initiation	rs1549979	3:85460131	C	0.0245	0.0026	0.34	8.8E-21	335
Smoking Initiation	rs160631	6:52895230	T	0.0173	0.0029	0.28	1.9E-09	149
Smoking Initiation	rs1632941	6:29796685	T	0.0158	0.0026	0.64	6.7E-10	142
Smoking Initiation	rs16826827	2:147825689	T	0.0222	0.0039	0.88	9.2E-09	129
Smoking Initiation	rs16828799	3:173353739	G	-0.0198	0.0035	0.84	1.8E-08	127
Smoking Initiation	rs1713676	11:113660576	A	0.0167	0.0026	0.46	5.4E-11	171
Smoking Initiation	rs1714521	3:158284861	A	0.0163	0.0026	0.57	3.1E-10	160
Smoking Initiation	rs17165769	5:107365642	A	-0.0159	0.0026	0.59	9.6E-10	151
Smoking Initiation	rs17197663	13:38172867	G	0.0216	0.0039	0.90	2.1E-08	105
Smoking Initiation	rs17229285	2:199523122	C	0.0155	0.0025	0.54	1.3E-09	147
Smoking Initiation	rs1733760	10:56698174	T	-0.0148	0.0025	0.54	6.7E-09	134
Smoking Initiation	rs1737329	6:163807748	C	-0.0170	0.0029	0.25	5.1E-09	133

Smoking Initiation	rs17554906	6:92226609	G	-0.0142	0.0026	0.62	3.1E-08	117
Smoking Initiation	rs1759433	9:128073097	G	-0.0154	0.0026	0.50	1.7E-09	145
Smoking Initiation	rs17616642	2:59022210	A	0.0166	0.0030	0.79	2.1E-08	110
Smoking Initiation	rs17692129	17:44793283	C	-0.0196	0.0027	0.58	4.6E-13	230
Smoking Initiation	rs1772572	13:81191176	C	0.0169	0.0027	0.67	5.6E-10	155
Smoking Initiation	rs1799068	7:97707069	G	-0.0166	0.0026	0.60	2.6E-10	164
Smoking Initiation	rs1811739	14:77529375	G	-0.0183	0.0030	0.75	6.0E-10	155
Smoking Initiation	rs1863161	2:60139524	G	-0.0153	0.0026	0.38	2.3E-09	136
Smoking Initiation	rs1889571	1:32195819	T	-0.0222	0.0038	0.84	4.2E-09	166
Smoking Initiation	rs1901477	2:104126983	A	-0.0304	0.0026	0.51	2.1E-31	570
Smoking Initiation	rs1927901	9:120519111	T	0.0142	0.0026	0.44	3.1E-08	122
Smoking Initiation	rs1930371	9:81444104	C	0.0172	0.0030	0.78	7.1E-09	125
Smoking Initiation	rs1931431	9:11161799	G	-0.0182	0.0026	0.53	8.6E-13	204
Smoking Initiation	rs1937443	1:66469643	C	-0.0204	0.0026	0.42	1.8E-15	250
Smoking Initiation	rs2010921	11:132098205	G	-0.0174	0.0028	0.68	2.5E-10	163
Smoking Initiation	rs2028269	5:79308315	G	-0.0162	0.0026	0.63	5.2E-10	151
Smoking Initiation	rs2063976	8:91096366	C	0.0202	0.0027	0.40	7.4E-14	241
Smoking Initiation	rs2155646	11:112912811	T	-0.0378	0.0026	0.45	9.4E-48	870
Smoking Initiation	rs2173019	5:167614971	T	-0.0282	0.0033	0.84	3.0E-17	267
Smoking Initiation	rs221988	3:64234307	A	0.0149	0.0026	0.58	1.4E-08	133
Smoking Initiation	rs2276825	3:52886605	T	-0.0189	0.0030	0.74	1.9E-10	170
Smoking Initiation	rs2279829	3:147106319	C	0.0174	0.0031	0.73	2.1E-08	145
Smoking Initiation	rs2289791	15:67476952	G	0.0177	0.0030	0.77	2.0E-09	139
Smoking Initiation	rs2306866	3:53766212	A	0.0167	0.0026	0.35	1.9E-10	156
Smoking Initiation	rs2319545	3:147719648	C	-0.0232	0.0036	0.84	8.3E-11	177
Smoking Initiation	rs2344976	17:30685935	T	0.0151	0.0026	0.38	8.0E-09	132
Smoking Initiation	rs2378662	9:86707289	G	-0.0152	0.0026	0.44	2.7E-09	141
Smoking Initiation	rs238896	11:113994505	G	0.0169	0.0025	0.52	3.7E-11	175

Smoking Initiation	rs2526390	3:50192760	C	-0.0205	0.0027	0.73	3.6E-14	204
Smoking Initiation	rs2539706	2:59819545	G	-0.0162	0.0026	0.47	2.0E-10	162
Smoking Initiation	rs2587507	17:77790135	T	0.0147	0.0025	0.49	8.7E-09	132
Smoking Initiation	rs2637869	1:38757237	G	-0.0182	0.0028	0.71	6.5E-11	169
Smoking Initiation	rs2710634	2:32808804	T	0.0178	0.0026	0.48	3.4E-12	194
Smoking Initiation	rs2734390	3:60459291	A	-0.0148	0.0026	0.67	2.1E-08	119
Smoking Initiation	rs2796793	10:36634124	G	-0.0145	0.0026	0.56	1.6E-08	127
Smoking Initiation	rs281296	15:47685010	G	-0.0247	0.0027	0.66	1.6E-20	338
Smoking Initiation	rs28408682	10:104403310	A	-0.0167	0.0026	0.55	1.4E-10	170
Smoking Initiation	rs28441558	17:7803118	T	0.0356	0.0055	0.94	1.2E-10	171
Smoking Initiation	rs2901785	1:174104743	G	0.0173	0.0026	0.55	1.5E-11	183
Smoking Initiation	rs290601	8:115374642	C	-0.0163	0.0029	0.70	1.1E-08	138
Smoking Initiation	rs2925128	14:98362355	C	-0.0168	0.0027	0.56	3.7E-10	172
Smoking Initiation	rs2939756	11:41436297	G	0.0157	0.0026	0.50	7.5E-10	152
Smoking Initiation	rs2959084	11:46078656	G	-0.0171	0.0028	0.34	9.8E-10	160
Smoking Initiation	rs301807	1:8484823	A	-0.0180	0.0026	0.39	2.5E-12	190
Smoking Initiation	rs3115418	2:200936399	T	0.0142	0.0026	0.51	2.8E-08	125
Smoking Initiation	rs3172494	3:48731487	G	0.0291	0.0040	0.81	3.4E-13	320
Smoking Initiation	rs3218116	6:41901763	C	0.0198	0.0029	0.76	1.0E-11	175
Smoking Initiation	rs329124	5:133865452	A	0.0164	0.0026	0.58	2.0E-10	162
Smoking Initiation	rs34342129	18:5872472	T	0.0143	0.0025	0.53	2.1E-08	125
Smoking Initiation	rs34399632	2:137571174	A	-0.0194	0.0030	0.78	1.5E-10	158
Smoking Initiation	rs34553878	9:134334588	A	-0.0247	0.0041	0.91	1.2E-09	122
Smoking Initiation	rs34940743	14:80102233	A	-0.0159	0.0027	0.66	2.8E-09	140
Smoking Initiation	rs35375873	5:43190647	G	0.0270	0.0041	0.84	3.3E-11	236
Smoking Initiation	rs35656245	1:190957480	G	-0.0159	0.0029	0.74	2.2E-08	122
Smoking Initiation	rs357304	2:164862639	T	-0.0167	0.0029	0.28	5.4E-09	137
Smoking Initiation	rs359247	2:60477052	A	-0.0220	0.0027	0.35	9.9E-17	273

Smoking Initiation	rs359431	5:173288534	C	0.0142	0.0026	0.46	3.2E-08	123
Smoking Initiation	rs3740977	11:46393574	T	-0.0195	0.0034	0.86	1.2E-08	112
Smoking Initiation	rs3800227	6:108994161	A	-0.0172	0.0029	0.32	3.6E-09	157
Smoking Initiation	rs3810496	20:62406886	T	-0.0159	0.0026	0.39	1.5E-09	148
Smoking Initiation	rs3811038	2:113240183	T	-0.0191	0.0028	0.76	1.6E-11	165
Smoking Initiation	rs3820277	1:18436657	G	0.0188	0.0026	0.47	1.6E-13	218
Smoking Initiation	rs3843905	5:165427280	C	0.0151	0.0026	0.57	5.4E-09	138
Smoking Initiation	rs3847244	9:3025368	C	-0.0187	0.0026	0.59	2.6E-13	208
Smoking Initiation	rs3850736	8:64912021	C	-0.0191	0.0026	0.48	6.4E-14	225
Smoking Initiation	rs3909281	5:165096435	T	-0.0211	0.0026	0.53	1.6E-16	273
Smoking Initiation	rs3934797	4:112467612	G	0.0213	0.0033	0.84	1.1E-10	151
Smoking Initiation	rs4044321	5:166989513	A	0.0226	0.0027	0.35	1.8E-17	288
Smoking Initiation	rs4140932	4:15458598	T	0.0140	0.0026	0.53	4.9E-08	121
Smoking Initiation	rs42417	5:94198290	C	-0.0169	0.0028	0.43	8.3E-10	173
Smoking Initiation	rs4264267	13:38359676	C	-0.0148	0.0026	0.45	6.8E-09	133
Smoking Initiation	rs4310804	15:96858409	C	0.0182	0.0030	0.71	7.5E-10	167
Smoking Initiation	rs4326350	8:10763655	C	0.0176	0.0026	0.59	5.2E-12	186
Smoking Initiation	rs4476253	18:25253297	G	0.0185	0.0030	0.77	5.8E-10	149
Smoking Initiation	rs4543050	3:74954560	A	-0.0222	0.0033	0.17	1.5E-11	168
Smoking Initiation	rs45444697	1:155034632	C	-0.0197	0.0031	0.82	2.7E-10	141
Smoking Initiation	rs4674916	2:225365635	C	0.0180	0.0027	0.69	3.1E-11	171
Smoking Initiation	rs4674993	2:226332033	A	0.0240	0.0032	0.80	4.9E-14	229
Smoking Initiation	rs4727189	7:88442568	T	-0.0149	0.0027	0.66	3.0E-08	123
Smoking Initiation	rs4752018	10:118678712	C	-0.0189	0.0030	0.79	4.4E-10	146
Smoking Initiation	rs4759229	12:56474480	A	-0.0156	0.0027	0.33	6.5E-09	132
Smoking Initiation	rs4785187	16:49766772	G	-0.0200	0.0031	0.72	6.6E-11	198
Smoking Initiation	rs4788676	16:72950468	T	0.0177	0.0030	0.83	4.9E-09	107
Smoking Initiation	rs4790874	17:1995177	C	-0.0174	0.0026	0.49	8.4E-12	187

Smoking Initiation	rs4818005	21:40588819	G	0.0204	0.0026	0.36	1.1E-14	236
Smoking Initiation	rs4822102	22:42698430	C	0.0165	0.0026	0.46	2.8E-10	167
Smoking Initiation	rs4837631	9:122061948	C	0.0154	0.0026	0.56	2.0E-09	143
Smoking Initiation	rs4877285	9:81354129	G	0.0181	0.0027	0.35	2.1E-11	184
Smoking Initiation	rs4886207	13:60705792	T	0.0162	0.0026	0.42	8.8E-10	158
Smoking Initiation	rs4912332	1:58815243	C	-0.0141	0.0025	0.47	2.9E-08	123
Smoking Initiation	rs540860	11:121530888	A	-0.0176	0.0026	0.45	5.8E-12	189
Smoking Initiation	rs55786907	13:59871584	A	-0.0194	0.0035	0.82	1.8E-08	138
Smoking Initiation	rs55913542	14:99693843	G	-0.0186	0.0034	0.82	3.3E-08	124
Smoking Initiation	rs55944129	4:29082156	T	0.0176	0.0029	0.70	1.1E-09	159
Smoking Initiation	rs56208390	2:83247997	A	-0.0216	0.0039	0.86	2.7E-08	134
Smoking Initiation	rs56902655	15:63898709	T	0.0219	0.0037	0.85	4.1E-09	154
Smoking Initiation	rs58400863	4:31184484	G	0.0202	0.0027	0.66	4.9E-14	226
Smoking Initiation	rs586699	11:92289734	G	0.0148	0.0026	0.43	7.3E-09	132
Smoking Initiation	rs59537158	4:28246049	C	-0.0225	0.0031	0.82	4.6E-13	184
Smoking Initiation	rs6011779	20:61984317	C	0.0192	0.0032	0.24	2.8E-09	166
Smoking Initiation	rs6050446	20:25195509	A	-0.0544	0.0076	0.03	8.8E-13	222
Smoking Initiation	rs6073075	20:42015801	T	0.0187	0.0034	0.17	2.4E-08	121
Smoking Initiation	rs60833441	15:74048768	A	0.0143	0.0026	0.53	2.3E-08	125
Smoking Initiation	rs61533748	2:22582968	T	-0.0174	0.0026	0.58	2.8E-11	183
Smoking Initiation	rs61884449	11:64485193	C	-0.0200	0.0036	0.80	2.3E-08	154
Smoking Initiation	rs61886926	11:64133552	C	0.0179	0.0026	0.60	7.3E-12	190
Smoking Initiation	rs619087	6:94175279	A	-0.0143	0.0026	0.51	3.1E-08	125
Smoking Initiation	rs61959481	13:55834929	G	0.0203	0.0031	0.81	7.9E-11	154
Smoking Initiation	rs62007780	15:78025464	G	0.0159	0.0026	0.61	7.5E-10	149
Smoking Initiation	rs62052916	16:72574550	A	0.0319	0.0050	0.92	1.6E-10	191
Smoking Initiation	rs62098013	18:50863861	G	-0.0177	0.0026	0.66	2.2E-11	174
Smoking Initiation	rs62106258	2:417167	T	0.0455	0.0060	0.97	3.3E-14	127

Smoking Initiation	rs62137126	2:44250149	A	0.0237	0.0039	0.89	1.3E-09	139
Smoking Initiation	rs62180324	2:63416606	G	0.0195	0.0031	0.83	3.9E-10	131
Smoking Initiation	rs62193862	2:202843875	G	-0.0238	0.0042	0.88	2.0E-08	146
Smoking Initiation	rs62246017	3:71483084	G	0.0162	0.0027	0.72	3.0E-09	131
Smoking Initiation	rs62340589	4:176875795	G	-0.0174	0.0032	0.81	4.3E-08	117
Smoking Initiation	rs62618693	11:32956492	C	0.0353	0.0063	0.97	2.1E-08	85
Smoking Initiation	rs6265	11:27679916	C	0.0293	0.0033	0.83	2.8E-19	304
Smoking Initiation	rs6437769	3:107997514	C	-0.0142	0.0026	0.48	3.7E-08	124
Smoking Initiation	rs6438436	3:117822149	C	-0.0247	0.0033	0.24	5.3E-14	271
Smoking Initiation	rs644740	11:65561468	C	0.0141	0.0026	0.58	3.7E-08	119
Smoking Initiation	rs6452785	5:87685500	C	0.0269	0.0026	0.51	4.7E-26	445
Smoking Initiation	rs6497840	16:25351633	G	-0.0228	0.0029	0.30	2.0E-15	270
Smoking Initiation	rs6568832	6:97702876	G	-0.0189	0.0030	0.28	1.7E-10	179
Smoking Initiation	rs67050670	18:39297254	A	0.0203	0.0030	0.78	2.3E-11	171
Smoking Initiation	rs6731872	2:624205	T	-0.0316	0.0034	0.18	5.4E-21	363
Smoking Initiation	rs6750107	2:80748807	G	-0.0146	0.0026	0.58	2.6E-08	128
Smoking Initiation	rs6750529	2:182027603	C	-0.0199	0.0029	0.26	9.3E-12	188
Smoking Initiation	rs6756212	2:146140132	C	0.0339	0.0026	0.52	3.5E-40	706
Smoking Initiation	rs67777803	17:27323322	G	0.0246	0.0034	0.82	3.2E-13	220
Smoking Initiation	rs6782116	3:77176032	C	0.0147	0.0026	0.66	1.5E-08	118
Smoking Initiation	rs6874731	5:80263865	T	-0.0153	0.0025	0.54	1.8E-09	144
Smoking Initiation	rs6890961	5:166778503	C	0.0193	0.0026	0.37	2.1E-13	214
Smoking Initiation	rs6936160	6:100347745	C	-0.0201	0.0028	0.31	4.2E-13	214
Smoking Initiation	rs6948707	7:1870794	T	-0.0243	0.0026	0.60	4.2E-21	351
Smoking Initiation	rs6968380	7:114940159	G	0.0234	0.0027	0.36	1.1E-17	311
Smoking Initiation	rs6986430	8:93048104	T	0.0243	0.0031	0.80	2.0E-15	235
Smoking Initiation	rs7024924	9:8282399	T	-0.0189	0.0034	0.81	1.9E-08	137
Smoking Initiation	rs7026534	9:134907263	T	0.0166	0.0028	0.29	2.7E-09	140

Smoking Initiation	rs7072776	10:22032942	A	0.0220	0.0028	0.26	5.7E-15	231
Smoking Initiation	rs7134009	12:75263193	T	0.0158	0.0029	0.67	4.3E-08	137
Smoking Initiation	rs71367544	18:77574374	C	-0.0206	0.0032	0.79	8.5E-11	171
Smoking Initiation	rs71592686	5:60121271	T	-0.0207	0.0029	0.73	3.8E-13	209
Smoking Initiation	rs71602617	4:136406155	C	0.0178	0.0032	0.73	2.1E-08	153
Smoking Initiation	rs7188873	16:24727064	A	-0.0203	0.0026	0.37	8.5E-15	238
Smoking Initiation	rs7192140	16:10173748	T	0.0169	0.0025	0.54	3.4E-11	175
Smoking Initiation	rs72780746	5:103929588	T	0.0258	0.0034	0.84	2.1E-14	217
Smoking Initiation	rs72789626	5:106825618	T	0.0256	0.0037	0.90	5.1E-12	147
Smoking Initiation	rs72790288	2:29513404	G	0.0455	0.0077	0.98	3.3E-09	108
Smoking Initiation	rs72898831	18:42658643	A	0.0244	0.0035	0.86	4.1E-12	178
Smoking Initiation	rs72938304	18:53661743	G	0.0272	0.0040	0.89	1.4E-11	175
Smoking Initiation	rs73008357	6:156431856	A	0.0223	0.0040	0.89	2.4E-08	120
Smoking Initiation	rs7333559	13:100546450	G	0.0232	0.0031	0.20	5.9E-14	216
Smoking Initiation	rs73831818	3:55988394	A	-0.0320	0.0055	0.95	5.5E-09	127
Smoking Initiation	rs748832	3:16851202	A	-0.0172	0.0026	0.64	6.6E-11	168
Smoking Initiation	rs7505855	18:31696075	C	0.0170	0.0026	0.43	5.3E-11	174
Smoking Initiation	rs75674569	13:96823724	G	0.0253	0.0043	0.93	2.6E-09	106
Smoking Initiation	rs75919030	17:50193197	T	0.0210	0.0029	0.73	3.3E-13	214
Smoking Initiation	rs7598402	2:50735943	C	0.0147	0.0025	0.49	7.4E-09	134
Smoking Initiation	rs7600835	2:172521827	G	0.0151	0.0027	0.71	1.8E-08	116
Smoking Initiation	rs7631379	3:181409057	T	-0.0208	0.0032	0.83	3.9E-11	151
Smoking Initiation	rs7640107	3:59966156	C	0.0142	0.0026	0.55	3.5E-08	123
Smoking Initiation	rs76460663	11:111979741	C	0.0423	0.0064	0.93	4.1E-11	289
Smoking Initiation	rs7657022	4:35501032	A	-0.0183	0.0025	0.50	7.3E-13	206
Smoking Initiation	rs76608582	19:4474725	C	0.0345	0.0059	0.94	4.9E-09	153
Smoking Initiation	rs76841737	7:91281409	C	0.0231	0.0042	0.89	3.3E-08	126
Smoking Initiation	rs7696257	4:137474783	G	-0.0153	0.0026	0.58	6.8E-09	141

Smoking Initiation	rs77215829	12:112618346	A	0.0240	0.0038	0.86	2.0E-10	172
Smoking Initiation	rs77283305	7:132593831	G	0.0152	0.0028	0.71	3.9E-08	117
Smoking Initiation	rs7743165	6:67521222	T	-0.0193	0.0025	0.54	4.2E-14	227
Smoking Initiation	rs7802996	7:77771983	C	0.0209	0.0034	0.88	1.1E-09	112
Smoking Initiation	rs7809303	7:69484366	G	0.0214	0.0027	0.66	3.5E-15	252
Smoking Initiation	rs7836565	8:52569449	C	0.0155	0.0028	0.30	4.4E-08	125
Smoking Initiation	rs7867822	9:20676454	A	0.0151	0.0027	0.31	2.8E-08	121
Smoking Initiation	rs7920501	10:10043159	T	0.0155	0.0026	0.59	1.3E-09	144
Smoking Initiation	rs7921378	10:63674885	G	0.0233	0.0025	0.52	6.1E-20	334
Smoking Initiation	rs7929518	11:85980958	A	-0.0192	0.0030	0.26	2.6E-10	176
Smoking Initiation	rs7943721	11:73309393	G	0.0212	0.0034	0.16	3.6E-10	151
Smoking Initiation	rs7969559	12:69655167	A	0.0170	0.0028	0.34	1.5E-09	159
Smoking Initiation	rs8005334	14:79563654	T	-0.0167	0.0027	0.72	3.4E-10	138
Smoking Initiation	rs8027457	15:99204101	T	-0.0153	0.0025	0.48	1.9E-09	144
Smoking Initiation	rs8050598	16:49891964	C	-0.0187	0.0029	0.77	1.8E-10	154
Smoking Initiation	rs8096225	18:36921851	A	-0.0155	0.0028	0.27	2.6E-08	116
Smoking Initiation	rs8103660	19:18566395	T	-0.0158	0.0027	0.62	3.0E-09	146
Smoking Initiation	rs876793	1:237852083	T	0.0179	0.0027	0.71	5.7E-11	164
Smoking Initiation	rs910912	20:54462393	T	0.0168	0.0029	0.26	7.8E-09	134
Smoking Initiation	rs925524	1:46496709	A	-0.0156	0.0028	0.28	2.9E-08	121
Smoking Initiation	rs9288999	3:114147927	G	-0.0174	0.0029	0.30	1.5E-09	158
Smoking Initiation	rs9302604	16:69576894	A	-0.0187	0.0026	0.56	3.3E-13	212
Smoking Initiation	rs9323328	14:58653514	A	0.0142	0.0026	0.52	2.6E-08	125
Smoking Initiation	rs9331343	6:157738258	T	0.0141	0.0026	0.43	3.9E-08	120
Smoking Initiation	rs9423279	10:125680419	C	0.0186	0.0027	0.40	3.1E-12	204
Smoking Initiation	rs951740	1:44011737	G	-0.0295	0.0026	0.38	3.8E-29	508
Smoking Initiation	rs9538162	13:59265043	T	-0.0174	0.0026	0.59	1.8E-11	180
Smoking Initiation	rs9540731	13:66949370	C	0.0177	0.0025	0.53	3.4E-12	193

Smoking Initiation	rs9545155	13:80191873	T	0.0161	0.0026	0.53	3.0E-10	159
Smoking Initiation	rs9627272	22:46442288	G	0.0155	0.0026	0.64	2.4E-09	136
Smoking Initiation	rs9826984	3:131945722	G	0.0141	0.0026	0.49	3.9E-08	122
Smoking Initiation	rs9841807	3:175718927	C	-0.0163	0.0029	0.68	1.4E-08	141
Smoking Initiation	rs9850597	3:161761866	G	0.0186	0.0033	0.23	1.6E-08	150
Smoking Initiation	rs986714	5:50821338	A	0.0160	0.0026	0.58	4.1E-10	155
Smoking Initiation	rs9922607	16:17570220	C	0.0222	0.0032	0.87	3.4E-12	139
Smoking Initiation	rs9941217	16:18050926	C	0.0186	0.0027	0.67	3.5E-12	188
Smoking Initiation	rs9987376	8:93190014	T	0.0205	0.0026	0.38	2.0E-15	242
Smoking Index	rs10052591	5:50812738	T	0.0120	0.0020	0.59	2.1E-09	32
Smoking Index	rs10226228	7:32315613	A	-0.0160	0.0020	0.68	2.0E-15	52
Smoking Index	rs10282292	7:111092478	C	0.0130	0.0020	0.38	5.9E-10	37
Smoking Index	rs1050847	16:87443734	C	0.0110	0.0020	0.50	1.4E-08	28
Smoking Index	rs10823968	10:74738269	A	0.0120	0.0020	0.52	2.1E-08	33
Smoking Index	rs10879871	12:75380511	T	-0.0140	0.0020	0.37	5.0E-11	42
Smoking Index	rs10918701	1:162090536	G	0.0120	0.0020	0.40	2.1E-08	32
Smoking Index	rs10922907	1:91193049	A	0.0150	0.0020	0.55	3.0E-13	51
Smoking Index	rs11210229	1:73860028	A	0.0170	0.0020	0.39	2.0E-16	64
Smoking Index	rs112282219	11:46632809	G	-0.0330	0.0050	0.97	3.8E-11	31
Smoking Index	rs11255908	10:8802912	T	-0.0150	0.0020	0.77	2.3E-10	37
Smoking Index	rs113382419	9:136463019	C	-0.0410	0.0030	0.94	3.0E-37	87
Smoking Index	rs11768481	7:96629103	C	0.0130	0.0020	0.60	9.9E-10	38
Smoking Index	rs11783093	8:27425349	C	0.0230	0.0030	0.87	1.2E-16	56
Smoking Index	rs11861214	16:746611	G	0.0140	0.0020	0.68	2.0E-08	39
Smoking Index	rs1193237	1:7526486	G	-0.0110	0.0020	0.51	2.8E-08	28
Smoking Index	rs11948770	5:13246336	T	-0.0150	0.0020	0.75	4.9E-10	39
Smoking Index	rs12202536	6:67475273	A	-0.0120	0.0020	0.55	2.8E-09	33
Smoking Index	rs1221148	9:122046875	C	0.0130	0.0020	0.60	7.3E-11	38

Smoking Index	rs12244388	10:104640052	G	-0.0190	0.0020	0.62	1.4E-19	79
Smoking Index	rs1246265	9:86761745	T	-0.0130	0.0020	0.30	4.2E-09	33
Smoking Index	rs12481282	20:44761377	G	0.0160	0.0030	0.73	7.8E-09	47
Smoking Index	rs12623702	2:202885506	A	-0.0140	0.0020	0.57	7.7E-12	45
Smoking Index	rs12708665	16:24728227	A	-0.0220	0.0040	0.28	3.5E-09	91
Smoking Index	rs12831617	12:84758368	C	-0.0130	0.0020	0.82	1.9E-08	24
Smoking Index	rs12967855	18:35138245	A	0.0120	0.0020	0.31	3.1E-08	29
Smoking Index	rs13009008	2:174043233	A	0.0120	0.0020	0.38	4.6E-09	31
Smoking Index	rs13016665	2:57995348	C	-0.0130	0.0020	0.54	1.8E-09	39
Smoking Index	rs13153393	5:167604213	A	-0.0200	0.0030	0.90	2.5E-10	33
Smoking Index	rs13296519	9:128471924	G	-0.0140	0.0020	0.63	8.1E-12	42
Smoking Index	rs136233	22:31212410	A	-0.0140	0.0030	0.82	1.8E-08	26
Smoking Index	rs147412694	21:40702786	G	-0.0170	0.0030	0.87	2.9E-09	31
Smoking Index	rs17309874	11:27667236	G	-0.0160	0.0020	0.74	9.7E-13	45
Smoking Index	rs17553262	10:92912773	A	-0.0180	0.0030	0.92	5.3E-09	22
Smoking Index	rs17576594	4:147952241	G	0.0160	0.0020	0.72	1.7E-12	48
Smoking Index	rs1922018	7:3560401	C	0.0140	0.0020	0.33	3.0E-12	40
Smoking Index	rs1931263	1:96175101	G	-0.0110	0.0020	0.57	4.0E-08	27
Smoking Index	rs1933270	1:49977965	T	0.0130	0.0020	0.41	1.5E-10	38
Smoking Index	rs202645	22:41798520	A	-0.0150	0.0020	0.24	3.9E-09	38
Smoking Index	rs2062882	8:91839576	G	-0.0120	0.0020	0.56	1.1E-08	33
Smoking Index	rs2080870	5:60388313	A	0.0120	0.0020	0.24	4.9E-08	25
Smoking Index	rs2254710	6:37477000	C	0.0130	0.0020	0.31	3.5E-08	34
Smoking Index	rs2401924	7:115057862	G	0.0150	0.0020	0.55	2.7E-14	52
Smoking Index	rs245774	5:170530930	A	-0.0130	0.0020	0.31	7.4E-09	33
Smoking Index	rs2675638	10:63576286	G	0.0120	0.0020	0.60	1.3E-09	32
Smoking Index	rs2678670	2:104469564	A	0.0130	0.0020	0.47	3.1E-10	39
Smoking Index	rs2838834	21:46665208	C	-0.0130	0.0020	0.72	6.3E-10	32

Smoking Index	rs28485305	15:74044197	C	-0.0120	0.0020	0.61	2.6E-08	32
Smoking Index	rs2867112	2:651349	T	0.0210	0.0030	0.84	4.8E-15	56
Smoking Index	rs2890772	2:146175106	G	-0.0200	0.0020	0.33	2.1E-22	82
Smoking Index	rs2894808	6:52861990	T	-0.0130	0.0020	0.92	3.5E-09	12
Smoking Index	rs317021	4:35418368	T	-0.0170	0.0030	0.82	1.1E-10	40
Smoking Index	rs326341	3:107811142	G	0.0140	0.0020	0.56	1.2E-11	45
Smoking Index	rs329120	5:133861756	C	0.0140	0.0020	0.58	6.3E-12	44
Smoking Index	rs34866095	11:16377356	A	-0.0120	0.0020	0.73	1.2E-08	26
Smoking Index	rs348809	20:59032097	A	-0.0120	0.0020	0.33	1.3E-08	29
Smoking Index	rs35169606	8:9604066	T	-0.0130	0.0020	0.65	1.2E-09	36
Smoking Index	rs35175834	15:47680815	G	-0.0240	0.0020	0.79	4.6E-22	89
Smoking Index	rs35343344	19:18471610	C	0.0130	0.0020	0.74	8.8E-09	30
Smoking Index	rs359243	2:60475509	T	-0.0130	0.0020	0.38	9.5E-10	37
Smoking Index	rs369230	16:89645437	G	-0.0120	0.0020	0.34	1.8E-09	30
Smoking Index	rs3742365	14:104198251	T	-0.0160	0.0020	0.58	2.5E-14	58
Smoking Index	rs3769949	2:166199284	T	-0.0130	0.0020	0.65	2.5E-09	36
Smoking Index	rs3811038	2:113240183	T	-0.0140	0.0020	0.76	8.9E-10	33
Smoking Index	rs3896224	10:106467853	A	-0.0140	0.0020	0.53	1.1E-11	45
Smoking Index	rs421983	3:84892866	T	0.0130	0.0020	0.47	3.3E-10	39
Smoking Index	rs4391802	11:28674592	A	0.0150	0.0020	0.73	1.4E-11	41
Smoking Index	rs4473348	2:182073742	A	-0.0150	0.0020	0.24	6.4E-11	38
Smoking Index	rs4543592	9:3014254	T	-0.0120	0.0020	0.59	4.5E-10	32
Smoking Index	rs4571506	5:87756918	C	-0.0160	0.0030	0.53	1.5E-08	59
Smoking Index	rs4671357	2:60136176	T	0.0140	0.0020	0.44	1.1E-11	45
Smoking Index	rs4731925	7:132664757	C	0.0120	0.0020	0.30	2.6E-08	28
Smoking Index	rs4814873	20:19616429	C	0.0140	0.0020	0.76	2.9E-09	33
Smoking Index	rs4949465	1:32178489	T	-0.0170	0.0030	0.83	1.7E-08	38
Smoking Index	rs4957528	5:106420589	A	-0.0150	0.0020	0.17	4.2E-09	29

Smoking Index	rs549845	1:44076469	G	0.0160	0.0020	0.30	8.3E-14	50
Smoking Index	rs57611503	16:31165795	G	0.0110	0.0020	0.51	4.0E-08	28
Smoking Index	rs6011779	20:61984317	C	0.0280	0.0030	0.24	2.3E-27	133
Smoking Index	rs60952428	16:75640521	T	0.0190	0.0030	0.88	3.0E-08	35
Smoking Index	rs6119897	20:31145415	G	-0.0180	0.0020	0.69	3.6E-15	64
Smoking Index	rs61796681	4:23678196	A	-0.0190	0.0040	0.91	4.2E-08	27
Smoking Index	rs62098013	18:50863861	G	-0.0120	0.0020	0.66	4.1E-09	30
Smoking Index	rs62135536	2:44326028	C	0.0350	0.0060	0.96	8.0E-10	42
Smoking Index	rs62155874	2:105973094	A	-0.0240	0.0030	0.89	5.2E-16	52
Smoking Index	rs62175972	2:161362830	T	0.0310	0.0060	0.98	1.7E-08	19
Smoking Index	rs624833	4:2881256	T	0.0130	0.0020	0.67	6.6E-10	34
Smoking Index	rs6562474	13:67332812	C	0.0120	0.0020	0.58	1.0E-08	32
Smoking Index	rs6598539	15:99204483	T	-0.0120	0.0020	0.48	4.5E-09	33
Smoking Index	rs6741228	2:22548774	T	0.0110	0.0020	0.50	1.6E-08	28
Smoking Index	rs67596067	17:50333733	G	0.0130	0.0020	0.70	1.2E-09	33
Smoking Index	rs6778080	3:49317338	T	-0.0140	0.0020	0.23	1.3E-12	32
Smoking Index	rs6779302	3:16859710	G	-0.0130	0.0020	0.64	1.2E-09	36
Smoking Index	rs6935954	6:26255451	A	0.0140	0.0020	0.36	8.2E-12	42
Smoking Index	rs6957896	7:132309592	C	-0.0110	0.0020	0.57	4.5E-08	27
Smoking Index	rs6962772	7:99081730	A	-0.0130	0.0020	0.78	7.8E-09	27
Smoking Index	rs7039819	9:82430418	G	0.0130	0.0020	0.45	5.1E-10	39
Smoking Index	rs7077678	10:104438565	C	0.0120	0.0020	0.60	2.6E-09	32
Smoking Index	rs71367545	18:77576337	G	-0.0150	0.0020	0.80	1.4E-09	34
Smoking Index	rs7155595	14:77502546	A	-0.0120	0.0020	0.70	2.5E-09	28
Smoking Index	rs71627581	5:43161351	G	0.0190	0.0030	0.84	1.6E-09	44
Smoking Index	rs72674867	8:95578201	A	0.0130	0.0020	0.66	3.8E-08	35
Smoking Index	rs72678864	4:112422145	G	0.0180	0.0030	0.86	1.6E-11	36
Smoking Index	rs7297175	12:56473808	T	-0.0120	0.0020	0.42	6.6E-09	33

Smoking Index	rs732083	17:37834367	G	0.0110	0.0020	0.33	1.5E-08	25
Smoking Index	rs73220544	3:131074511	A	0.0120	0.0020	0.78	1.5E-08	23
Smoking Index	rs7333559	13:100546450	G	0.0150	0.0020	0.20	3.2E-10	34
Smoking Index	rs74086911	12:50015942	G	0.0210	0.0040	0.94	2.1E-08	23
Smoking Index	rs7519626	1:99514554	C	0.0120	0.0020	0.37	1.2E-08	31
Smoking Index	rs7528604	1:66407352	G	0.0140	0.0020	0.60	5.7E-12	44
Smoking Index	rs7553348	1:75005067	G	0.0140	0.0020	0.50	5.2E-12	45
Smoking Index	rs7569203	2:45154418	A	-0.0160	0.0020	0.61	7.4E-13	56
Smoking Index	rs75742406	11:17070365	G	0.0140	0.0020	0.73	1.3E-09	36
Smoking Index	rs76608582	19:4474725	C	0.0310	0.0050	0.94	3.2E-10	46
Smoking Index	rs775758	3:77582005	A	0.0120	0.0020	0.47	1.1E-08	33
Smoking Index	rs7766610	6:111707821	C	0.0180	0.0030	0.27	2.2E-12	59
Smoking Index	rs7807019	7:117543063	A	-0.0150	0.0020	0.46	6.7E-14	52
Smoking Index	rs8042134	15:97514404	T	0.0160	0.0020	0.53	1.3E-12	59
Smoking Index	rs8042849	15:78817929	C	0.0280	0.0020	0.37	1.8E-39	170
Smoking Index	rs860326	14:57342912	C	0.0120	0.0020	0.53	2.7E-09	33
Smoking Index	rs8614	17:27588806	C	-0.0170	0.0030	0.82	1.8E-10	39
Smoking Index	rs889398	16:69556715	C	0.0130	0.0020	0.59	6.3E-11	38
Smoking Index	rs9435340	1:107593201	T	0.0120	0.0020	0.32	1.2E-08	29
Smoking Index	rs9842947	3:157412246	C	-0.0130	0.0020	0.32	3.1E-09	34
Smoking Index	rs986391	5:166993972	G	0.0160	0.0020	0.37	9.4E-15	55
Smoking Index	rs9904288	17:47031973	T	0.0120	0.0020	0.73	3.1E-08	26
Smoking Index	rs9919670	11:112877304	G	-0.0220	0.0020	0.45	7.6E-27	111
Alcohol Drinking	rs10004020	4:152968372	A	0.0091	0.0016	0.62	2.4E-08	36
Alcohol Drinking	rs10028756	4:100254520	A	-0.0186	0.0022	0.11	1.2E-17	61
Alcohol Drinking	rs1004787	2:45159091	A	0.0084	0.0015	0.61	8.4E-09	32
Alcohol Drinking	rs10085696	7:69783020	G	-0.0114	0.0019	0.21	1.1E-09	40
Alcohol Drinking	rs10236149	7:98977515	G	-0.0135	0.0022	0.18	1.2E-09	50

Alcohol Drinking	rs10438820	17:78524597	T	0.0090	0.0016	0.65	1.8E-08	35
Alcohol Drinking	rs10506274	12:81601464	T	-0.0090	0.0015	0.45	5.8E-10	38
Alcohol Drinking	rs10750025	11:113424042	T	0.0103	0.0016	0.77	4.9E-11	36
Alcohol Drinking	rs10753661	1:165119792	A	-0.0086	0.0016	0.70	3.8E-08	30
Alcohol Drinking	rs10876188	12:51895882	T	-0.0080	0.0015	0.48	4.8E-08	30
Alcohol Drinking	rs10978550	9:109345993	C	-0.0117	0.0018	0.22	7.2E-11	44
Alcohol Drinking	rs11030084	11:27643725	T	-0.0106	0.0019	0.17	1.7E-08	30
Alcohol Drinking	rs1104608	16:73912588	C	-0.0110	0.0015	0.42	1.1E-13	55
Alcohol Drinking	rs1123285	14:57274519	G	-0.0089	0.0015	0.34	8.1E-09	34
Alcohol Drinking	rs113443718	16:29892184	A	-0.0102	0.0016	0.30	1.2E-10	41
Alcohol Drinking	rs1154414	4:100000136	C	0.0176	0.0021	0.15	3.7E-17	73
Alcohol Drinking	rs11625650	14:104610138	A	-0.0096	0.0017	0.19	2.9E-08	27
Alcohol Drinking	rs11692435	2:98275354	A	0.0174	0.0026	0.21	2.5E-11	94
Alcohol Drinking	rs11739827	5:166803321	T	-0.0084	0.0015	0.50	1.2E-08	33
Alcohol Drinking	rs11940694	4:39414993	G	0.0259	0.0015	0.61	3.0E-68	302
Alcohol Drinking	rs12088813	1:66407700	C	-0.0093	0.0016	0.26	1.6E-08	31
Alcohol Drinking	rs1217091	8:64527399	C	0.0122	0.0019	0.81	7.1E-11	42
Alcohol Drinking	rs1229984	4:100239319	C	0.1505	0.0039	0.89	1.6E-203	4131
Alcohol Drinking	rs12499107	4:99678691	G	0.0127	0.0022	0.13	4.5E-09	34
Alcohol Drinking	rs1260326	2:27730940	C	0.0209	0.0015	0.63	8.1E-45	191
Alcohol Drinking	rs12651313	4:171086393	G	-0.0086	0.0015	0.49	3.8E-09	35
Alcohol Drinking	rs12655091	5:144412335	A	-0.0083	0.0015	0.49	1.3E-08	32
Alcohol Drinking	rs12795042	11:133658168	C	-0.0083	0.0015	0.67	3.3E-08	29
Alcohol Drinking	rs12907323	15:86796012	G	0.0085	0.0015	0.36	9.9E-09	31
Alcohol Drinking	rs13024996	2:144225215	A	-0.0109	0.0015	0.32	5.7E-13	49
Alcohol Drinking	rs13032049	2:63581507	G	0.0102	0.0016	0.26	3.0E-10	38
Alcohol Drinking	rs13066454	3:93994255	T	-0.0088	0.0015	0.41	4.1E-09	35
Alcohol Drinking	rs13094887	3:70968431	T	-0.0103	0.0016	0.27	8.6E-11	39

Alcohol Drinking	rs13107325	4:103188709	T	-0.0275	0.0028	0.05	1.5E-22	63
Alcohol Drinking	rs13250583	8:20949917	T	-0.0097	0.0018	0.24	4.7E-08	32
Alcohol Drinking	rs13383034	2:45155276	T	0.0149	0.0016	0.38	6.3E-22	99
Alcohol Drinking	rs144198753	4:99713350	T	-0.0418	0.0059	0.02	1.4E-12	57
Alcohol Drinking	rs17029090	4:100443853	G	-0.0491	0.0052	0.03	4.8E-21	122
Alcohol Drinking	rs1713676	11:113660576	G	-0.0080	0.0015	0.54	4.3E-08	30
Alcohol Drinking	rs17177078	16:24810681	T	-0.0223	0.0030	0.07	1.3E-13	57
Alcohol Drinking	rs17665139	10:125093880	T	-0.0116	0.0020	0.13	1.6E-08	29
Alcohol Drinking	rs2011092	3:141124607	C	-0.0089	0.0015	0.35	7.4E-09	34
Alcohol Drinking	rs2165670	4:100286085	A	0.0231	0.0024	0.13	1.7E-22	112
Alcohol Drinking	rs2178197	2:27860551	G	-0.0088	0.0015	0.54	2.5E-09	36
Alcohol Drinking	rs2180870	14:58782779	C	-0.0122	0.0021	0.12	1.1E-08	30
Alcohol Drinking	rs2472297	15:75027880	T	0.0106	0.0017	0.24	3.1E-10	38
Alcohol Drinking	rs2532276	17:44246624	A	-0.0218	0.0026	0.08	1.6E-17	68
Alcohol Drinking	rs2764771	16:20013793	A	0.0099	0.0016	0.37	4.0E-10	43
Alcohol Drinking	rs281379	19:49214274	A	0.0137	0.0015	0.41	4.9E-21	86
Alcohol Drinking	rs2854334	17:29715500	G	0.0092	0.0015	0.59	7.5E-10	39
Alcohol Drinking	rs28601761	8:126500031	G	0.0091	0.0015	0.40	7.2E-10	37
Alcohol Drinking	rs28680958	1:173848808	A	-0.0110	0.0018	0.21	5.1E-10	38
Alcohol Drinking	rs28929474	14:94844947	T	-0.0368	0.0054	0.02	1.3E-11	48
Alcohol Drinking	rs35034355	7:103840115	A	-0.0081	0.0015	0.48	2.9E-08	31
Alcohol Drinking	rs35538052	4:39418965	A	-0.0085	0.0015	0.36	1.4E-08	32
Alcohol Drinking	rs36052336	4:100273594	G	-0.0184	0.0030	0.06	1.2E-09	38
Alcohol Drinking	rs3748034	4:3446091	T	-0.0117	0.0021	0.13	1.7E-08	30
Alcohol Drinking	rs378421	16:28754684	A	-0.0112	0.0015	0.34	4.8E-14	53
Alcohol Drinking	rs3803800	17:7462969	G	0.0114	0.0018	0.75	1.5E-10	46
Alcohol Drinking	rs3809162	12:54674235	G	0.0091	0.0015	0.46	1.2E-09	38
Alcohol Drinking	rs4092465	18:55080437	G	-0.0083	0.0015	0.61	4.4E-08	31

Alcohol Drinking	rs4501255	4:42151306	G	0.0107	0.0017	0.23	4.8E-10	38
Alcohol Drinking	rs4548913	17:2209888	A	-0.0084	0.0015	0.61	3.1E-08	31
Alcohol Drinking	rs4690727	4:143648579	G	0.0108	0.0016	0.73	2.4E-11	44
Alcohol Drinking	rs4699791	4:101243023	A	0.0186	0.0025	0.13	6.6E-14	75
Alcohol Drinking	rs4815364	20:25035711	A	0.0086	0.0015	0.63	1.0E-08	32
Alcohol Drinking	rs4842786	12:92170791	A	-0.0088	0.0015	0.61	2.7E-09	35
Alcohol Drinking	rs4916723	5:87854395	C	-0.0100	0.0015	0.44	1.7E-11	46
Alcohol Drinking	rs4938230	11:116075001	A	0.0128	0.0020	0.81	1.5E-10	47
Alcohol Drinking	rs500321	13:27124360	T	-0.0097	0.0017	0.75	4.9E-09	33
Alcohol Drinking	rs5024204	1:71491890	T	0.0097	0.0016	0.28	2.6E-09	35
Alcohol Drinking	rs55872084	5:155902003	T	0.0100	0.0017	0.18	6.3E-09	28
Alcohol Drinking	rs55932213	9:108755622	G	0.0095	0.0017	0.72	9.6E-09	34
Alcohol Drinking	rs56030824	11:47397353	A	-0.0116	0.0016	0.31	1.2E-13	55
Alcohol Drinking	rs56337305	2:225475560	C	-0.0096	0.0015	0.35	1.6E-10	39
Alcohol Drinking	rs58107686	1:33837334	A	-0.0097	0.0016	0.33	7.8E-10	39
Alcohol Drinking	rs60654199	3:141267295	A	-0.0167	0.0030	0.06	2.9E-08	30
Alcohol Drinking	rs62044525	16:64872590	G	-0.0122	0.0019	0.15	1.0E-10	36
Alcohol Drinking	rs62250685	3:85457240	G	-0.0144	0.0015	0.66	1.1E-21	88
Alcohol Drinking	rs6460047	7:73042443	C	0.0116	0.0018	0.18	9.7E-11	37
Alcohol Drinking	rs6787172	3:158187811	G	-0.0080	0.0015	0.53	4.3E-08	30
Alcohol Drinking	rs682011	11:121544285	C	0.0082	0.0015	0.59	2.2E-08	31
Alcohol Drinking	rs6951574	7:153489744	C	0.0132	0.0015	0.39	1.6E-19	78
Alcohol Drinking	rs705687	1:4548453	G	-0.0109	0.0018	0.80	8.2E-10	35
Alcohol Drinking	rs7074871	10:110507806	A	-0.0094	0.0017	0.24	1.9E-08	31
Alcohol Drinking	rs7185555	16:69131281	C	-0.0111	0.0020	0.13	4.2E-08	26
Alcohol Drinking	rs72859280	2:147956293	T	0.0229	0.0039	0.04	4.4E-09	41
Alcohol Drinking	rs77165542	2:430975	T	-0.0260	0.0040	0.02	5.6E-11	24
Alcohol Drinking	rs79139602	4:100444363	T	0.0603	0.0051	0.04	1.8E-32	276

Alcohol Drinking	rs7950166	11:8642218	T	-0.0098	0.0015	0.63	9.9E-11	42
Alcohol Drinking	rs79616692	16:72338507	C	0.0163	0.0024	0.12	4.1E-12	52
Alcohol Drinking	rs823114	1:205719532	A	0.0088	0.0015	0.56	2.3E-09	36
Alcohol Drinking	rs828867	2:74334462	A	0.0088	0.0015	0.59	2.2E-09	35
Alcohol Drinking	rs9607814	22:41946519	A	-0.0102	0.0019	0.22	4.3E-08	34
Alcohol Drinking	rs9838144	3:131576287	C	-0.0100	0.0018	0.21	2.7E-08	31
Alcohol Drinking	rs9950000	18:53052169	T	-0.0091	0.0015	0.39	9.4E-10	37
Alcohol Dependence	rs1229984	4:100239319	T	-0.7215	0.1025	0.11	9.8E-13	5197
Alcohol Dependence	rs3811802	4:100244221	G	0.1501	0.0275	0.49	2.4E-08	531
Alcohol Dependence	rs894368	4:100309313	A	-0.1199	0.0218	0.28	1.9E-08	269
Coffee consumption	rs1057868	7:75615006	T	0.0394	0.0032	0.36	5.3E-33	269
Coffee consumption	rs10865548	2:631606	G	0.0308	0.0038	0.82	4.5E-15	104
Coffee consumption	rs1260326	2:27730940	C	0.0272	0.0030	0.63	2.6E-19	129
Coffee consumption	rs1956218	14:33075243	G	0.0164	0.0030	0.49	3.6E-08	51
Coffee consumption	rs2330783	22:24747031	G	0.0906	0.0126	0.99	1.6E-12	71
Coffee consumption	rs2472297	15:75027880	T	0.0908	0.0034	0.24	5.2E-155	1121
Coffee consumption	rs34060476	7:73037956	G	0.0378	0.0044	0.13	5.1E-18	121
Coffee consumption	rs4410790	7:17284577	C	0.0788	0.0030	0.63	5.6E-141	1093
Coffee consumption	rs574367	1:177873210	T	0.0210	0.0036	0.19	8.1E-09	50
Coffee consumption	rs597045	11:56272114	A	0.0214	0.0032	0.67	6.6E-11	76
Coffee consumption	rs66723169	18:57808978	A	0.0294	0.0036	0.21	9.9E-17	107
Coffee consumption	rs73073176	7:17562952	C	0.0462	0.0044	0.90	5.6E-25	145
Caffeine consumption	rs2470893	15:75019449	T	0.1200	0.0160	0.27	5.2E-14	57
Caffeine consumption	rs4410790	7:17284577	C	0.1500	0.0170	0.63	2.4E-19	105
Sleep Duration	rs10173260	2:210377845	C	0.0128	0.0023	0.57	2.9E-08	36
Sleep Duration	rs10421649	19:9942262	A	0.0133	0.0023	0.50	6.9E-09	39
Sleep Duration	rs10483350	14:29816155	G	0.0174	0.0029	0.20	1.5E-09	44
Sleep Duration	rs1057703	11:122830251	G	0.0194	0.0032	0.15	1.1E-09	43

Sleep Duration	rs10761674	10:64618340	C	0.0123	0.0023	0.47	4.2E-08	34
Sleep Duration	rs10973207	9:37100525	T	0.0204	0.0031	0.21	6.0E-11	63
Sleep Duration	rs11190970	10:103128332	G	0.0154	0.0028	0.82	4.6E-08	31
Sleep Duration	rs112230981	3:55879269	A	0.0315	0.0052	0.97	2.2E-09	27
Sleep Duration	rs113113059	6:43160375	T	0.0161	0.0027	0.80	8.4E-09	38
Sleep Duration	rs11567976	5:137654218	T	0.0128	0.0023	0.51	2.1E-08	37
Sleep Duration	rs11602180	11:48162453	C	0.0183	0.0031	0.84	2.3E-09	41
Sleep Duration	rs11621908	14:78495761	C	0.0241	0.0042	0.93	5.6E-09	33
Sleep Duration	rs11643715	16:23909538	G	0.0139	0.0025	0.27	3.2E-08	34
Sleep Duration	rs11885663	2:166944004	T	0.0162	0.0026	0.21	8.6E-10	39
Sleep Duration	rs12246842	10:21830580	A	0.0134	0.0023	0.45	3.9E-09	40
Sleep Duration	rs12567114	1:98527951	A	0.0148	0.0025	0.27	4.3E-09	38
Sleep Duration	rs12607679	18:53059748	T	0.0201	0.0026	0.76	8.3E-15	66
Sleep Duration	rs12611523	2:139195328	A	0.0126	0.0023	0.60	3.1E-08	34
Sleep Duration	rs1263056	11:116576415	A	0.0128	0.0023	0.44	2.0E-08	36
Sleep Duration	rs12791153	11:80685181	T	0.0236	0.0042	0.07	1.9E-08	34
Sleep Duration	rs13088093	3:135838598	G	0.0163	0.0024	0.30	7.0E-12	49
Sleep Duration	rs13109404	4:102896591	T	0.0312	0.0044	0.96	1.4E-12	32
Sleep Duration	rs151014368	5:176751059	A	0.0161	0.0028	0.23	9.1E-09	41
Sleep Duration	rs1517572	11:28829882	C	0.0147	0.0023	0.64	1.5E-10	44
Sleep Duration	rs1553132	11:88297740	G	0.0145	0.0026	0.24	2.5E-08	35
Sleep Duration	rs17427571	4:82254908	A	0.0138	0.0024	0.65	1.3E-08	39
Sleep Duration	rs174560	11:61581764	C	0.0136	0.0024	0.35	2.8E-08	38
Sleep Duration	rs17732997	3:70470834	C	0.0129	0.0023	0.61	1.2E-08	35
Sleep Duration	rs1776776	9:140497072	T	0.0200	0.0034	0.82	4.9E-09	52
Sleep Duration	rs1939455	11:101520886	G	0.0204	0.0036	0.91	1.2E-08	32
Sleep Duration	rs1991556	17:44083402	G	0.0166	0.0027	0.91	1.0E-09	20
Sleep Duration	rs205024	17:11227352	T	0.0138	0.0023	0.39	3.9E-09	41

Sleep Duration	rs2072727	20:43538733	T	0.0133	0.0023	0.46	7.9E-09	39
Sleep Duration	rs2079070	7:114126432	C	0.0176	0.0026	0.22	7.5E-12	47
Sleep Duration	rs2192528	4:18327896	A	0.0134	0.0023	0.54	2.7E-09	40
Sleep Duration	rs2231265	6:89790201	G	0.0150	0.0027	0.80	2.7E-08	32
Sleep Duration	rs269054	1:57864304	A	0.0137	0.0023	0.36	2.1E-09	38
Sleep Duration	rs3095508	16:6550400	C	0.0154	0.0023	0.57	3.1E-11	52
Sleep Duration	rs330088	8:9149746	C	0.0145	0.0023	0.59	2.7E-10	45
Sleep Duration	rs34354917	12:38764559	C	0.0138	0.0025	0.75	3.9E-08	32
Sleep Duration	rs34731055	7:2106928	T	0.0195	0.0030	0.22	3.7E-11	58
Sleep Duration	rs35531607	4:92533225	C	0.0128	0.0023	0.43	1.5E-08	36
Sleep Duration	rs365663	5:1428883	A	0.0146	0.0023	0.46	1.0E-10	47
Sleep Duration	rs374153	2:40382712	C	0.0176	0.0031	0.20	9.1E-09	44
Sleep Duration	rs4128364	2:147612734	C	0.0146	0.0024	0.35	1.4E-09	43
Sleep Duration	rs4538155	2:157040773	T	0.0130	0.0024	0.64	3.6E-08	35
Sleep Duration	rs4592416	11:43800474	G	0.0147	0.0023	0.48	9.3E-11	48
Sleep Duration	rs460692	5:3126584	C	0.0211	0.0033	0.20	3.6E-10	64
Sleep Duration	rs4767550	12:117951150	G	0.0143	0.0023	0.42	6.3E-10	44
Sleep Duration	rs55658675	14:65554638	C	0.0131	0.0024	0.64	2.0E-08	35
Sleep Duration	rs56372231	5:102321905	T	0.0170	0.0024	0.33	2.2E-12	57
Sleep Duration	rs61796569	1:66476437	T	0.0155	0.0026	0.27	1.5E-09	42
Sleep Duration	rs61985058	14:60233841	T	0.0186	0.0032	0.10	1.3E-08	29
Sleep Duration	rs62120041	2:9185564	T	0.0261	0.0046	0.92	9.6E-09	42
Sleep Duration	rs6575005	14:26954078	T	0.0156	0.0027	0.78	4.4E-09	37
Sleep Duration	rs7115226	11:113408518	A	0.0266	0.0044	0.06	1.7E-09	38
Sleep Duration	rs72804080	2:59358659	G	0.0178	0.0032	0.16	2.9E-08	38
Sleep Duration	rs73219758	8:14279446	G	0.0164	0.0025	0.75	5.6E-11	45
Sleep Duration	rs7503199	17:8134275	C	0.0148	0.0026	0.76	1.0E-08	35
Sleep Duration	rs75539574	2:58871658	C	0.0363	0.0041	0.08	6.9E-19	89

Sleep Duration	rs7556815	2:114085785	A	0.0407	0.0027	0.22	1.3E-49	255
Sleep Duration	rs7644809	3:107564459	T	0.0131	0.0023	0.45	1.6E-08	38
Sleep Duration	rs7806045	7:132610266	T	0.0148	0.0026	0.73	1.4E-08	38
Sleep Duration	rs7915425	10:125016501	T	0.0191	0.0030	0.17	2.0E-10	47
Sleep Duration	rs7951019	11:118358027	G	0.0369	0.0065	0.03	1.2E-08	37
Sleep Duration	rs80193650	6:33464363	G	0.0168	0.0031	0.13	4.1E-08	28
Sleep Duration	rs8038326	15:47989799	A	0.0159	0.0025	0.71	2.8E-10	46
Sleep Duration	rs8050478	16:56120461	G	0.0160	0.0023	0.54	1.7E-12	57
Sleep Duration	rs915416	1:34731984	C	0.0193	0.0025	0.31	9.9E-15	71
Sleep Duration	rs9345234	6:93162639	C	0.0130	0.0023	0.54	1.8E-08	38
Sleep Duration	rs9382445	6:54937974	T	0.0145	0.0023	0.62	4.8E-10	44
Sleep Duration	rs9903973	17:50571227	C	0.0128	0.0023	0.44	2.6E-08	36
Sleep Duration	rs9940646	16:53800629	C	0.0170	0.0023	0.58	1.2E-13	63
Short Sleep Duration	rs11763750	7:2080114	G	0.0350	0.0080	0.74	5.1E-09	210
Short Sleep Duration	rs1229762	7:114218582	T	0.0370	0.0030	0.68	1.1E-12	265
Short Sleep Duration	rs12518468	5:7249696	C	0.0310	0.0060	0.33	8.5E-09	191
Short Sleep Duration	rs12567114	1:98527951	G	0.0360	0.0070	0.73	4.1E-09	226
Short Sleep Duration	rs12661667	6:41792545	T	0.0280	0.0070	0.24	2.8E-08	128
Short Sleep Duration	rs12963463	18:53099093	C	0.0290	0.0060	0.28	1.9E-11	152
Short Sleep Duration	rs13107325	4:103188709	T	0.0750	0.0110	0.05	2.5E-13	220
Short Sleep Duration	rs1380703	2:57941287	G	0.0350	0.0060	0.37	1.6E-11	256
Short Sleep Duration	rs1607227	11:28808617	G	0.0310	0.0070	0.73	1.5E-09	169
Short Sleep Duration	rs17005118	4:82288564	A	0.0300	0.0070	0.25	2.5E-09	151
Short Sleep Duration	rs17388803	15:48027204	C	0.0530	0.0100	0.09	6.5E-10	207
Short Sleep Duration	rs2014830	3:50172397	C	0.0300	0.0060	0.66	2.7E-08	180
Short Sleep Duration	rs205024	17:11227352	C	0.0310	0.0060	0.61	2.7E-08	205
Short Sleep Duration	rs2186122	1:66470206	T	0.0240	0.0060	0.58	4.8E-09	125
Short Sleep Duration	rs2820313	1:201870221	G	0.0310	0.0060	0.30	2.3E-09	181

Short Sleep Duration	rs2863957	2:114089551	C	0.0540	0.0070	0.78	2.6E-18	449
Short Sleep Duration	rs3776864	5:102327868	A	0.0310	0.0060	0.68	1.7E-08	186
Short Sleep Duration	rs4585442	5:135508381	G	0.0310	0.0060	0.30	8.1E-10	180
Short Sleep Duration	rs5757675	22:39838892	G	0.0340	0.0070	0.27	2.7E-09	204
Short Sleep Duration	rs59779556	16:56227965	T	0.0250	0.0060	0.51	2.0E-08	139
Short Sleep Duration	rs60882754	8:52886619	A	0.0550	0.0120	0.96	1.8E-08	108
Short Sleep Duration	rs7524118	1:34736052	C	0.0300	0.0060	0.69	4.9E-08	172
Short Sleep Duration	rs75539574	2:58871658	A	0.0450	0.0110	0.92	8.4E-11	137
Short Sleep Duration	rs7939345	11:47980568	T	0.0350	0.0070	0.20	4.0E-08	174
Short Sleep Duration	rs9321171	6:129848635	C	0.0310	0.0060	0.61	4.2E-08	205
Short Sleep Duration	rs9367621	6:55040290	T	0.0240	0.0060	0.43	1.6E-08	126
Long Sleep Duration	rs10899257	11:76415209	A	0.0680	0.0130	0.14	4.6E-08	511
Long Sleep Duration	rs17688916	17:43778680	T	0.0710	0.0120	0.94	1.1E-11	260
Long Sleep Duration	rs17817288	16:53807764	A	0.0390	0.0090	0.51	8.9E-09	339
Long Sleep Duration	rs3751046	11:122828342	G	0.0700	0.0130	0.16	2.0E-08	599
Long Sleep Duration	rs6737318	2:114083120	G	0.0760	0.0110	0.22	3.4E-13	884
Long Sleep Duration	rs7534398	1:7767464	A	0.0470	0.0120	0.20	2.1E-08	311
Long Sleep Duration	rs75458655	11:118115331	T	0.1850	0.0290	0.02	5.4E-12	643
Insomnia	rs1015438	16:51177517	A	0.0583	0.0080	0.22	2.5E-14	1572
Insomnia	rs1031654	13:54382035	A	-0.0513	0.0070	0.79	3.9E-12	1164
Insomnia	rs1038093	15:74012409	T	0.0392	0.0060	0.60	2.5E-10	986
Insomnia	rs10502966	18:50748499	A	-0.0387	0.0060	0.59	8.5E-11	965
Insomnia	rs1064939	11:118396331	A	0.1302	0.0200	0.98	2.2E-10	932
Insomnia	rs10756571	9:14534505	T	0.0363	0.0060	0.66	1.8E-08	786
Insomnia	rs10758593	9:4292083	A	-0.0356	0.0060	0.43	4.9E-09	830
Insomnia	rs10761240	9:96361922	A	-0.0429	0.0060	0.39	2.1E-12	1164
Insomnia	rs10800992	1:190900576	T	0.0421	0.0060	0.41	3.8E-12	1144
Insomnia	rs10825503	10:57177470	T	0.0334	0.0060	0.42	1.4E-08	723

Insomnia	rs10865954	3:49211989	T	0.0421	0.0060	0.30	1.9E-11	984
Insomnia	rs10898940	11:73455292	A	0.0344	0.0060	0.57	8.1E-09	775
Insomnia	rs10928256	2:146458738	T	0.0344	0.0060	0.47	1.6E-08	785
Insomnia	rs10944696	6:94498850	A	-0.0377	0.0070	0.32	8.0E-09	826
Insomnia	rs10947428	6:33647058	T	-0.0683	0.0070	0.84	9.1E-21	1636
Insomnia	rs10947690	6:37631768	A	-0.0471	0.0070	0.78	4.0E-12	1015
Insomnia	rs10947987	6:41754370	T	-0.0325	0.0060	0.45	4.1E-08	698
Insomnia	rs10955647	8:114154187	T	0.0334	0.0060	0.56	1.8E-08	733
Insomnia	rs11001276	10:76825638	A	-0.0377	0.0070	0.71	2.5E-08	775
Insomnia	rs11090039	22:41496800	A	0.0392	0.0070	0.30	1.8E-09	858
Insomnia	rs11119409	1:210293333	T	-0.0346	0.0060	0.52	1.2E-08	795
Insomnia	rs11149313	13:85294881	A	0.0402	0.0070	0.62	2.4E-09	1011
Insomnia	rs113851554	2:66750564	T	0.2062	0.0140	0.07	1.6E-51	7024
Insomnia	rs1147852	6:147980909	A	0.0392	0.0060	0.29	9.9E-10	852
Insomnia	rs11588755	1:57819204	A	-0.0346	0.0060	0.44	5.1E-09	783
Insomnia	rs11605348	11:47606483	A	-0.0450	0.0060	0.35	7.0E-13	1222
Insomnia	rs116466468	2:159137557	T	0.0440	0.0070	0.74	2.1E-10	1005
Insomnia	rs11650304	17:46035001	C	0.0667	0.0120	0.93	1.2E-08	801
Insomnia	rs1167132	12:43484487	T	0.0354	0.0060	0.38	8.7E-09	783
Insomnia	rs11679943	2:77724624	A	0.0373	0.0060	0.37	3.2E-09	867
Insomnia	rs117152417	6:166411281	A	-0.1473	0.0260	0.01	2.8E-08	589
Insomnia	rs11722569	4:112822731	T	0.0344	0.0060	0.67	2.9E-08	702
Insomnia	rs11756035	6:18843810	C	0.0507	0.0090	0.10	1.3E-08	610
Insomnia	rs11803128	1:190060095	A	-0.0408	0.0060	0.65	6.8E-11	1014
Insomnia	rs118166957	9:8858043	T	0.0677	0.0080	0.13	1.9E-16	1421
Insomnia	rs12030482	1:96961268	A	0.0411	0.0070	0.20	8.2E-09	711
Insomnia	rs12187443	5:102660400	T	0.0402	0.0060	0.67	1.6E-10	953
Insomnia	rs12251016	10:21821918	A	-0.0387	0.0060	0.68	3.9E-10	876

Insomnia	rs12310246	12:84700945	A	0.0450	0.0070	0.21	4.7E-11	881
Insomnia	rs12454003	18:26315799	C	-0.0346	0.0060	0.46	4.9E-09	790
Insomnia	rs12520974	5:61514611	T	-0.0356	0.0060	0.49	1.7E-09	845
Insomnia	rs12605642	18:31313965	T	0.0354	0.0060	0.54	2.1E-09	829
Insomnia	rs12666306	7:115082406	A	0.0421	0.0060	0.55	2.2E-12	1169
Insomnia	rs12790660	11:57667222	T	-0.0398	0.0060	0.66	4.5E-10	942
Insomnia	rs1289939	1:117944435	T	-0.0408	0.0070	0.26	6.0E-09	864
Insomnia	rs12912299	15:38897857	T	-0.0429	0.0060	0.46	4.4E-13	1220
Insomnia	rs12917449	15:74331659	A	-0.0419	0.0080	0.76	3.0E-08	845
Insomnia	rs12924275	16:9191790	T	0.0383	0.0070	0.28	1.9E-08	789
Insomnia	rs12983032	19:5073447	A	-0.0429	0.0060	0.42	1.1E-11	1195
Insomnia	rs12991815	2:68071990	C	0.0402	0.0060	0.44	3.0E-11	1058
Insomnia	rs13010288	2:51824512	T	-0.0598	0.0090	0.10	9.3E-12	872
Insomnia	rs13135092	4:103198082	A	-0.0888	0.0110	0.95	2.5E-16	957
Insomnia	rs13138995	4:148987430	A	0.0344	0.0060	0.38	2.0E-08	743
Insomnia	rs138678612	6:30932223	A	-0.1165	0.0200	0.98	1.4E-08	685
Insomnia	rs1519102	2:66677816	C	-0.0367	0.0060	0.67	1.9E-08	786
Insomnia	rs152555	5:106849674	A	-0.0523	0.0080	0.85	4.8E-10	920
Insomnia	rs1530938	2:236900633	A	0.0363	0.0060	0.46	8.8E-10	874
Insomnia	rs1536053	13:111982291	T	-0.0377	0.0060	0.29	6.0E-09	781
Insomnia	rs1567084	3:71435955	A	0.0334	0.0060	0.53	2.1E-08	742
Insomnia	rs1580173	3:107955515	A	0.0334	0.0060	0.48	2.3E-08	743
Insomnia	rs1620977	1:72729142	A	0.0516	0.0070	0.27	2.3E-14	1407
Insomnia	rs16903122	5:87693561	T	0.0554	0.0070	0.24	9.0E-16	1502
Insomnia	rs16990210	4:34720226	T	-0.0460	0.0080	0.84	2.0E-08	749
Insomnia	rs17005118	4:82288564	A	0.0421	0.0070	0.25	6.1E-10	886
Insomnia	rs17025198	3:88001713	A	0.0411	0.0070	0.25	2.2E-08	835
Insomnia	rs17083297	5:92995477	A	-0.0440	0.0080	0.11	1.6E-08	485

Insomnia	rs17223714	5:50492629	A	0.0459	0.0070	0.75	2.4E-10	1041
Insomnia	rs1731951	7:137075847	A	-0.0346	0.0060	0.45	1.4E-08	788
Insomnia	rs17367725	5:107112116	T	-0.0356	0.0060	0.30	9.3E-09	716
Insomnia	rs17520265	7:119674508	A	-0.0910	0.0160	0.02	2.9E-08	498
Insomnia	rs17643634	8:91650818	T	-0.0598	0.0080	0.15	1.3E-13	1189
Insomnia	rs176644	15:89913632	T	0.0354	0.0060	0.43	9.5E-09	816
Insomnia	rs1861412	2:58893065	A	0.0383	0.0060	0.48	1.7E-10	974
Insomnia	rs190073	7:10985188	A	-0.0336	0.0060	0.45	2.9E-08	742
Insomnia	rs1927902	9:120518991	T	0.0526	0.0070	0.27	1.2E-14	1452
Insomnia	rs1937447	1:66358242	C	-0.0387	0.0070	0.70	2.1E-08	836
Insomnia	rs2030672	7:21687925	C	0.0344	0.0060	0.54	1.1E-08	783
Insomnia	rs2089358	1:37194103	T	-0.0408	0.0070	0.67	2.7E-10	986
Insomnia	rs214934	11:17193475	A	-0.0377	0.0060	0.33	3.2E-09	836
Insomnia	rs2216427	3:180785697	C	0.0354	0.0060	0.68	1.6E-08	724
Insomnia	rs2221119	11:88598444	C	0.0363	0.0060	0.47	2.0E-09	876
Insomnia	rs224029	10:64519299	T	-0.0387	0.0060	0.40	2.5E-10	962
Insomnia	rs2286729	12:6873818	A	0.0695	0.0110	0.06	5.4E-11	747
Insomnia	rs2364921	3:158522463	T	-0.0336	0.0060	0.51	2.1E-08	749
Insomnia	rs2388840	6:99598756	A	-0.0367	0.0060	0.64	1.4E-09	826
Insomnia	rs2389631	13:96932868	A	-0.0398	0.0060	0.59	2.0E-10	1023
Insomnia	rs2431108	5:103947968	T	-0.0534	0.0060	0.70	7.8E-17	1605
Insomnia	rs2598293	7:133989882	T	0.0354	0.0060	0.43	2.5E-09	816
Insomnia	rs2737240	8:116657235	A	0.0363	0.0070	0.69	3.4E-08	752
Insomnia	rs2792990	9:125621610	C	0.0545	0.0080	0.82	1.2E-10	1193
Insomnia	rs2838787	21:46539725	A	-0.0356	0.0060	0.38	7.7E-09	796
Insomnia	rs28552587	8:103356226	A	0.0334	0.0060	0.63	3.3E-08	693
Insomnia	rs28582096	12:123856998	A	-0.0545	0.0070	0.21	1.7E-13	1323
Insomnia	rs28611339	8:10170037	T	0.0583	0.0090	0.11	8.5E-11	858

Insomnia	rs2867690	20:41972028	T	0.0421	0.0080	0.18	3.7E-08	692
Insomnia	rs3131638	6:31475127	A	-0.0440	0.0070	0.28	7.9E-10	1048
Insomnia	rs314281	6:105400605	T	-0.0429	0.0060	0.44	6.0E-13	1206
Insomnia	rs3184470	16:715164	A	-0.0377	0.0060	0.47	9.7E-10	942
Insomnia	rs324017	12:57487814	A	0.0392	0.0070	0.33	1.6E-09	901
Insomnia	rs34214423	16:52303107	A	0.0450	0.0080	0.82	3.2E-09	793
Insomnia	rs34490907	17:26933741	C	0.0535	0.0090	0.89	1.8E-08	734
Insomnia	rs34967082	2:215382654	A	0.0354	0.0060	0.39	4.3E-09	795
Insomnia	rs35110063	3:43066558	A	0.0392	0.0060	0.44	8.8E-11	1009
Insomnia	rs35322724	16:77137324	A	0.0488	0.0060	0.52	3.7E-16	1584
Insomnia	rs35539975	5:91607148	A	0.0421	0.0070	0.73	4.5E-09	929
Insomnia	rs3774751	3:50209053	T	-0.0408	0.0060	0.49	7.3E-12	1110
Insomnia	rs3902952	16:61647589	T	0.0478	0.0080	0.22	2.6E-10	1040
Insomnia	rs4090240	9:77118987	T	-0.0387	0.0070	0.26	8.5E-09	761
Insomnia	rs4238755	16:52746089	A	-0.0429	0.0070	0.28	2.3E-10	998
Insomnia	rs4260410	3:178469932	T	0.0344	0.0060	0.30	4.9E-08	667
Insomnia	rs429358	19:45411941	T	0.0459	0.0080	0.83	2.1E-08	781
Insomnia	rs4502882	5:153093998	T	-0.0387	0.0060	0.69	8.0E-10	856
Insomnia	rs4588900	8:73890425	A	0.0334	0.0060	0.52	1.6E-08	743
Insomnia	rs4592425	11:62697813	T	0.0402	0.0060	0.72	4.3E-10	861
Insomnia	rs4643373	17:47123423	T	0.0411	0.0070	0.72	1.6E-10	903
Insomnia	rs4664299	2:160570033	T	-0.0408	0.0070	0.22	4.9E-09	769
Insomnia	rs4699157	4:106055212	T	-0.0812	0.0150	0.97	4.0E-08	448
Insomnia	rs4702	15:91426560	A	-0.0481	0.0060	0.54	6.8E-16	1534
Insomnia	rs4709655	6:163280204	T	-0.0545	0.0090	0.09	3.1E-09	668
Insomnia	rs4767645	12:118385788	T	-0.0367	0.0060	0.53	6.5E-10	893
Insomnia	rs4788203	16:29978827	A	-0.0346	0.0060	0.41	6.3E-09	773
Insomnia	rs4858708	3:25154112	A	-0.0336	0.0060	0.55	1.2E-08	742

Insomnia	rs492858	3:155432229	T	-0.0661	0.0110	0.10	3.5E-09	1006
Insomnia	rs4981170	14:33412996	A	-0.0545	0.0080	0.19	7.3E-13	1211
Insomnia	rs521484	7:49894349	A	-0.0398	0.0070	0.77	1.5E-08	741
Insomnia	rs524859	11:66041079	A	-0.0440	0.0060	0.32	1.5E-12	1125
Insomnia	rs55772859	2:208042581	A	0.0421	0.0060	0.33	4.8E-11	1042
Insomnia	rs55972276	5:135653737	A	0.0733	0.0090	0.10	4.2E-17	1313
Insomnia	rs56097173	2:44262449	T	0.0402	0.0060	0.66	2.7E-10	966
Insomnia	rs56133505	11:72348039	A	0.0411	0.0060	0.45	5.6E-12	1114
Insomnia	rs566673	11:66401373	T	-0.0387	0.0060	0.60	1.2E-10	960
Insomnia	rs5877	1:173878862	T	0.0363	0.0060	0.68	1.2E-08	769
Insomnia	rs6019663	20:47774512	T	0.0402	0.0070	0.30	6.5E-10	898
Insomnia	rs60565673	18:52906830	T	-0.0429	0.0060	0.63	1.6E-12	1150
Insomnia	rs6119267	20:31163914	C	-0.0598	0.0060	0.60	2.3E-20	2294
Insomnia	rs61921611	12:66367726	T	-0.0440	0.0060	0.69	7.8E-12	1106
Insomnia	rs62068188	17:2400876	T	0.0488	0.0080	0.82	1.2E-09	921
Insomnia	rs62158170	2:114082175	A	0.0658	0.0070	0.79	1.2E-19	1916
Insomnia	rs62213452	2:210380152	T	0.0373	0.0070	0.25	2.4E-08	694
Insomnia	rs62264767	3:117642005	A	0.0649	0.0080	0.81	1.6E-14	1720
Insomnia	rs62301574	4:22050165	C	-0.0419	0.0070	0.79	1.4E-08	777
Insomnia	rs623025	1:201765094	T	-0.0377	0.0070	0.25	3.2E-08	712
Insomnia	rs62383308	5:165460085	A	-0.0598	0.0110	0.07	4.0E-08	647
Insomnia	rs62429521	6:140324582	A	0.0507	0.0080	0.19	1.8E-09	1040
Insomnia	rs6457796	6:34828553	T	-0.0387	0.0070	0.73	1.1E-08	788
Insomnia	rs6465151	7:88310899	T	0.0564	0.0090	0.14	1.9E-09	1023
Insomnia	rs647905	11:121534938	T	0.0334	0.0060	0.53	2.9E-08	742
Insomnia	rs6510033	19:30710785	A	-0.0367	0.0070	0.76	4.7E-08	657
Insomnia	rs6545798	2:60521311	A	-0.0408	0.0060	0.38	1.2E-11	1044
Insomnia	rs6562066	13:60532796	T	0.0392	0.0060	0.43	1.4E-10	1006

Insomnia	rs6589988	11:99126016	A	-0.0377	0.0060	0.72	4.7E-09	770
Insomnia	rs6597649	9:133786652	T	0.0334	0.0060	0.44	3.1E-08	733
Insomnia	rs6601080	5:179511043	A	0.0354	0.0060	0.64	2.2E-08	772
Insomnia	rs6606731	12:109982578	A	0.0431	0.0080	0.16	1.5E-08	649
Insomnia	rs66674044	16:19904344	A	-0.0598	0.0090	0.87	2.2E-12	1045
Insomnia	rs667730	11:83277325	T	0.0334	0.0060	0.60	2.3E-08	712
Insomnia	rs6702604	1:107190062	A	-0.0367	0.0060	0.56	1.3E-09	883
Insomnia	rs670501	7:108625185	T	0.0526	0.0070	0.24	7.4E-13	1344
Insomnia	rs671985	8:60914783	A	-0.0377	0.0060	0.48	2.8E-10	945
Insomnia	rs6734957	2:42813247	T	-0.0419	0.0070	0.22	1.8E-09	811
Insomnia	rs6756610	2:147480394	C	0.0373	0.0060	0.61	1.1E-09	878
Insomnia	rs6808140	3:10581380	T	0.0392	0.0060	0.52	5.4E-11	1023
Insomnia	rs6888135	5:141254063	A	0.0383	0.0060	0.49	1.2E-10	974
Insomnia	rs694786	3:173112907	T	-0.0440	0.0060	0.42	2.0E-13	1250
Insomnia	rs6967168	7:132672192	T	-0.0440	0.0070	0.74	1.4E-10	998
Insomnia	rs6978112	7:1966841	T	0.0344	0.0060	0.40	2.1E-08	756
Insomnia	rs699844	1:74878253	A	0.0602	0.0110	0.93	4.1E-08	624
Insomnia	rs701394	5:80296487	A	-0.0356	0.0060	0.64	6.8E-09	778
Insomnia	rs7040224	9:134886837	A	0.0373	0.0060	0.31	4.2E-09	786
Insomnia	rs7044885	9:81739348	C	-0.0408	0.0060	0.49	5.7E-12	1109
Insomnia	rs715338	15:57215867	A	0.0411	0.0060	0.59	7.9E-12	1089
Insomnia	rs7168238	15:66709386	C	0.0639	0.0110	0.08	1.8E-08	842
Insomnia	rs7214267	17:43157709	A	-0.0440	0.0060	0.54	5.1E-13	1278
Insomnia	rs72657797	4:90820809	T	-0.0555	0.0080	0.13	1.5E-12	901
Insomnia	rs72773790	9:139109080	T	0.0373	0.0060	0.69	3.7E-09	797
Insomnia	rs728017	6:124292594	A	-0.0346	0.0060	0.43	9.5E-09	780
Insomnia	rs72820274	2:104412924	A	0.0344	0.0060	0.42	1.3E-08	768
Insomnia	rs72899452	11:45415577	T	0.0742	0.0120	0.09	1.0E-09	1188

Insomnia	rs73671843	7:3520024	A	-0.0555	0.0090	0.14	5.5E-10	969
Insomnia	rs7402939	15:99183876	T	-0.0356	0.0060	0.39	5.2E-09	801
Insomnia	rs742760	20:50985290	A	0.0431	0.0080	0.83	2.5E-08	682
Insomnia	rs7475916	10:77771194	C	-0.0367	0.0060	0.37	6.7E-09	838
Insomnia	rs75452188	2:67134426	A	0.0516	0.0090	0.89	1.6E-08	691
Insomnia	rs7571486	2:176473295	A	-0.0387	0.0070	0.27	1.4E-08	784
Insomnia	rs75932578	7:106844694	T	-0.0398	0.0070	0.19	4.2E-08	640
Insomnia	rs7599697	2:239231477	T	-0.0367	0.0060	0.34	5.0E-09	809
Insomnia	rs76145129	20:62670427	T	-0.0502	0.0090	0.11	2.7E-08	647
Insomnia	rs7615602	3:18718055	C	-0.0398	0.0070	0.32	2.6E-09	924
Insomnia	rs7625896	3:44062561	A	0.0363	0.0060	0.65	5.3E-09	801
Insomnia	rs77641763	9:140265782	T	0.0714	0.0090	0.21	6.5E-15	2262
Insomnia	rs7992992	13:54721699	A	0.0507	0.0090	0.14	1.2E-08	825
Insomnia	rs8076183	17:61024696	T	-0.0377	0.0060	0.48	2.7E-10	944
Insomnia	rs8180457	5:107209814	T	-0.0555	0.0080	0.15	1.1E-11	1038
Insomnia	rs8180817	7:114047542	C	-0.0492	0.0060	0.46	1.8E-16	1602
Insomnia	rs823247	2:2850540	T	-0.0367	0.0060	0.46	5.3E-10	888
Insomnia	rs830716	16:12323509	C	0.0450	0.0070	0.61	8.7E-12	1278
Insomnia	rs871994	8:35190619	A	0.0354	0.0060	0.39	5.5E-09	794
Insomnia	rs874168	8:30849450	T	0.0344	0.0060	0.56	8.0E-09	777
Insomnia	rs908668	19:56134038	T	0.0497	0.0070	0.17	1.4E-11	943
Insomnia	rs910187	20:45841052	A	-0.0346	0.0060	0.35	1.6E-08	721
Insomnia	rs9373590	6:101212001	A	0.0402	0.0060	0.54	2.2E-11	1068
Insomnia	rs9394502	6:38452503	T	-0.0545	0.0060	0.39	7.8E-18	1886
Insomnia	rs9527083	13:53991125	A	-0.0758	0.0060	0.66	1.6E-32	3465
Insomnia	rs9540729	13:66947124	A	0.0363	0.0060	0.53	1.4E-09	875
Insomnia	rs9563886	13:61720066	T	-0.0336	0.0060	0.60	3.1E-08	720
Insomnia	rs9889282	17:50259142	A	-0.0419	0.0060	0.63	4.7E-12	1093

Insomnia	rs9931543	16:56128782	T	0.0478	0.0070	0.74	1.1E-12	1169
Insomnia	rs9964420	18:56824041	A	0.0354	0.0070	0.29	4.5E-08	688
Sports	rs10946808	6:26233387	G	0.0300	0.0078	0.38	9.9E-10	149
Sports	rs159544	5:60489247	G	0.0300	0.0078	0.39	1.3E-09	150
Sports	rs166840	17:19799698	G	0.0300	0.0071	0.63	3.1E-11	147
Sports	rs62253088	3:85400801	T	0.0490	0.0082	0.31	1.0E-19	363
Sports	rs75930676	14:71826547	C	0.0730	0.0193	0.03	2.0E-09	126
MVPA	rs1160545	2:100832269	T	0.0249	0.0041	0.40	1.7E-09	196
MVPA	rs12357890	10:99762693	A	0.0225	0.0041	0.51	4.8E-08	167
MVPA	rs13201721	6:141799534	T	0.0255	0.0040	0.73	1.8E-10	169
MVPA	rs1625595	11:66078129	T	-0.0213	0.0032	0.46	1.9E-11	149
MVPA	rs1691471	3:85011013	T	0.0379	0.0042	0.30	1.7E-19	400
MVPA	rs2290338	3:85362101	T	-0.0257	0.0039	0.28	7.3E-11	176
MVPA	rs2668196	3:165502709	A	-0.0227	0.0040	0.18	2.1E-08	99
MVPA	rs336620	3:18628793	C	0.0243	0.0044	0.35	4.0E-08	177
MVPA	rs385301	17:19806828	T	-0.0284	0.0047	0.23	1.6E-09	187
MVPA	rs4546329	5:60589739	T	-0.0181	0.0032	0.49	1.4E-08	108
MVPA	rs4865512	5:50661601	A	0.0240	0.0042	0.62	7.7E-09	179
MVPA	rs568546	11:107321156	T	0.0237	0.0041	0.51	5.9E-09	186
MVPA	rs6427178	1:169095082	A	0.0229	0.0041	0.50	1.7E-08	173
MVPA	rs7613360	3:49916710	T	-0.0247	0.0042	0.31	2.8E-09	172
MVPA	rs9903845	17:50291181	A	-0.0200	0.0034	0.26	6.1E-09	101
VPA	rs1248860	3:85015779	A	0.0410	0.0086	0.47	1.1E-13	219
VPA	rs13243553	7:133506955	G	0.0390	0.0094	0.62	9.0E-11	188
VPA	rs2764261	6:108927842	A	0.0390	0.0091	0.42	2.0E-11	194
VPA	rs328902	7:35020843	T	0.0410	0.0104	0.30	5.5E-10	183
VPA	rs3781411	10:126715436	C	0.0580	0.0145	0.84	3.0E-10	236

Sedentary behaviour	rs10041724	5:124273520	T	0.0180	0.0030	0.81	3.9E-11	40
Sedentary behaviour	rs10054327	5:147871137	G	0.0170	0.0020	0.61	3.4E-15	56
Sedentary behaviour	rs10145592	14:94287215	C	-0.0150	0.0020	0.38	1.8E-11	43
Sedentary behaviour	rs10189857	2:60713235	A	-0.0210	0.0020	0.53	6.2E-21	90
Sedentary behaviour	rs1022785	14:29571653	G	0.0180	0.0030	0.14	7.1E-09	33
Sedentary behaviour	rs10234444	7:41828527	G	0.0160	0.0030	0.86	2.9E-08	25
Sedentary behaviour	rs10246289	7:115544763	A	0.0190	0.0030	0.14	1.7E-08	35
Sedentary behaviour	rs1031423	5:93276883	T	-0.0190	0.0030	0.15	1.8E-12	38
Sedentary behaviour	rs10427502	21:40654840	G	0.0140	0.0020	0.68	8.6E-10	35
Sedentary behaviour	rs10737620	1:193057971	T	0.0140	0.0020	0.33	2.6E-09	35
Sedentary behaviour	rs10771746	12:30791864	C	-0.0140	0.0020	0.69	2.5E-09	34
Sedentary behaviour	rs10772643	12:13415288	C	0.0250	0.0040	0.16	1.3E-12	69
Sedentary behaviour	rs10876864	12:56401085	G	-0.0130	0.0020	0.41	1.0E-09	34
Sedentary behaviour	rs10890123	1:74802123	C	0.0140	0.0030	0.79	3.3E-08	26
Sedentary behaviour	rs10932837	2:221044785	C	-0.0130	0.0020	0.46	1.3E-09	34
Sedentary behaviour	rs10940659	5:59360341	A	-0.0130	0.0020	0.54	1.5E-08	34
Sedentary behaviour	rs10994943	10:63591413	T	0.0130	0.0020	0.62	3.1E-09	33
Sedentary behaviour	rs11020045	11:92487907	A	-0.0130	0.0020	0.66	1.6E-08	31
Sedentary behaviour	rs11130793	3:60884659	C	0.0130	0.0020	0.58	5.0E-09	34
Sedentary behaviour	rs11201422	10:86924483	T	0.0130	0.0020	0.70	3.4E-08	29
Sedentary behaviour	rs11218575	11:122154576	C	0.0150	0.0020	0.64	2.1E-12	43
Sedentary behaviour	rs11245482	10:126733546	T	-0.0130	0.0020	0.64	2.6E-09	32
Sedentary behaviour	rs114328297	1:91190854	T	0.0140	0.0030	0.81	4.4E-08	25
Sedentary behaviour	rs114600294	3:181419367	G	-0.0160	0.0030	0.82	7.9E-10	31
Sedentary behaviour	rs1156541	18:39952989	C	0.0150	0.0030	0.27	2.8E-08	36
Sedentary behaviour	rs11654952	17:4813799	T	-0.0170	0.0030	0.86	4.3E-08	29
Sedentary behaviour	rs11657730	17:79372489	C	0.0130	0.0020	0.65	1.1E-08	32
Sedentary behaviour	rs11689199	2:100820421	A	0.0190	0.0020	0.61	5.5E-17	71

Sedentary behaviour	rs11714337	3:71582521	G	0.0140	0.0020	0.61	4.7E-11	38
Sedentary behaviour	rs11763734	7:126400910	A	-0.0130	0.0020	0.50	8.2E-09	35
Sedentary behaviour	rs11810109	1:44186812	A	0.0160	0.0020	0.69	5.4E-12	45
Sedentary behaviour	rs12105701	2:41737200	C	-0.0130	0.0020	0.33	5.4E-09	30
Sedentary behaviour	rs12272012	11:84861286	G	-0.0300	0.0050	0.97	1.9E-08	24
Sedentary behaviour	rs12289262	11:12894758	C	-0.0140	0.0020	0.77	2.0E-08	28
Sedentary behaviour	rs1243182	10:21916728	C	-0.0190	0.0020	0.70	2.0E-15	62
Sedentary behaviour	rs12476388	2:116330983	C	0.0130	0.0020	0.67	2.1E-08	30
Sedentary behaviour	rs12491503	3:165706855	G	-0.0140	0.0020	0.69	5.5E-10	34
Sedentary behaviour	rs12541615	8:118870151	T	-0.0180	0.0030	0.75	3.9E-10	50
Sedentary behaviour	rs12554512	9:23352293	T	0.0210	0.0020	0.58	3.8E-21	88
Sedentary behaviour	rs12725114	1:62432641	G	0.0150	0.0030	0.79	1.6E-08	31
Sedentary behaviour	rs1278847	1:110037483	C	0.0160	0.0020	0.70	1.2E-11	44
Sedentary behaviour	rs13029509	2:215374209	G	-0.0180	0.0020	0.57	2.0E-17	65
Sedentary behaviour	rs13107325	4:103188709	C	-0.0290	0.0040	0.95	1.5E-12	30
Sedentary behaviour	rs138256022	3:11603090	C	-0.0310	0.0060	0.97	2.9E-08	24
Sedentary behaviour	rs141184308	9:86463339	A	0.0430	0.0080	0.99	2.6E-08	20
Sedentary behaviour	rs1421334	8:30865733	A	0.0170	0.0020	0.44	2.2E-15	58
Sedentary behaviour	rs1451533	2:105466005	G	-0.0160	0.0020	0.67	6.7E-12	46
Sedentary behaviour	rs17207890	11:95490754	G	0.0160	0.0020	0.73	1.1E-16	41
Sedentary behaviour	rs17379561	1:98340139	A	-0.0260	0.0030	0.80	7.0E-10	87
Sedentary behaviour	rs17512836	18:53194961	T	0.0420	0.0070	0.97	3.1E-09	40
Sedentary behaviour	rs17727474	10:127182959	C	0.0180	0.0030	0.82	1.4E-13	39
Sedentary behaviour	rs17789218	6:100600097	T	0.0190	0.0030	0.79	8.6E-12	49
Sedentary behaviour	rs2034768	3:93986371	A	0.0150	0.0020	0.46	5.9E-09	46
Sedentary behaviour	rs2045147	10:56660437	A	0.0130	0.0020	0.42	1.5E-10	34
Sedentary behaviour	rs2073869	9:135763816	C	0.0190	0.0030	0.75	1.3E-08	55
Sedentary behaviour	rs2092829	22:31780514	G	0.0140	0.0020	0.72	1.1E-08	32

Sedentary behaviour	rs2164744	12:109872039	T	-0.0130	0.0020	0.65	4.6E-09	31
Sedentary behaviour	rs2173650	12:117525235	G	0.0180	0.0030	0.85	3.0E-09	34
Sedentary behaviour	rs2184364	6:143183596	A	0.0160	0.0030	0.78	6.8E-12	36
Sedentary behaviour	rs2447098	17:2277720	C	-0.0150	0.0020	0.45	5.0E-10	46
Sedentary behaviour	rs2460	15:53073084	G	-0.0150	0.0030	0.78	2.9E-10	32
Sedentary behaviour	rs2584597	17:61941284	T	0.0150	0.0020	0.74	2.9E-14	36
Sedentary behaviour	rs2616830	9:1721385	G	0.0170	0.0020	0.42	3.2E-15	57
Sedentary behaviour	rs262890	5:62930015	A	-0.0190	0.0020	0.65	1.5E-08	68
Sedentary behaviour	rs2717559	8:143882420	A	0.0120	0.0020	0.56	1.2E-08	29
Sedentary behaviour	rs2787374	9:103054951	T	0.0130	0.0020	0.48	2.7E-10	34
Sedentary behaviour	rs303753	18:21074922	G	-0.0150	0.0020	0.66	1.4E-09	41
Sedentary behaviour	rs34864022	9:22609110	A	-0.0260	0.0040	0.94	3.0E-08	29
Sedentary behaviour	rs35574015	16:15146061	T	-0.0130	0.0020	0.69	5.5E-16	29
Sedentary behaviour	rs374722	2:147839830	G	0.0250	0.0030	0.18	4.8E-12	74
Sedentary behaviour	rs3754970	2:162091836	T	-0.0150	0.0020	0.54	3.2E-33	46
Sedentary behaviour	rs3796386	3:49899795	G	-0.0260	0.0020	0.66	2.8E-09	124
Sedentary behaviour	rs405797	14:26972834	T	-0.0150	0.0030	0.24	7.3E-09	34
Sedentary behaviour	rs42210	5:166408788	G	-0.0140	0.0020	0.34	2.1E-08	36
Sedentary behaviour	rs4334769	4:130245233	G	0.0120	0.0020	0.47	6.7E-09	29
Sedentary behaviour	rs4382592	9:134870755	T	0.0140	0.0020	0.30	5.0E-10	34
Sedentary behaviour	rs4523073	6:67550288	A	-0.0140	0.0020	0.64	1.6E-13	37
Sedentary behaviour	rs4577309	2:191288833	A	0.0160	0.0020	0.53	2.9E-08	52
Sedentary behaviour	rs4675246	2:202864487	G	-0.0150	0.0030	0.74	4.7E-09	35
Sedentary behaviour	rs4775373	15:61476936	T	0.0130	0.0020	0.34	1.3E-12	31
Sedentary behaviour	rs4845364	1:154141908	A	-0.0150	0.0020	0.45	1.4E-08	46
Sedentary behaviour	rs4937842	11:133734542	G	-0.0130	0.0020	0.61	1.0E-09	33
Sedentary behaviour	rs4973576	2:233806771	C	-0.0150	0.0020	0.29	2.1E-09	38
Sedentary behaviour	rs55700114	20:43717080	G	-0.0140	0.0020	0.64	2.4E-09	37

Sedentary behaviour	rs55909997	1:107581039	G	-0.0140	0.0020	0.67	3.8E-10	36
Sedentary behaviour	rs56103247	20:62483184	C	0.0300	0.0050	0.96	3.4E-10	31
Sedentary behaviour	rs56858768	13:86511730	G	-0.0150	0.0020	0.65	4.3E-09	42
Sedentary behaviour	rs57585211	5:107197725	T	-0.0170	0.0030	0.84	3.1E-13	33
Sedentary behaviour	rs6131281	20:11891724	C	0.0160	0.0020	0.60	1.3E-09	50
Sedentary behaviour	rs6141814	20:31368960	C	-0.0140	0.0020	0.61	7.8E-10	38
Sedentary behaviour	rs62379379	5:141082015	G	-0.0260	0.0040	0.91	7.0E-10	44
Sedentary behaviour	rs62641636	2:68425427	A	0.0140	0.0020	0.66	1.8E-09	36
Sedentary behaviour	rs6472942	8:76827190	T	-0.0130	0.0020	0.60	2.2E-11	33
Sedentary behaviour	rs6673341	1:184655649	T	-0.0150	0.0020	0.43	7.9E-12	45
Sedentary behaviour	rs66852340	4:3311070	C	-0.0180	0.0030	0.77	1.7E-10	47
Sedentary behaviour	rs6721975	2:5832667	T	-0.0170	0.0030	0.29	1.7E-13	49
Sedentary behaviour	rs6797840	3:85656569	A	-0.0160	0.0020	0.48	4.9E-15	52
Sedentary behaviour	rs6825241	4:152543783	C	-0.0170	0.0020	0.57	1.1E-10	58
Sedentary behaviour	rs6850494	4:82291771	A	-0.0140	0.0020	0.60	8.5E-18	39
Sedentary behaviour	rs6905544	6:98411631	A	-0.0190	0.0020	0.34	1.0E-09	66
Sedentary behaviour	rs6973656	7:77422583	A	-0.0140	0.0020	0.63	3.5E-08	37
Sedentary behaviour	rs6996198	8:65463442	C	-0.0160	0.0030	0.83	2.4E-09	30
Sedentary behaviour	rs7089973	10:116569565	C	-0.0130	0.0020	0.63	2.3E-08	32
Sedentary behaviour	rs7157001	14:99749484	A	-0.0140	0.0030	0.71	6.3E-10	33
Sedentary behaviour	rs71658797	1:77967507	T	-0.0200	0.0030	0.88	8.2E-13	34
Sedentary behaviour	rs7184800	16:53509131	G	0.0170	0.0020	0.73	3.4E-11	46
Sedentary behaviour	rs7189927	16:28913787	T	0.0150	0.0020	0.37	3.4E-10	43
Sedentary behaviour	rs7248205	19:10770305	C	0.0140	0.0020	0.41	4.5E-08	39
Sedentary behaviour	rs72671494	8:93195457	T	-0.0170	0.0030	0.88	3.0E-12	25
Sedentary behaviour	rs72781699	2:24277709	G	-0.0190	0.0030	0.83	4.9E-09	41
Sedentary behaviour	rs72828890	5:167806836	C	0.0190	0.0030	0.89	2.7E-13	30
Sedentary behaviour	rs72834698	6:26176517	G	0.0230	0.0030	0.87	2.7E-12	47

Sedentary behaviour	rs749671	16:31088347	G	0.0160	0.0020	0.62	2.8E-11	49
Sedentary behaviour	rs7564130	2:50606642	T	-0.0150	0.0020	0.60	1.7E-10	44
Sedentary behaviour	rs7693082	4:159857819	G	0.0150	0.0020	0.26	2.7E-09	36
Sedentary behaviour	rs7693703	4:118357405	G	0.0230	0.0040	0.89	1.9E-11	42
Sedentary behaviour	rs7700107	4:17880416	A	-0.0210	0.0030	0.88	3.6E-09	37
Sedentary behaviour	rs7716447	5:88800331	A	-0.0130	0.0020	0.76	1.5E-08	25
Sedentary behaviour	rs77215114	4:45187658	A	0.0240	0.0040	0.91	1.1E-10	39
Sedentary behaviour	rs7991062	13:100713194	C	-0.0180	0.0020	0.63	7.3E-14	62
Sedentary behaviour	rs801733	11:65934549	A	0.0170	0.0020	0.69	3.4E-08	51
Sedentary behaviour	rs8043253	15:41503730	C	-0.0120	0.0020	0.62	5.3E-10	28
Sedentary behaviour	rs8756	12:66359752	C	-0.0140	0.0020	0.49	1.5E-09	40
Sedentary behaviour	rs9471333	6:40362023	C	0.0130	0.0020	0.53	4.3E-11	34
Sedentary behaviour	rs9563168	13:54247827	G	0.0180	0.0030	0.78	3.4E-10	45
Sedentary behaviour	rs9569734	13:58319476	A	0.0190	0.0030	0.81	9.3E-19	45
Sedentary behaviour	rs9718104	6:166170539	T	-0.0410	0.0050	0.92	1.5E-08	96
Sedentary behaviour	rs973734	7:89387578	C	0.0170	0.0030	0.21	3.3E-09	39
Sedentary behaviour	rs9834970	3:36856030	T	0.0130	0.0020	0.55	4.1E-11	34
Sedentary behaviour	rs984409	1:67020440	G	-0.0150	0.0020	0.35	3.9E-12	42
Sedentary behaviour	rs9867121	3:114631548	C	0.0200	0.0030	0.81	4.5E-11	50
Sedentary behaviour	rs9902312	17:65070304	T	0.0150	0.0020	0.64	3.3E-14	42
Sedentary behaviour	rs9964724	18:35159124	C	0.0180	0.0020	0.30	6.6E-12	55
Leisure screen time	rs10041724	5:124273520	T	0.0249	0.0044	0.81	1.5E-08	125
Leisure screen time	rs1017550	10:63587683	A	0.0196	0.0035	0.67	2.9E-08	112
Leisure screen time	rs10189857	2:60713235	A	-0.0274	0.0035	0.53	7.8E-15	248
Leisure screen time	rs10222987	4:185946130	A	-0.0209	0.0036	0.63	9.0E-09	134
Leisure screen time	rs10253861	7:8110475	A	-0.0198	0.0035	0.49	1.5E-08	130
Leisure screen time	rs10400776	14:97326366	A	-0.0259	0.0044	0.23	3.4E-09	156
Leisure screen time	rs10469911	2:49989127	T	0.0204	0.0037	0.72	4.8E-08	111

Leisure screen time	rs10765775	11:95656362	A	-0.0223	0.0039	0.30	9.8E-09	139
Leisure screen time	rs10772643	12:13415288	T	-0.0385	0.0062	0.84	5.9E-10	265
Leisure screen time	rs10889193	1:61106174	A	0.0243	0.0039	0.64	4.9E-10	180
Leisure screen time	rs10947452	6:33803752	T	0.0220	0.0040	0.35	4.9E-08	145
Leisure screen time	rs11074658	16:10308335	T	-0.0239	0.0039	0.64	9.2E-10	174
Leisure screen time	rs114590429	2:166176789	A	0.0376	0.0060	0.15	3.0E-10	237
Leisure screen time	rs11587591	1:209762875	A	0.0219	0.0039	0.74	2.2E-08	121
Leisure screen time	rs1188887	6:139257866	T	0.0258	0.0041	0.63	2.5E-10	205
Leisure screen time	rs11972285	7:99025591	A	0.0384	0.0056	0.78	5.1E-12	333
Leisure screen time	rs12062845	1:98342685	A	0.0281	0.0042	0.27	2.4E-11	204
Leisure screen time	rs12188899	5:59670706	A	-0.0274	0.0048	0.80	1.1E-08	157
Leisure screen time	rs12206846	6:108238917	A	0.0199	0.0036	0.40	2.1E-08	126
Leisure screen time	rs12214364	6:67556372	T	-0.0197	0.0036	0.62	4.9E-08	120
Leisure screen time	rs12324720	15:64092140	A	-0.0271	0.0046	0.21	3.5E-09	162
Leisure screen time	rs12425850	12:123501972	T	-0.0236	0.0039	0.30	1.4E-09	154
Leisure screen time	rs12463321	19:37651855	A	-0.0317	0.0055	0.16	7.0E-09	177
Leisure screen time	rs12481468	20:43532438	T	-0.0225	0.0039	0.75	8.6E-09	124
Leisure screen time	rs12617870	2:193746283	T	0.0263	0.0035	0.57	6.6E-14	225
Leisure screen time	rs12678836	8:92690148	A	0.0232	0.0035	0.41	5.4E-11	173
Leisure screen time	rs12962050	18:35179808	A	-0.0234	0.0036	0.67	1.2E-10	161
Leisure screen time	rs12981974	19:19388071	C	0.0337	0.0058	0.18	8.6E-09	223
Leisure screen time	rs12992995	2:175197545	A	-0.0258	0.0043	0.26	2.1E-09	169
Leisure screen time	rs13017586	2:147847198	A	-0.0401	0.0054	0.83	8.3E-14	297
Leisure screen time	rs13107325	4:103188709	T	0.0404	0.0067	0.05	1.8E-09	95
Leisure screen time	rs13168806	5:120071604	A	0.0225	0.0040	0.40	1.5E-08	161
Leisure screen time	rs13188731	5:7387097	A	0.0218	0.0040	0.32	4.5E-08	137
Leisure screen time	rs13235840	7:133505091	A	-0.0313	0.0050	0.81	3.5E-10	197
Leisure screen time	rs13301354	9:139924637	T	-0.0211	0.0037	0.37	1.2E-08	138

Leisure screen time	rs1362910	8:30856464	A	0.0253	0.0035	0.40	1.0E-12	204
Leisure screen time	rs1375561	3:85658230	T	0.0232	0.0037	0.61	2.6E-10	169
Leisure screen time	rs1391954	11:88575965	T	0.0245	0.0040	0.49	1.5E-09	199
Leisure screen time	rs1483796	7:49871114	A	-0.0199	0.0036	0.37	2.1E-08	122
Leisure screen time	rs16825228	2:146346331	A	-0.0193	0.0035	0.52	4.1E-08	123
Leisure screen time	rs16896229	4:18002583	T	0.0300	0.0051	0.12	3.8E-09	124
Leisure screen time	rs17025214	3:88023541	T	-0.0221	0.0038	0.28	4.3E-09	131
Leisure screen time	rs1736523	1:3079885	C	0.0216	0.0039	0.55	2.8E-08	153
Leisure screen time	rs17621391	7:140176596	T	0.0243	0.0041	0.76	2.1E-09	143
Leisure screen time	rs17801257	20:58892520	A	-0.0292	0.0053	0.17	2.9E-08	159
Leisure screen time	rs1802669	10:21827796	A	0.0384	0.0041	0.32	2.4E-21	424
Leisure screen time	rs1837687	2:191238799	C	0.0222	0.0039	0.65	1.8E-08	148
Leisure screen time	rs1860337	17:60851559	T	-0.0253	0.0039	0.58	9.1E-11	206
Leisure screen time	rs1938450	1:68359482	A	-0.0226	0.0041	0.28	2.7E-08	135
Leisure screen time	rs1947066	5:161101615	A	0.0298	0.0044	0.80	8.5E-12	188
Leisure screen time	rs197439	1:112280990	A	-0.0259	0.0039	0.56	3.3E-11	219
Leisure screen time	rs1999065	9:120514574	T	0.0249	0.0037	0.37	1.2E-11	192
Leisure screen time	rs2344451	5:93000224	C	-0.0265	0.0047	0.15	1.6E-08	118
Leisure screen time	rs2473977	6:113454213	A	0.0195	0.0036	0.46	4.6E-08	125
Leisure screen time	rs249960	5:96164771	A	0.0298	0.0050	0.80	2.4E-09	186
Leisure screen time	rs2529484	7:111180444	C	0.0221	0.0036	0.38	1.4E-09	152
Leisure screen time	rs262890	5:62930015	A	-0.0344	0.0042	0.65	2.1E-16	359
Leisure screen time	rs2667382	15:83545977	A	0.0224	0.0039	0.46	6.7E-09	165
Leisure screen time	rs2783992	9:1722044	T	-0.0242	0.0038	0.58	3.4E-10	189
Leisure screen time	rs28458909	9:140257189	T	0.0364	0.0059	0.21	5.5E-10	291
Leisure screen time	rs2964252	5:152067929	A	-0.0235	0.0037	0.34	3.2E-10	164
Leisure screen time	rs2965216	19:13071082	A	0.0212	0.0039	0.49	4.6E-08	149
Leisure screen time	rs34811474	4:25408838	A	-0.0249	0.0045	0.22	4.5E-08	140

Leisure screen time	rs34864022	9:22609110	A	-0.0483	0.0078	0.94	4.7E-10	164
Leisure screen time	rs35039375	1:171516863	A	-0.0411	0.0066	0.92	6.3E-10	162
Leisure screen time	rs36079846	2:215367159	T	-0.0239	0.0038	0.57	4.5E-10	186
Leisure screen time	rs364789	5:77387439	A	0.0275	0.0044	0.80	2.8E-10	158
Leisure screen time	rs3759344	12:6862646	A	0.0452	0.0062	0.08	4.3E-13	202
Leisure screen time	rs3781412	10:126715154	A	-0.0208	0.0036	0.62	8.4E-09	135
Leisure screen time	rs3791033	1:44134077	T	0.0331	0.0041	0.67	3.7E-16	320
Leisure screen time	rs3853705	2:44261892	T	0.0228	0.0040	0.67	1.4E-08	151
Leisure screen time	rs396321	5:112113735	T	-0.0213	0.0035	0.50	1.3E-09	150
Leisure screen time	rs421151	8:73462574	A	-0.0360	0.0063	0.90	1.1E-08	158
Leisure screen time	rs4303732	2:100830040	T	0.0265	0.0035	0.60	5.4E-14	222
Leisure screen time	rs4311996	10:103735978	A	-0.0217	0.0039	0.39	3.6E-08	148
Leisure screen time	rs4416502	4:77030872	A	0.0291	0.0048	0.25	1.4E-09	208
Leisure screen time	rs4460001	4:130275243	A	-0.0193	0.0035	0.43	4.5E-08	121
Leisure screen time	rs4483592	11:65990439	T	0.0360	0.0052	0.18	4.0E-12	250
Leisure screen time	rs4551799	11:71416091	A	-0.0241	0.0039	0.32	6.9E-10	168
Leisure screen time	rs469565	22:29952437	T	-0.0259	0.0046	0.78	1.5E-08	154
Leisure screen time	rs4889530	16:31065918	A	-0.0253	0.0039	0.38	1.3E-10	199
Leisure screen time	rs558134	6:12693454	T	-0.0225	0.0036	0.42	5.0E-10	163
Leisure screen time	rs56151256	15:78024806	A	0.0285	0.0044	0.80	1.2E-10	175
Leisure screen time	rs566017137	2:157149797	C	-0.0656	0.0108	0.97	1.2E-09	181
Leisure screen time	rs57092155	7:53856368	T	-0.0268	0.0047	0.77	8.8E-09	171
Leisure screen time	rs58087899	1:1863026	A	-0.0222	0.0040	0.46	4.3E-08	162
Leisure screen time	rs58541850	6:166165563	A	0.0522	0.0082	0.07	1.7E-10	241
Leisure screen time	rs6010651	20:62418243	A	0.0235	0.0040	0.62	3.3E-09	173
Leisure screen time	rs60603116	2:217431561	A	-0.0226	0.0039	0.59	6.4E-09	163
Leisure screen time	rs6073637	20:43714051	A	-0.0225	0.0038	0.45	4.6E-09	166
Leisure screen time	rs6102913	20:41202958	T	-0.0193	0.0035	0.55	3.0E-08	122

Leisure screen time	rs61166637	4:140771814	C	0.0242	0.0043	0.75	1.5E-08	147
Leisure screen time	rs61813324	1:156049877	T	0.0310	0.0057	0.15	4.4E-08	162
Leisure screen time	rs62068672	16:89647015	T	0.0263	0.0048	0.73	4.1E-08	181
Leisure screen time	rs62134209	2:45093457	A	0.0538	0.0091	0.95	3.3E-09	167
Leisure screen time	rs62151809	2:104433256	T	0.0228	0.0039	0.44	3.9E-09	170
Leisure screen time	rs62244886	3:71587392	C	0.0239	0.0039	0.64	1.2E-09	175
Leisure screen time	rs6457816	6:35362848	T	-0.0414	0.0070	0.94	4.0E-09	130
Leisure screen time	rs6674314	1:243920895	A	0.0274	0.0049	0.78	2.3E-08	172
Leisure screen time	rs6685030	1:171805284	A	-0.0217	0.0035	0.45	5.3E-10	154
Leisure screen time	rs68049022	10:66407019	T	0.0314	0.0048	0.83	6.2E-11	186
Leisure screen time	rs6857	19:45392254	T	-0.0369	0.0047	0.18	5.8E-15	267
Leisure screen time	rs71658797	1:77967507	A	0.0373	0.0058	0.12	1.8E-10	192
Leisure screen time	rs7236799	18:41627516	A	0.0242	0.0041	0.31	4.9E-09	167
Leisure screen time	rs72671494	8:93195457	T	-0.0349	0.0056	0.88	5.7E-10	168
Leisure screen time	rs73405293	12:117522917	A	-0.0308	0.0054	0.15	1.3E-08	161
Leisure screen time	rs73420302	17:77768068	C	-0.0300	0.0051	0.25	3.0E-09	222
Leisure screen time	rs7430216	3:75201030	T	0.0250	0.0042	0.23	2.5E-09	149
Leisure screen time	rs7432837	3:93809151	T	-0.0235	0.0040	0.74	3.2E-09	142
Leisure screen time	rs743699	4:3305116	A	-0.0267	0.0044	0.73	1.2E-09	185
Leisure screen time	rs74996610	12:24075007	C	-0.0513	0.0093	0.95	3.1E-08	156
Leisure screen time	rs7579662	2:161915810	A	0.0216	0.0039	0.38	4.1E-08	145
Leisure screen time	rs7615206	3:49937505	T	-0.0345	0.0035	0.66	1.5E-22	354
Leisure screen time	rs76267866	3:70540347	A	-0.0297	0.0047	0.81	3.5E-10	182
Leisure screen time	rs7627290	3:165711001	A	-0.0231	0.0039	0.56	2.4E-09	174
Leisure screen time	rs76602404	7:50737835	T	-0.0266	0.0049	0.21	4.4E-08	157
Leisure screen time	rs78140587	18:41817809	A	0.0615	0.0099	0.97	5.8E-10	162
Leisure screen time	rs7821826	8:10769439	T	0.0207	0.0035	0.46	3.3E-09	141
Leisure screen time	rs78394231	6:107649123	T	-0.0381	0.0065	0.90	3.5E-09	176

Leisure screen time	rs7875078	9:14494845	A	0.0204	0.0035	0.51	7.4E-09	138
Leisure screen time	rs7969719	12:109883577	T	0.0271	0.0037	0.27	4.5E-13	192
Leisure screen time	rs7991062	13:100713194	C	-0.0335	0.0041	0.63	1.3E-16	347
Leisure screen time	rs841020	10:125409953	T	0.0269	0.0044	0.25	1.1E-09	180
Leisure screen time	rs892087	19:10794793	T	-0.0284	0.0040	0.64	1.0E-12	245
Leisure screen time	rs9278004	6:33319815	A	0.0394	0.0057	0.11	3.2E-12	195
Leisure screen time	rs9423279	10:125680419	C	0.0230	0.0041	0.40	2.5E-08	168
Leisure screen time	rs9513416	13:99055774	A	0.0281	0.0048	0.87	4.1E-09	119
Leisure screen time	rs9821299	3:83068067	A	0.0285	0.0048	0.19	3.6E-09	167
Leisure screen time	rs9867121	3:114631548	A	-0.0317	0.0050	0.19	2.0E-10	203

Supplemental Table 5. Sample overlap between used GWAS data sources.

	Overlapping	
**Trait**	**Circulating protein**	**CAD**
Circulating protein	/	0
Body mass index	0	0
Waist-to-hip ratio	0	0
Visceral adipose tissue	0	0
Waist circumference	0	4.9%
Smoking initiation	0	1.8%
Lifetime smoking index	0	0
Alcohol drinking	0	1.8%
Alcohol dependence	0	0
Coffee consumption	0	0
Caffeine consumption	0	0.4%
Sports	0	0
MVPA	0	0
VPA	0	0
Sedentary behavior	0	0
Leisure screen time	0	0
Sleep duration	0	0
Short sleep duration	0	0
Long sleep duration	0	0
Insomnia	0	0

Note: CAD, coronary artery disease; MVPA, moderate to vigorous physical activity; VPA, vigorous physical activity.

Supplemental Table 6. The bias and type 1 error rate caused by the sample overlap.

Exposure	Outcome	Overlap rate	Bias	Type 1 error rate
Waist circumference	CAD	4.9%	<0.002	0.05
Smoking initiation	CAD	1.8%	<0.001	0.05
Alcohol drinking	CAD	1.8%	<0.001	0.05
Caffeine consumption	CAD	0.4%	<0.001	0.05

Note: CAD, coronary artery disease; MVPA, moderate to vigorous physical activity; VPA, vigorous physical activity.

Supplemental Table 7. Posterior probability of H3 and H4 in colocalization analysis.

UniProt	Protein	PP3+PP4	PP4/(PP3+PP4)
Q8NBP7	PCSK9	1.00	1.00
Q9BY76	ANGPTL4	1.00	1.00
P37802	TAGLN2	1.00	1.00
P37802	TAGLN2	1.00	1.00
Q13308	PTK7	1.00	1.00
P21964	COMT	1.00	0.99
P08887	IL6R	1.00	0.99
P04114	APOB	1.00	0.98
P09871	C1S	1.00	0.96
P00736	C1R	1.00	0.96
P01160	NPPA	1.00	0.93
Q9NUM4	TMEM106B	1.00	0.93
P08670	VIM	0.95	0.96
Q99487	PAFAH2	0.98	0.94
P13497	BMP1	1.00	0.88
P22676	CALB2	1.00	0.87
Q9NRN7	AASDHPPT	0.94	0.89
Q6NW40	RGMB	0.91	0.92
P02751	FN1	0.96	0.87
P02751	FN1	0.96	0.87
P35625	TIMP3	0.94	0.89
P02751	FN1	0.96	0.87
P38936	CDKN1A	0.95	0.86
P36941	LTBR	0.92	0.88

Note: PP3, posterior probability of H3; PP4, posterior probability of H4.

Supplemental Table 8. Associations of genetically predicted obesity measures with CAD in sensitivity analyses.

Exposure	SNPs	MR method	OR	95% CI	P_{Effect}	$P_{Heterogeneity}$	$P_{Intercept}$
BMI	311	Weighted mode	1.42	(1.22-1.65)	8.6E-06		
		MR Egger	1.43	(1.22-1.68)	1.4E-05	<0.001	0.86
		Simple median	1.43	(1.33-1.53)	5.7E-22		
		Weighted median	1.39	(1.28-1.51)	4.6E-14		
WHR	292	Weighted mode	1.37	(1.06-1.78)	0.02		
		MR Egger	1.40	(1.07-1.84)	0.02	<0.001	0.72
		Simple median	1.46	(1.35-1.59)	5.5E-19		
		Weighted median	1.41	(1.29-1.55)	2.5E-14		
WC	46	Weighted mode	1.37	(1.18-1.59)	1.9E-04		
		MR Egger	1.73	(1.16-2.57)	0.01	<0.001	0.16
		Simple median	1.33	(1.18-1.50)	2.0E-06		
		Weighted median	1.38	(1.21-1.57)	2.2E-06		
VAT	219	Weighted mode	1.43	(1.22-1.66)	8.3E-06		
		MR Egger	1.32	(1.06-1.64)	0.01	<0.001	0.51
		Simple median	1.39	(1.29-1.49)	2.9E-18		
		Weighted median	1.43	(1.31-1.56)	9.5E-16		

Note: CAD, coronary artery disease; BMI, body mass index; WHR, waist-to-hip ratio; WC, waist circumference; VAT, visceral adipose tissue; OR, odds ratio; CI, confidence interval.

Supplemental Table 9. Associations of genetically predicted smoking, alcohol use and coffee consumption with CAD in sensitivity analyses.

Exposure	SNPs	MR method	OR	95% CI	P_{Effect}	$P_{Heterogeneity}$	$P_{Intercept}$
Smoking Initiation	313	Weighted mode	1.23	(1.04-1.45)	0.02		
		MR Egger	1.36	(1.09-1.71)	7.3E-03	<0.001	0.42
		Simple median	1.24	(1.17-1.32)	3.1E-12		
		Weighted median	1.24	(1.16-1.32)	1.1E-10		
Smoking Index	126	Weighted mode	1.56	(1.05-2.33)	0.03		
		MR Egger	1.18	(0.73-1.91)	0.51	<0.001	0.52
		Simple median	1.55	(1.36-1.78)	1.9E-10		
		Weighted median	1.42	(1.25-1.62)	7.6E-08		
Alcohol Drinking	96	Weighted mode	1.30	(1.07-1.57)	8.7E-03		
		MR Egger	1.22	(0.86-1.74)	0.26	<0.001	0.53
		Simple median	1.21	(1.00-1.46)	0.05		
		Weighted median	1.26	(1.03-1.53)	0.02		
Alcohol Dependence	3	Weighted mode	1.05	(1.00-1.10)	0.17		
		MR Egger	1.05	(0.99-1.12)	0.34	0.62	0.72
		Simple median	1.05	(0.99-1.11)	0.11		
		Weighted median	1.05	(1.00-1.10)	0.03		
Coffee consumption	12	Weighted mode	1.00	(1.00-1.01)	0.02		
		MR Egger	1.17	(0.79-1.72)	0.46	<0.001	0.75
		Simple median	1.00	(1.00-1.01)	0.24		
		Weighted median	1.13	(0.98-1.29)	0.09		

Note: CAD, coronary artery disease; OR, odds ratio; CI, confidence interval. The results for caffeine consumption with CAD using the weighted-median, MR-Egger regression, and weighted mode methods were not reported, due to the insufficient number of SNP.

Supplemental Table 10. Associations of genetically predicted sleep traits with CAD in sensitivity analyses.

Exposure	SNPs	MR method	OR	95% CI	P_{Effect}	$P_{Heterogeneity}$	$P_{Intercept}$
Sleep Duration	73	Weighted mode	0.73	(0.53-1.01)	0.06		
		MR Egger	0.76	(0.40-1.47)	0.42	<0.001	0.88
		Simple median	0.74	(0.63-0.89)	9.4E-04		
		Weighted median	0.74	(0.62-0.89)	1.1E-03		
Short Sleep Duration	26	Weighted mode	1.35	(1.04-1.75)	0.03		
		MR Egger	1.20	(0.72-1.99)	0.49	0.00	0.92
		Simple median	1.29	(1.14-1.47)	8.9E-05		
		Weighted median	1.25	(1.09-1.43)	1.0E-03		
Long Sleep Duration	7	Weighted mode	0.88	(0.74-1.05)	0.21		
		MR Egger	1.03	(0.74-1.43)	0.88	0.16	0.39
		Simple median	0.94	(0.80-1.10)	0.45		
		Weighted median	0.90	(0.78-1.04)	0.14		
Insomnia	208	Weighted mode	1.09	(0.99-1.20)	0.08		
		MR Egger	1.03	(0.91-1.18)	0.62	<0.001	0.45
		Simple median	1.09	(1.05-1.12)	3.6E-06		
		Weighted median	1.09	(1.05-1.13)	1.0E-05		

Note: CAD, coronary artery disease; OR, odds ratio; CI, confidence interval.

Supplemental Table 11. Associations of genetically predicted physical activity and sedentary behavior with CAD in sensitivity analyses.

Exposure	SNPs	MR method	OR	95% CI	P_{Effect}	$P_{Heterogeneity}$	$P_{Intercept}$
Sports	5	Weighted mode	0.92	(0.72-1.17)	0.52		
		MR Egger	0.98	(0.56-1.73)	0.96	0.49	0.67
		Simple median	0.88	(0.70-1.11)	0.28		
		Weighted median	0.90	(0.74-1.11)	0.33		
MVPA	15	Weighted mode	0.74	(0.55-1.01)	0.08		
		MR Egger	1.51	(0.43-5.37)	0.53	<0.001	0.28
		Simple median	0.67	(0.53-0.84)	7.2E-04		
		Weighted median	0.78	(0.63-0.96)	0.02		
VPA	5	Weighted mode	0.89	(0.71-1.12)	0.39		
		MR Egger	0.49	(0.19-1.24)	0.23	0.80	0.30
		Simple median	0.89	(0.74-1.07)	0.22		
		Weighted median	0.89	(0.75-1.06)	0.19		
Sedentary behavior	133	Weighted mode	1.40	(1.06-1.84)	0.02		
		MR Egger	1.89	(1.18-3.01)	8.5E-03	<0.001	0.08
		Simple median	1.35	(1.20-1.52)	7.0E-07		
		Weighted median	1.35	(1.20-1.51)	3.5E-07		
Leisure screen time	129	Weighted mode	1.06	(0.86-1.30)	0.60		
		MR Egger	0.76	(0.56-1.03)	0.08	<0.001	0.004
		Simple median	1.16	(1.08-1.25)	3.6E-05		
		Weighted median	1.16	(1.08-1.25)	6.9E-05		

Note: CAD, coronary artery disease; MVPA, moderate to vigorous physical activity; VPA, vigorous physical activity; OR, odds ratio; CI, confidence interval.

Supplemental Table 12. Associations of genetically predicted obesity and lifestyle factors with CAD in MR-PRESSO Analyses.

Trait	OR	95% CI	*P*	Outlier	$P_{distrotion}$
BMI	1.47	(1.39, 1.55)	<0.001	14	0.20
WHR	1.50	(1.40, 1.61)	<0.001	22	0.55
WC	1.34	(1.24, 1.45)	<0.001	9	0.59
VAT	1.45	(1.36, 1.54)	<0.001	4	0.49
Smoking initiation	1.25	(1.19, 1.31)	<0.001	5	0.99
Lifetime smoking index	1.37	(1.23, 1.53)	<0.001	2	0.99
Alcohol drinking	1.13	(0.96, 1.33)	0.146	2	0.88
Coffee consumption	1.22	(1.08, 1.37)	0.012	2	0.82
Sports	NA	NA	NA	NA	NA
MVPA	0.69	(0.57, 0.84)	0.003	1	0.57
VPA	NA	NA	NA	NA	NA
Sedentary behavior	1.28	(1.16, 1.40)	<0.001	3	0.75
Leisure screen time	1.19	(1.12, 1.26)	<0.001	2	0.39
Sleep duration	0.79	(0.68, 0.91)	0.002	4	0.78
Long sleep duration	NA	NA	NA	NA	NA
Short sleep duration	NA	NA	NA	NA	NA
Insomnia	1.09	(1.06, 1.12)	<0.001	5	0.94

Note: BMI, body mass index; WHR, waist-to-hip ratio; WC, waist circumference; VAT, visceral adipose tissue; MVPA, moderate to vigorous physical activity; VPA, vigorous physical activity; OR, odds ratio; CI, confidence interval. $P_{distrotion}$, the *p* value of MR-PRESSO distrotion test; NA, no outlier was found. The MR-PRESSO method was not available for caffeine consumption and alcohol dependence, due to the limited number of SNP.

Supplemental Table 13. Associations of genetically predicted obesity measures with levels of MAP1LC3A.

Exposure	nsnp	MR method	beta	se	pval
BMI	299	Weighted mode	0.195	0.075	0.010
		MR Egger	0.150	0.087	0.086
		IVW	0.112	0.035	0.001
		Simple median	0.097	0.047	0.038
		Weighted median	0.165	0.052	0.001
VAT	212	Weighted mode	0.192	0.083	0.023
		MR Egger	0.272	0.098	0.006
		IVW	0.067	0.031	0.030
		Simple median	0.096	0.046	0.035
		Weighted median	0.161	0.050	0.001

Note: nsnp, number of single nucleotide polymorphisms; se, standard error; pval, *p* value; BMI, body mass index; WHR, waist-to-hip ratio; WC, waist circumference; VAT, visceral adipose tissue; IVW, Inverse variance weighted analysis.

Supplemental Table 14. Associations of genetically predicted obesity measures with levels of ANGPTL4.

Exposure	nsnp	MR method	beta	se	pval
BMI	299	Weighted mode	0.184	0.077	0.017
		MR Egger	0.221	0.076	0.004
		IVW	0.108	0.029	<0.001
		Simple median	0.091	0.046	0.046
		Weighted median	0.171	0.057	0.003
WHR	280	Weighted mode	0.135	0.087	0.122
		MR Egger	0.075	0.087	0.391
		IVW	0.081	0.033	0.014
		Simple median	0.111	0.049	0.024
		Weighted median	0.084	0.062	0.175
VAT	212	Weighted mode	0.164	0.092	0.075
		MR Egger	0.318	0.098	0.001
		IVW	0.098	0.031	0.001
		Simple median	0.137	0.045	0.002
		Weighted median	0.159	0.050	0.002

Note: nsnp, number of single nucleotide polymorphisms; se, standard error; pval, *p* value; BMI, body mass index; WHR, waist-to-hip ratio; WC, waist circumference; VAT, visceral adipose tissue; IVW, Inverse variance weighted analysis.

Supplemental Table 15. Associations of genetically predicted obesity measures with levels of RPS6KA1.

Exposure	nsnp	MR method	beta	se	pval
BMI	299	Weighted mode	0.198	0.067	0.003
		MR Egger	0.160	0.075	0.035
		IVW	0.158	0.030	<0.001
		Simple median	0.183	0.043	<0.001
		Weighted median	0.191	0.048	<0.001
WHR	280	Weighted mode	0.073	0.085	0.397
		MR Egger	0.103	0.081	0.205
		IVW	0.166	0.030	<0.001
		Simple median	0.158	0.043	<0.001
		Weighted median	0.110	0.048	0.022
WC	45	Weighted mode	0.199	0.073	0.010
		MR Egger	0.337	0.121	0.008
		IVW	0.091	0.042	0.029
		Simple median	0.126	0.066	0.055
		Weighted median	0.192	0.067	0.004
VAT	212	Weighted mode	0.192	0.076	0.013
		MR Egger	0.282	0.085	0.001
		IVW	0.136	0.027	<0.001
		Simple median	0.123	0.042	0.004
		Weighted median	0.176	0.045	<0.001

Note: nsnp, number of single nucleotide polymorphisms; se, standard error; pval, *p* value; BMI, body mass index; WHR, waist-to-hip ratio; WC, waist circumference; VAT, visceral adipose tissue; IVW, Inverse variance weighted analysis.

Supplemental Table 16. Associations of genetically predicted obesity measures with levels of PCSK9.

Exposure	nsnp	MR method	beta	se	pval
BMI	299	Weighted mode	0.188	0.084	0.025
		MR Egger	0.220	0.084	0.010
		IVW	0.141	0.033	<0.001
		Simple median	0.163	0.047	<0.001
		Weighted median	0.185	0.056	0.001
WHR	280	Weighted mode	0.151	0.092	0.103
		MR Egger	0.030	0.109	0.781
		IVW	0.151	0.041	<0.001
		Simple median	0.137	0.053	0.010
		Weighted median	0.177	0.069	0.011
WC	45	Weighted mode	0.201	0.095	0.039
		MR Egger	0.235	0.175	0.186
		IVW	0.124	0.048	0.010
		Simple median	0.118	0.076	0.120
		Weighted median	0.224	0.079	0.005
VAT	212	Weighted mode	0.245	0.104	0.019
		MR Egger	0.422	0.104	<0.001
		IVW	0.166	0.031	<0.001
		Simple median	0.155	0.047	0.001
		Weighted median	0.197	0.053	<0.001

Note: nsnp, number of single nucleotide polymorphisms; se, standard error; pval, *p* value; BMI, body mass index; WHR, waist-to-hip ratio; WC, waist circumference; VAT, visceral adipose tissue; IVW, Inverse variance weighted analysis.

Supplemental Table 17. Associations of genetically predicted obesity measures with levels of ITPKA.

Exposure	nsnp	MR method	beta	se	pval
BMI	299	Weighted mode	0.179	0.076	0.020
		MR Egger	0.169	0.075	0.025
		IVW	0.113	0.030	<0.001
		Simple median	0.109	0.045	0.015
		Weighted median	0.134	0.046	0.003
WHR	280	Weighted mode	0.151	0.094	0.112
		MR Egger	0.112	0.087	0.197
		IVW	0.113	0.033	0.001
		Simple median	0.134	0.050	0.007
		Weighted median	0.162	0.061	0.008
VAT	212	Weighted mode	0.160	0.092	0.086
		MR Egger	0.296	0.095	0.002
		IVW	0.097	0.030	0.001
		Simple median	0.094	0.046	0.039
		Weighted median	0.123	0.045	0.007

Note: nsnp, number of single nucleotide polymorphisms; se, standard error; pval, *p* value; BMI, body mass index; WHR, waist-to-hip ratio; WC, waist circumference; VAT, visceral adipose tissue; IVW, Inverse variance weighted analysis.

Supplemental Table 18. Associations of genetically predicted physical activity with levels of circulating proteins.

Exposure	Protein	nsnp	MR method	beta	se	pval
Sports	LGALS2	5	Weighted mode	0.226	0.165	0.244
			MR Egger	0.271	0.354	0.501
			IVW	0.214	0.106	0.043
			Simple median	0.222	0.133	0.096
			Weighted median	0.228	0.128	0.075
VPA	CNP	5	Weighted mode	0.300	0.165	0.142
			MR Egger	-0.199	0.668	0.785
			IVW	0.234	0.094	0.012
			Simple median	0.285	0.125	0.023
			Weighted median	0.264	0.124	0.033

Note: nsnp, number of single nucleotide polymorphisms; se, standard error; pval, *p* value; VPA, vigorous physical activity; IVW, Inverse variance weighted analysis.

Supplemental Table 19. Associations of genetically predicted sedentary behavior with levels of circulating proteins.

Exposure	Protein	nsnp	MR method	beta	se	pval
SB	PCSK9	123	Weighted mode	0.167	0.174	0.338
			MR Egger	0.131	0.240	0.585
			IVW	0.145	0.052	0.005
			Simple median	0.114	0.074	0.120
			Weighted median	0.112	0.071	0.115
SB	MST1	123	Weighted mode	-0.024	0.126	0.849
			MR Egger	7.161	1.496	<0.001
			IVW	0.911	0.037	<0.001
			Simple median	-0.024	0.056	0.675
			Weighted median	-0.011	0.056	0.849
LST	RPS6KA1	122	Weighted mode	0.148	0.123	0.228
			MR Egger	0.195	0.132	0.143
			IVW	0.084	0.028	0.003
			Simple median	0.099	0.042	0.019
			Weighted median	0.100	0.042	0.017
LST	MST1	122	Weighted mode	0.072	0.075	0.337
			MR Egger	2.092	0.885	0.020
			IVW	0.460	0.023	<0.001
			Simple median	0.051	0.035	0.145
			Weighted median	0.055	0.033	0.091

Note: nsnp, number of single nucleotide polymorphisms; se, standard error; pval, *p* value; SB, sedentary behavior; LST, leisure screen time; IVW, Inverse variance weighted analysis.

Supplemental Table 20. Associations of genetically predicted smoking with levels of circulating proteins.

Exposure	Protein	nsnp	MR method	beta	se	pval
Smoking initiation	RGMB	303	Weighted mode	-0.216	0.114	0.059
			MR Egger	0.066	0.122	0.588
			IVW	-0.121	0.029	<0.001
			Simple median	-0.173	0.041	<0.001
			Weighted median	-0.161	0.040	<0.001
Smoking initiation	PCSK9	303	Weighted mode	-0.034	0.122	0.780
			MR Egger	0.106	0.120	0.379
			IVW	0.077	0.029	0.007
			Simple median	0.068	0.040	0.086
			Weighted median	0.050	0.040	0.209
Smoking index	ITPKA	122	Weighted mode	0.229	0.199	0.252
			MR Egger	-0.176	0.208	0.401
			IVW	0.142	0.054	0.009
			Simple median	0.217	0.078	0.006
			Weighted median	0.186	0.081	0.021
Smoking index	PCSK9	122	Weighted mode	0.115	0.206	0.576
			MR Egger	-0.269	0.221	0.226
			IVW	0.168	0.058	0.004
			Simple median	0.170	0.080	0.034
			Weighted median	0.112	0.083	0.179
Smoking index	RPS6KA1	122	Weighted mode	-0.083	0.191	0.664
			MR Egger	-0.041	0.210	0.846
			IVW	0.162	0.055	0.003
			Simple median	0.142	0.074	0.056
			Weighted median	0.089	0.075	0.232

Note: nsnp, number of single nucleotide polymorphisms; se, standard error; pval, *p* value; IVW, Inverse variance weighted analysis.

Supplemental Table 21. Associations of genetically predicted coffee and caffeine consumption with levels of circulating proteins.

Exposure	Protein	nsnp	MR method	beta	se	pval
Coffee consumption	MAP1LC3A	12	Weighted mode	0.084	0.072	0.270
			MR Egger	0.017	0.115	0.883
			IVW	0.119	0.060	0.047
			Simple median	0.156	0.100	0.121
			Weighted median	0.075	0.077	0.328
Coffee consumption	FAS	12	Weighted mode	0.186	0.071	0.023
			MR Egger	0.075	0.221	0.741
			IVW	0.324	0.060	<0.001
			Simple median	0.412	0.143	0.004
			Weighted median	0.180	0.078	0.022
Caffeine consumption	APOB	2	IVW	0.089	0.006	<0.001
Caffeine consumption	VIM	2	IVW	-0.036	0.005	<0.001
Caffeine consumption	FAS	2	IVW	0.078	0.001	<0.001

Note: nsnp, number of single nucleotide polymorphisms; se, standard error; pval, *p* value; IVW, Inverse variance weighted analysis. The associations of caffeine consumption with APOB, VIM, and FAS were only analyzed by the IVW method due to the limited number of SNPs (<3).

Supplemental Table 22. The estimates of the indirect effect and proportion mediated.

Exposure	Mediator	Indirect effect	Proportion mediated	95% CI
BMI	MAP1LC3A	0.057	16.5%	(4.3%, 28.6%)
BMI	ANGPTL4	0.036	10.3%	(3.6%, 16.9%)
BMI	RPS6KA1	0.035	10.1%	(3.4%, 16.7%)
BMI	PCSK9	0.035	10.0%	(5.0%, 14.9%)
BMI	ITPKA	0.034	9.8%	(2.4%, 17.3%)
BMI	AGER	0.021	6.0%	(2.5%, 9.4%)
BMI	RGMB	0.020	5.7%	(1.6%, 9.8%)
BMI	C1R	0.013	3.7%	(1.7%, 5.8%)
BMI	FAS	0.011	3.1%	(0.8%, 5.3%)
BMI	MST1	0.010	2.9%	(0.5%, 5.4%)
BMI	C1S	0.008	2.4%	(1.1%, 3.7%)
BMI	GCA	0.008	2.4%	(0.5%, 4.3%)
BMI	APOA5	0.006	1.7%	(0.6%, 2.9%)
WHR	PCSK9	0.037	9.6%	(4.2%, 15.0%)
WHR	RPS6KA1	0.037	9.6%	(3.3%, 15.8%)
WHR	ITPKA	0.034	8.8%	(1.8%, 15.9%)
WHR	ANGPTL4	0.027	6.9%	(0.8%, 13.1%)
WHR	AGER	0.017	4.5%	(1.5%, 7.5%)
WHR	RGMB	0.017	4.4%	(0.7%, 8.1%)
WHR	AGT	0.014	3.8%	(0.8%, 6.7%)
WHR	APOA5	0.012	3.1%	(1.5%, 4.6%)

WHR	C1R	0.007	1.8%	(0.2%, 3.4%)
WHR	FAS	0.007	1.7%	(0.1%, 3.4%)
WHR	C1S	0.006	1.7%	(0.6%, 2.7%)
WHR	GCA	0.006	1.6%	(0.1%, 3.2%)
WHR	LAMC2	0.003	0.7%	(0.0%, 1.4%)
WC	AGER	0.028	10.1%	(4.0%, 16.3%)
WC	RGMB	0.020	7.2%	(0.9%, 13.5%)
WC	C1S	0.006	2.3%	(0.3%, 4.4%)
VAT	PCSK9	0.041	11.8%	(6.6%, 17.0%)
VAT	PTK7	0.037	10.6%	(2.9%, 18.4%)
VAT	ANGPTL4	0.032	9.4%	(2.4%, 16.4%)
VAT	RPS6KA1	0.030	8.8%	(2.8%, 14.7%)
VAT	ITPKA	0.029	8.5%	(1.4%, 15.6%)
VAT	AGER	0.017	5.0%	(1.9%, 8.1%)
VAT	C1R	0.015	4.4%	(2.1%, 6.7%)
VAT	RGMB	0.013	3.7%	(0.2%, 7.2%)
VAT	FAS	0.012	3.5%	(1.0%, 6.0%)
VAT	MST1	0.012	3.4%	(0.5%, 6.3%)
VAT	C1S	0.008	2.3%	(1.1%, 3.6%)
VAT	GCA	0.007	2.0%	(0.3%, 3.8%)
VAT	APOA5	0.007	1.9%	(0.6%, 3.2%)
VAT	LAMC2	0.003	1.0%	(0.2%, 1.8%)
Smoking Initiation	RGMB	0.020	9.0%	(2.8%, 15.2%)
Smoking Initiation	PCSK9	0.019	8.5%	(2.1%, 14.9%)
Smoking Initiation	AGER	0.008	3.7%	(0.7%, 6.7%)

Smoking Initiation	MST1	0.007	3.2%	(0.1%, 6.3%)
Smoking Index	ITPKA	0.043	13.4%	(0.8%, 26.0%)
Smoking Index	PCSK9	0.041	12.9%	(3.8%, 22.0%)
Smoking Index	RPS6KA1	0.036	11.3%	(1.6%, 21.0%)
Smoking Index	C1S	0.009	2.7%	(0.7%, 4.7%)
Alcohol Dependence	ARG1	0.010	25.0%	(1.8%, 48.1%)
Alcohol Dependence	FN1	0.010	24.8%	(1.2%, 48.5%)
Alcohol Dependence	TIMP3	0.001	3.0%	(1.4%, 4.6%)
Coffee consumption	MAP1LC3A	0.001	29.0%	(2.5%, 55.4%)
Coffee consumption	FAS	0.000	10.0%	(0.6%, 19.4%)
Caffeine consumption	APOB	0.042	33.5%	(20.5%, 46.6%)
Caffeine consumption	VIM	0.012	9.9%	(4.7%, 15.2%)
Caffeine consumption	FAS	0.005	4.1%	(1.6%, 6.5%)
Caffeine consumption	THSD1	0.004	3.5%	(1.4%, 5.6%)
Caffeine consumption	ANGPTL1	0.004	3.5%	(0.7%, 6.2%)
Caffeine consumption	CD14	0.004	3.5%	(1.3%, 5.6%)
Caffeine consumption	IL6R	0.003	2.1%	(0.9%, 3.3%)
Caffeine consumption	MTHFSD	0.001	1.2%	(0.5%, 1.8%)
Short Sleep Duration	CHL1	0.015	7.3%	(0.3%, 14.3%)
Insomnia	PCSK9	0.008	10.0%	(0.5%, 19.5%)
Sports	VIM	0.054	36.8%	(16.4%, 57.2%)
Sports	LGALS2	0.020	13.9%	(3.6%, 24.2%)
Sports	THSD1	0.011	7.2%	(1.6%, 12.8%)
VPA	CNP	0.066	49.9%	(12.3%, 87.4%)
VPA	ARG1	0.036	27.2%	(2.2%, 52.2%)

VPA	TIRAP	0.025	19.0%	(1.5%, 36.5%)
VPA	MTHFSD	0.017	13.2%	(1.6%, 24.8%)
VPA	UGDH	0.017	13.1%	(1.7%, 24.6%)
VPA	SWAP70	0.017	13.1%	(3.6%, 22.5%)
VPA	AGER	0.015	11.3%	(3.3%, 19.2%)
VPA	TIMP3	0.009	6.6%	(2.2%, 11.0%)
VPA	CD14	0.008	6.1%	(1.2%, 11.1%)
Sedentary behaviour	PCSK9	0.035	15.4%	(5.3%, 25.6%)
Sedentary behaviour	MST1	0.022	9.4%	(1.2%, 17.6%)
Sedentary behaviour	APOA5	0.008	3.5%	(0.6%, 6.5%)
Sedentary behaviour	C1S	0.006	2.7%	(0.3%, 5.1%)
Leisure screen time	RPS6KA1	0.019	13.4%	(1.4%, 25.3%)
Leisure screen time	MST1	0.011	7.8%	(0.3%, 15.4%)
Leisure screen time	C1R	0.006	4.6%	(0.4%, 8.8%)
Leisure screen time	C1S	0.004	3.1%	(0.4%, 5.8%)

Note: BMI, body mass index; WHR, waist-to-hip ratio; WC, waist circumference; VAT, visceral adipose tissue; VPA, vigorous physical activity.

Supplemental Table 23. The statistical power of Mendelian randomization analyses.

Exposure	Outcome	Statistical power
Body mass index	coronary artery disease	100%
Waist-to-hip ratio	coronary artery disease	100%
Visceral adipose tissue	coronary artery disease	100%
Waist circumference	coronary artery disease	100%
Smoking initiation	coronary artery disease	100%
Lifetime smoking index	coronary artery disease	100%
Alcohol drinking	coronary artery disease	84%
Alcohol dependence	coronary artery disease	97%
Coffee consumption	coronary artery disease	100%
Caffeine consumption	coronary artery disease	100%
Sports	coronary artery disease	57%
MVPA	coronary artery disease	100%
VPA	coronary artery disease	59%
Sedentary behavior	coronary artery disease	100%
Leisure screen time	coronary artery disease	100%
Sleep duration	coronary artery disease	100%
Short sleep duration	coronary artery disease	100%
Long sleep duration	coronary artery disease	81%
Insomnia	coronary artery disease	100%
Body mass index	MAP1LC3A	99%

Body mass index	ANGPTL4	98%
Body mass index	RPS6KA1	100%
Body mass index	PCSK9	100%
Body mass index	ITPKA	99%
Waist-to-hip ratio	ANGPTL4	77%
Waist-to-hip ratio	RPS6KA1	100%
Waist-to-hip ratio	PCSK9	100%
Waist-to-hip ratio	ITPKA	97%
Waist circumference	RPS6KA1	58%
Waist circumference	PCSK9	84%
Visceral adipose tissue	MAP1LC3A	69%
Visceral adipose tissue	ANGPTL4	95%
Visceral adipose tissue	RPS6KA1	100%
Visceral adipose tissue	PCSK9	100%
Visceral adipose tissue	ITPKA	95%
Sports	LGALS2	55%
VPA	CNP	81%
SB	PCSK9	90%
SB	MST1	100%
LST	RPS6KA1	84%
LST	MST1	100%
Smoking initiation	RGMB	100%
Smoking initiation	PCSK9	88%

Smoking index	ITPKA	81%
Smoking index	PCSK9	93%
Smoking index	RPS6KA1	91%
Coffee consumption	MAP1LC3A	57%
Coffee consumption	FAS	100%

Note: MVPA, moderate to vigorous physical activity; VPA, vigorous physical activity.
